# Supplementary material for: Cellulose nanofiber-mediated manifold dynamic synergy enabling adhesive and photo-detachable hydrogel for self-powered E-skin
Source: Nat Commun. 2024 May 8;15:3859. doi: 10.1038/s41467-024-47986-y (PMC11078967; doi:10.1038/s41467-024-47986-y)
Supplement: Supplementary file 1 — Supplementary Information [file 41467_2024_47986_MOESM1_ESM.docx]

**Supplementary Information for**

**Cellulose Nanofiber-Mediated Manifold Dynamic Synergy Enabling Adhesive and Photo-Detachable Hydrogel for Self-Powered E-Skin**

Lei Zhang^1#^, Lu Chen^2#^, Siheng Wang^1,2^, Shanshan Wang^3^, Dan Wang^1^, Le Yu^2^, Xu Xu^3^, He Liu^1^* & Chaoji Chen^2^*

^1^ Jiangsu Key Laboratory of Biomass Energy and Material, Institute of Chemical Industry of Forest Products, Chinese Academy of Forestry, 210042 Nanjing, China.

^2^ Hubei Biomass-Resource Chemistry and Environmental Biotechnology Key Laboratory, School of Resource and Environmental Sciences, Wuhan University, 430079 Wuhan, China.

^3^ Jiangsu Co–Innovation Center of Efficient Processing and Utilization of Forest Resources, College of Chemical Engineering, Nanjing Forestry University, 210037 Nanjing, China.

^#^ Equal contributions.

* Corresponding authors:

He Liu, email: liuhe.caf@gmail.com.

Chaoji Chen, email: chenchaojili@whu.edu.cn.

This file includes

**Supplementary Methods**

**Suppl. Fig. 1.** Schematic illustration of the photo-detachable mechanism of the cellulose nanofiber reinforced dopamine-poly(acrylic acid)-Fe^3+^ (CNF-DA/PAA@Fe^3+^) hydrogel based on the photo-Fenton-like (P.F.) reaction.

**Suppl. Fig. 2.** Schematic diagrams illustrating the mechanism of the reversible tunability of the supramolecular network in the CNF-DA/PAA@Fe^3+^ hydrogel based on the P.F. reaction.

**Suppl. Fig. 3.** Schematic diagrams illustrating the peeling process of the CNF-DA/PAA@Fe^3+^ hydrogel from the substrate under non-UV and UV exposure.

**Suppl. Fig. 4.** The digital image of the CNF-DA/PAA@Fe^3+^ hydrogels prepared with different Fe^3+^ contents.

**Suppl. Fig. 5.** The mechanical properties of CNF-DA/PAA@Fe^3+^ hydrogels with different Fe^3+^ contents.

**Suppl. Fig. 6.** The adhesive performance of CNF-DA/PAA@Fe^3+^ hydrogels with different Fe^3+^ contents.

**Suppl. Fig. 7.** Microstructural images of the CNF-DA/PAA@Fe^3+^ hydrogel.

**Suppl. Fig. 8.** One-dimensional Raman spectra of the CNF-DA/PAA@Fe^3+^ hydrogel.

**Suppl. Fig. 9.** Photographs of the different solutions during the UV irradiation and air oxidation process.

**Suppl. Fig. 10.** X-Ray diffraction (XRD) spectroscopy of the CNF-DA/PAA@Fe^3+^ hydrogel before and after UV irradiation.

**Suppl. Fig. 11.** The electron paramagnetic resonance (EPR) spectra of the CNF-Fe^3+^ solution after UV irradiation using the spin trap 5,5-dimethyl-1-pyrroline-N-oxide (DMPO).

**Suppl. Fig. 12.** Photo-detachable mechanism of the CNF-DA/PAA@Fe^3+^ hydrogel via the light-driven supramolecular network engineering.

**Suppl. Fig. 13.** The binding energy between Fe^3+^ or Fe^2+^ and hydroxy groups in CNF chains and CNF-DA chains based on Gaussian calculation.

**Suppl. Fig. 14.** Cyclic mechanical properties of the CNF-DA/PAA@Fe^3+^ hydrogel.

**Suppl. Fig. 15.** Mechanical stabilities of the CNF-DA/PAA@Fe^3+^ hydrogel with different air oxidation times.

**Suppl. Fig. 16.** Self-healing properties of the CNF-DA/PAA@Fe^3+^ hydrogel.

**Suppl. Fig. 17.** Thermal and rheological behavior of the CNF-DA/PAA@Fe^3+^ hydrogel.

**Suppl. Fig. 18.** Comparison of mechanical properties of CNF-DA/PAA@Fe^3+^ hydrogels during the UV irradiation and air-oxidation process.

**Suppl. Fig. 19.** Comparison of mechanical properties of different hydrogels.

**Suppl. Fig. 20.** The mechanical properties of the CNF-DA/PAA@Fe^3+^ hydrogels prepared with different water contents.

**Suppl. Fig. 21.** The mechanical properties of the CNF-DA/PAA hydrogels (without Fe ions) with different water contents.

**Suppl. Fig. 22.** The mechanical properties of the CNF-DA/PAA@Fe^3+^ hydrogels prepared with different CNF-DA contents before and after compression (50% for water content).

**Suppl. Fig. 23.** Photographs of the two same pieces of various representative substrates adhered by the CNF-DA/PAA@Fe^3+^ hydrogel to support a weight of 500 g.

**Suppl. Fig. 24.** The standard 90-degree peel test of the CNF/PAA@Fe^3+^ hydrogel, PAA@Fe^3+^ hydrogel, and PAA hydrogel.

**Suppl. Fig. 25.** Setups for mechanical testing of adhesion performance of the CNF-DA/PAA@Fe^3+^ hydrogel.

**Suppl. Fig. 26.** Comparison of adhesion force of the CNF-DA/PAA@Fe^3+^ hydrogel to different substrates.

**Suppl. Fig. 27.** Comparison of adhesion force of the CNF-DA/PAA@Fe^3+^ hydrogel during the UV irradiation and air-oxidation process on the freshly excised porcine skin.

**Suppl. Fig. 28.** Comparison of the adhesion energy and strain of different hydrogels.

**Suppl. Fig. 29.** The digital images of the CNF-DA/PAA@Fe^3+^ hydrogels with different water contents.

**Suppl. Fig. 30.** The adhesive force-displacement curves of the CNF-DA/PAA@Fe^3+^ hydrogels prepared with different water contents on the freshly excised porcine skin.

**Suppl. Fig. 31.** The adhesive force-displacement curves of the CNF-DA/PAA@Fe^3+^ hydrogels prepared with different water contents on the engineering glass.

**Suppl. Fig. 32.** Comparison of the adhesive performance of the CNF-DA/PAA@Fe^3+^ hydrogels with different water contents on the skin and glass.

**Suppl. Fig. 33.** Comparison of the adhesive performance of the CNF-DA/PAA@Fe^3+^ hydrogels with different water contents on the glass with other reported works.

**Suppl. Fig. 34.** The adhesive force-displacement curves of the CNF-DA/PAA hydrogels (without Fe ions) with different water contents on the engineering glass.

**Suppl. Fig. 35.** The adhesion strength of the CNF-DA/PAA@Fe^3+^ hydrogel to the freshly excised porcine skin after air-oxidation.

**Suppl. Fig. 36.** Adhesion stability of the hydrogels.

**Suppl. Fig. 37.** The adhesion strength of the CNF-DA/PAA@Fe^3+^ hydrogel to the engineering glass.

**Suppl. Fig. 38.** Adhesion strength as a function of several variables in UV irradiation intensities and lasting time.

**Suppl. Fig. 39.** Adhesion strength as a function of several variables in environment temperatures.

**Suppl. Fig. 40.** Biocompatibility test of the CNF-DA/PAA@Fe^3+^ hydrogel.

**Suppl. Fig. 41.** Schematic diagrams illustrating highly tunable and reversible properties of the CNF-DA/PAA@Fe^3+^ dynamic hydrogel.

**Suppl. Fig. 42.** Structural formula of the Fe^2+^ and gelatin, chitosan, alginate, starch, PAAm, and PVA.

**Suppl. Fig. 43.** Universality of the photo-detachable adhesion strategy.

**Suppl. Fig. 44.** Universality of the CNF-mediated photo-detachable adhesion strategy.

**Suppl. Fig. 45.** Photographs of the photo-detachable CNF-DA/PAA@Fe^3+^ hydrogel to light up LEDs under different conditions.

**Suppl. Fig. 46.** Electrical performance of the CNF-DA/PAA@Fe^3+^ hydrogel.

**Suppl. Fig. 47.** Application of the CNF-DA/PAA@Fe^3+^ hydrogel as a self-powered e-kin for whole-body physiological and motion monitoring.

**Suppl. Fig. 48.** Schematic illustration of fabricating the two-layer structured photo-detachable adhesion-triboelectric nanogenerator (PdA-TENG).

**Suppl. Fig. 49.** Working principle of the single-electrode PdA-TENG with two-layer structure.

**Suppl. Fig. 50.** Schematic design of the PdA-TENG circuit diagram.

**Suppl. Fig. 51.** The electrical output performance of photo-detachable adhesion-triboelectric nanogenerator (PdA-TENG).

**Suppl. Fig. 52.** Water retention properties of the CNF-DA/PAA@Fe^3+^ hydrogel.

**Suppl. Fig. 53.** The voltage output of the PdA-TENG with repeated cycles contacting under UV light.

**Suppl. Fig. 54.** Stability and durability test of the PdA-TENG.

**Suppl. Fig. 55.** PdA-TENG is used as a self-powered e-kin device for whole-body physiological and motion monitoring.

**Suppl. Fig. 56.** AFM image of the CNFs.

**Suppl. Fig. 57.** Typical conductometric titration curve of the CNFs for determining the surface charge density.

**Suppl. Fig. 58.** The structure of CNF, CNF-DA.

**Suppl. Fig. 59.** Fourier transform infrared (FTIR) spectra of CNF-DA/PAA@Fe^3+^ hydrogel, CNF/PAA@Fe^3+^ hydrogel, and PAA@Fe^3+^ hydrogel.

**Suppl. Table. 1.** Comparison of the tunable ratio in adhesive strength of the CNF-DA/PAA@Fe^3+^ hydrogel with other adhesive materials.

**Supplementary References S1-S14**

**Supplementary Methods**

**Synthesis of cellulose nanofiber reinforced poly(acrylic acid)-iron metal (CNF/PAA@Fe^3+^) hydrogel, poly(acrylic acid)-iron metal (PAA@Fe^3+^) hydrogel, poly(acrylic acid) (PAA) hydrogel.** Chemically crosslinked CNF/PAA@Fe^3+^ hydrogel, PAA@Fe^3+^ hydrogel, and pure PAA hydrogel were synthesized via polymerization at 70 °C. The preparation process of the PAA@Fe^3+^ hydrogel solution was the same as that described above, except that CNF-DA was replaced with H_2_O. Only PAA hydrogel was prepared with 1.25 mL acrylic acid (AA), 7 mL deionized water, 1 mg/mL MBA, and 0.0625 mL/mL APS (with respect to the volume of the deionized water) at 90 °C.

**Synthesis of universal hydrogels.** Chemically crosslinked universal hydrogels were synthesized via polymerization at 70 °C. A precursor solution was prepared by adding 2 mg/mL biomass materials (10 wt% for gelatin, chitosan, 6 wt% for starch and 1 wt% for alginate) and petroleum-based polymers (10 wt% for PVA, 1 g PAAm) respectively to a solution containing 5 mL CNF-DA solution, 1.25 mL AA, 0.01 g FeCl_3_, 2 mL DI water, 1 mg/mL chemical cross-linker MBAA and 0.0625 mL/mL thermal initiator APS. The precursor solution was then polymerized under 70-90 °C for 40 min to form the universal hydrogels.

**Mechanical tests.** The tensile and compressive mechanical properties of the hydrogels were measured on a universal testing machine (UTM6503, SANS, China) which was equipped with a 5 kN load cell at room temperature. The tensile tests were conducted on rectangular samples (25 × 5 × 1 mm^3^) at 50 mm min^−1^. The cyclic tensile tests were driven at a tensile speed of 100 mm min^−1^ for ten loading-unloading cycles and a strain of 200% with no intervals between continuous cycles. The compressive tests were conducted with cylindrical samples (a height of 10 mm, a diameter of 10 mm) at the compressive rate of 5 mm min^−1^. The cyclic compressive tests were driven at the compressive speed of 10 mm min^−1^ for ten loading-unloading cycles as well as the strain of 50% with no intervals between continuous cycles. To obtain the tensile properties of self-healed hydrogels, the samples were firstly cut into two halves; secondly, the optical microscopy (Zeiss, Germany) image of the self-healing behavior of the photo-detachable hydrogel was obtained by a digital microscope camera; thirdly, the self-healed hydrogels of the tensile properties were tested by a universal testing machine (UTM6503, SANS, China) with a tensile speed of 50 mm min^−1^ at the room temperature. The rheological properties of the hydrogels were measured using the Thermo Fisher Scientific MARS60 rheometer with cone-plate geometry (cone angle = 1°, plate diameter = 10 mm) within five minutes.

**Biocompatibility test of the cellulose nanofiber reinforced dopamine-poly(acrylic acid)-iron metal (CNF-DA/PAA@Fe^3+^) hydrogel.** L cell, L-929 were purchased from Wuhan Pricella Biotechnology Co., Ltd (CL-0137). After ethanol/UV sterilization in a certified A2 biosafety cabinet, the prepared extracts are placed in standard 96-well cell culture plates and a total of 500,000 L-929 mouse fibroblasts are seeded in each well. Cells are treated with trypsin EDTA (Giboc) and then resuspended with the extract. Cells are seeded into each well (96-well plate) at a density of 1 × 10^3^ and then cultured for 24 hours, 48 hours, and 96 hours. The cytocompatibility of hydrogels is analyzed by cell counting kit-8 (CCK-8) assay (Bimake) and live/dead assay. 2 μM Calcein AM (in DPBS) and 4 μM EthD-1 (Invitrogen) working solution are added to the wells. The 96-well plate is finally incubated for 20 minutes at 37 °C in a 5% CO_2_ incubator. The morphology of the cells is obtained using a fluorescence microscope. 6 parallel experiments are performed for each group.

**Ionic conductivity measurement.** The electrochemical impendence spectra of all hydrogels were tested with an electrochemical workstation (CORRTEST, CS310H, China). The data was obtained by the electrochemical AC impendence spectroscopy (IMP) of the hydrogels at a frequency range from 10^−1^ to 10^−5^ Hz as well as 100 mV voltage under UV-free and UV-irradiated conditions.

The ionic conductivity of all hydrogels was calculated according to the following equation:

$\sigma=\text{ } \frac{L}{RA}$ (1)

where *L* represents the distance between the two probes, *R* represents the electrical resistance of all hydrogels, and *A* represents the cross-sectional area of the hydrogels.

**Supplementary Figures**


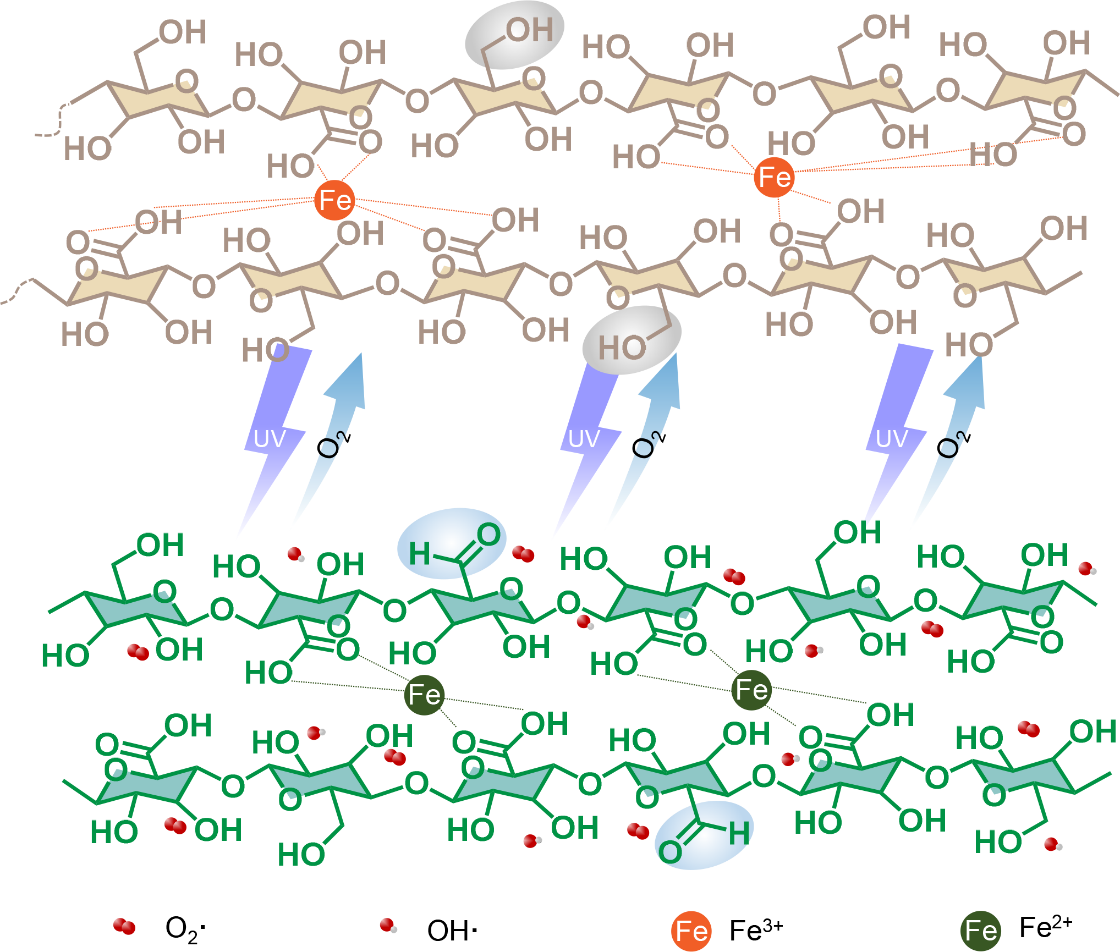


**Supplementary Fig. 1 | Schematic illustration of the photo-detachable mechanism of the cellulose nanofiber reinforced dopamine-poly(acrylic acid)-iron metal (CNF-DA/PAA@Fe^3+^) hydrogel based on the photo-Fenton-like (P.F.) reaction.** Initially, the six-coordinated complexes between Fe^3+^ and −COOH groups are obtained in the CNF-DA/PAA@Fe^3+^ hydrogel. When exposed to UV irradiation, Fe^3+^ is reduced to Fe^2+^ in the P.F. reaction, and the resulting Fe^2+^ has four-coordination interactions with −COOH groups in the hydrogel.


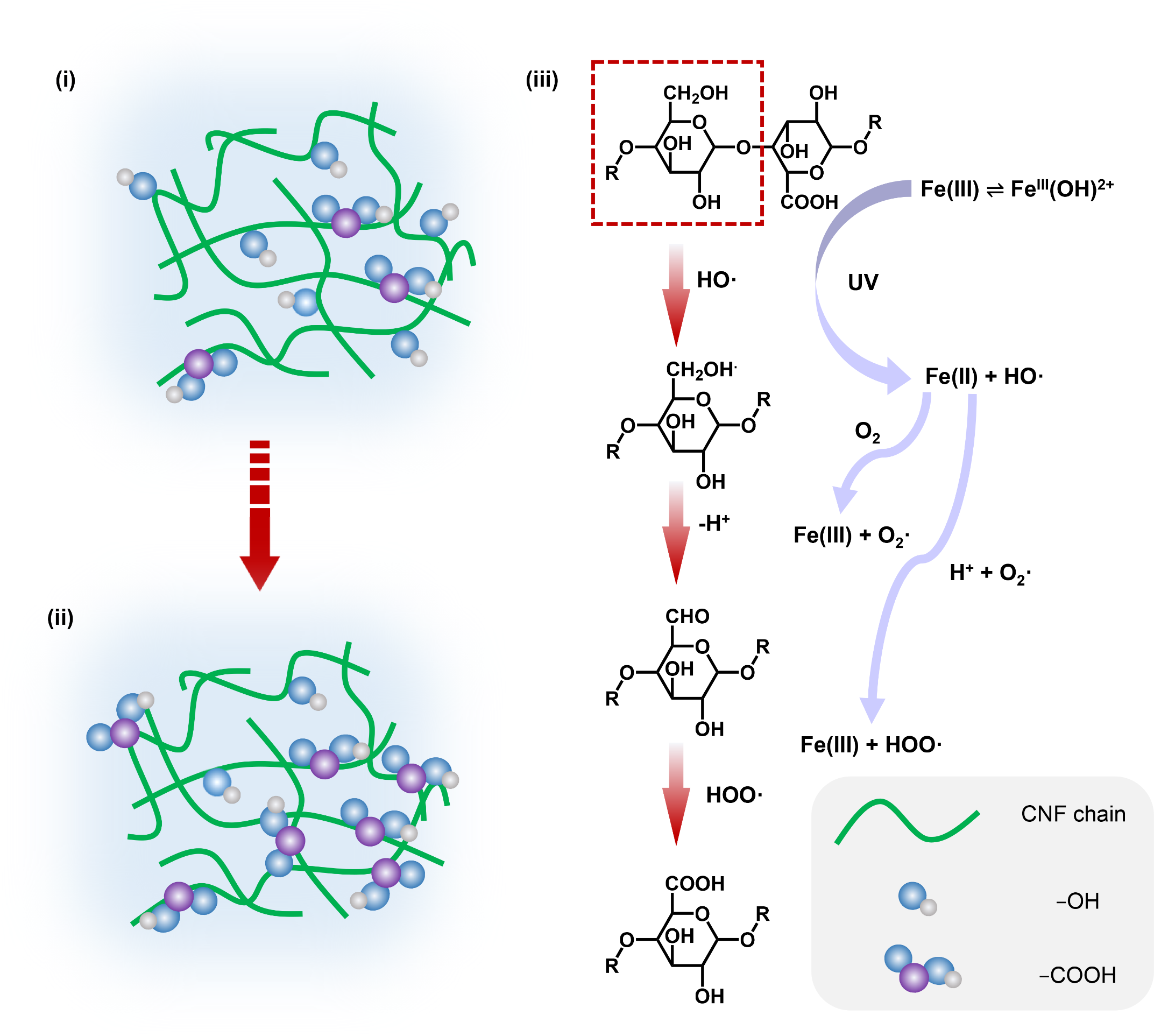


**Supplementary Fig. 2 | Schematic diagrams illustrating the mechanism of the reversible tunability of the supramolecular network in the CNF-DA/PAA@Fe^3+^ hydrogel based on the P.F. reaction.** During the P.F. oxidation process, various free radicals including hydroxyl radicals (HO⋅) and hydroperoxide radicals (HOO⋅) are generated. Fe^3+^ is reduced to Fe^2+^ under UV irradiation, and Fe^2+^ can be converted into Fe^3+^ under air oxidation^1−4^.


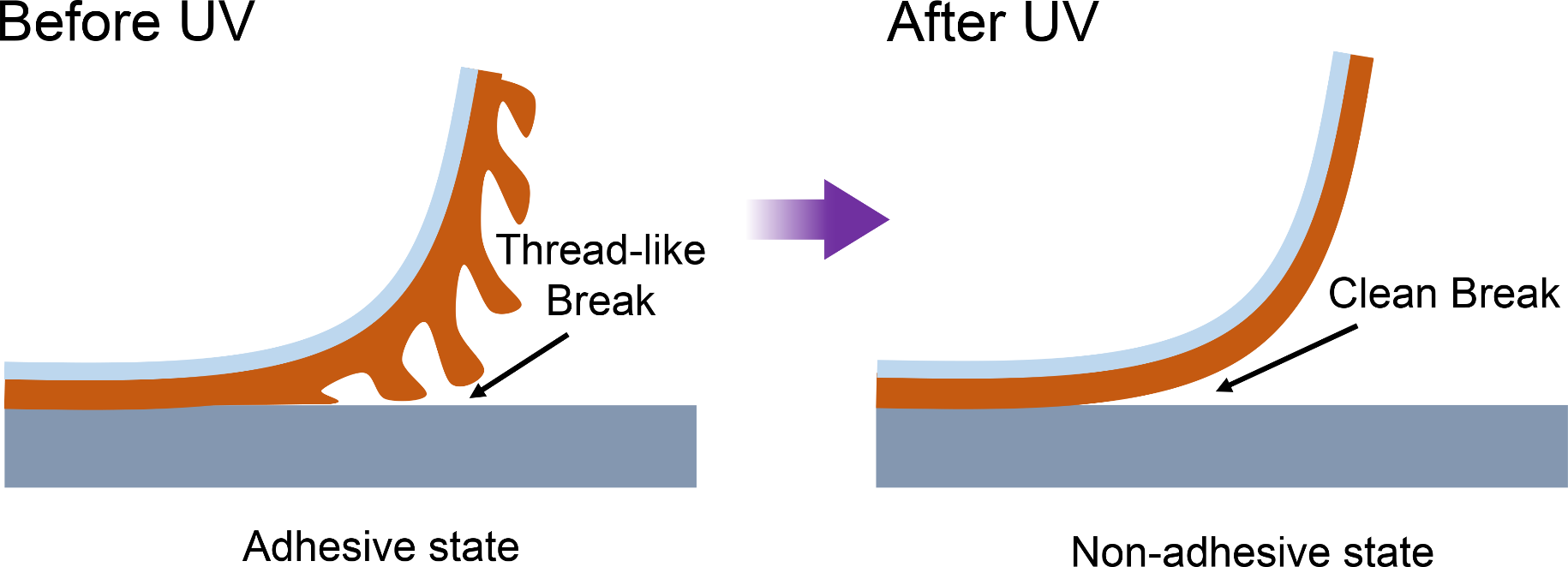


**Supplementary Fig. 3 | Schematic diagrams illustrating the peeling process of the CNF-DA/PAA@Fe^3+^ hydrogel from the substrate under non-UV and UV exposure.** Initially, peeling from strong adhesion is hard, and sometimes painful; when stimulated by UV light, the adhesive CNF-DA/PAA@Fe^3+^ hydrogel is easy to be peeled from the substrate with benign detachment.

**
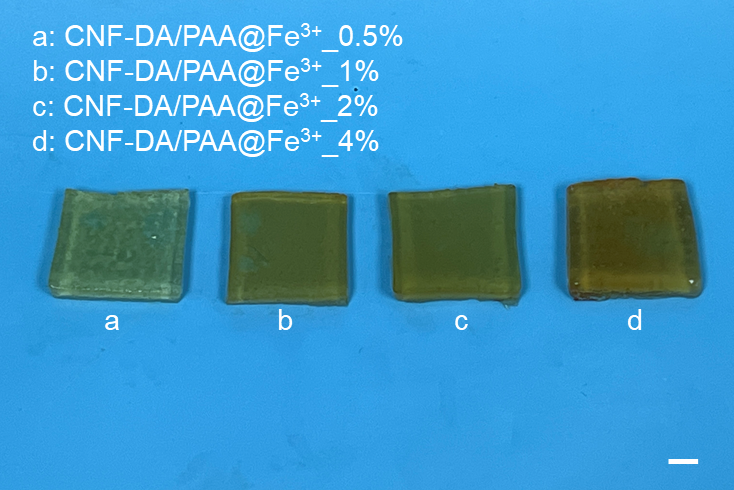
**

**Supplementary Fig. 4 | The digital image of the CNF-DA/PAA@Fe^3+^ hydrogels prepared with different Fe^3+^ ratios.** It can be observed that the overall color of the hydrogels with the same thickness changes from light yellow to dark brown with the gradual increase of Fe^3+^ content in the various CNF-DA/PAA@Fe^3+^ hydrogel. Scale bar, 0.5 cm.

**
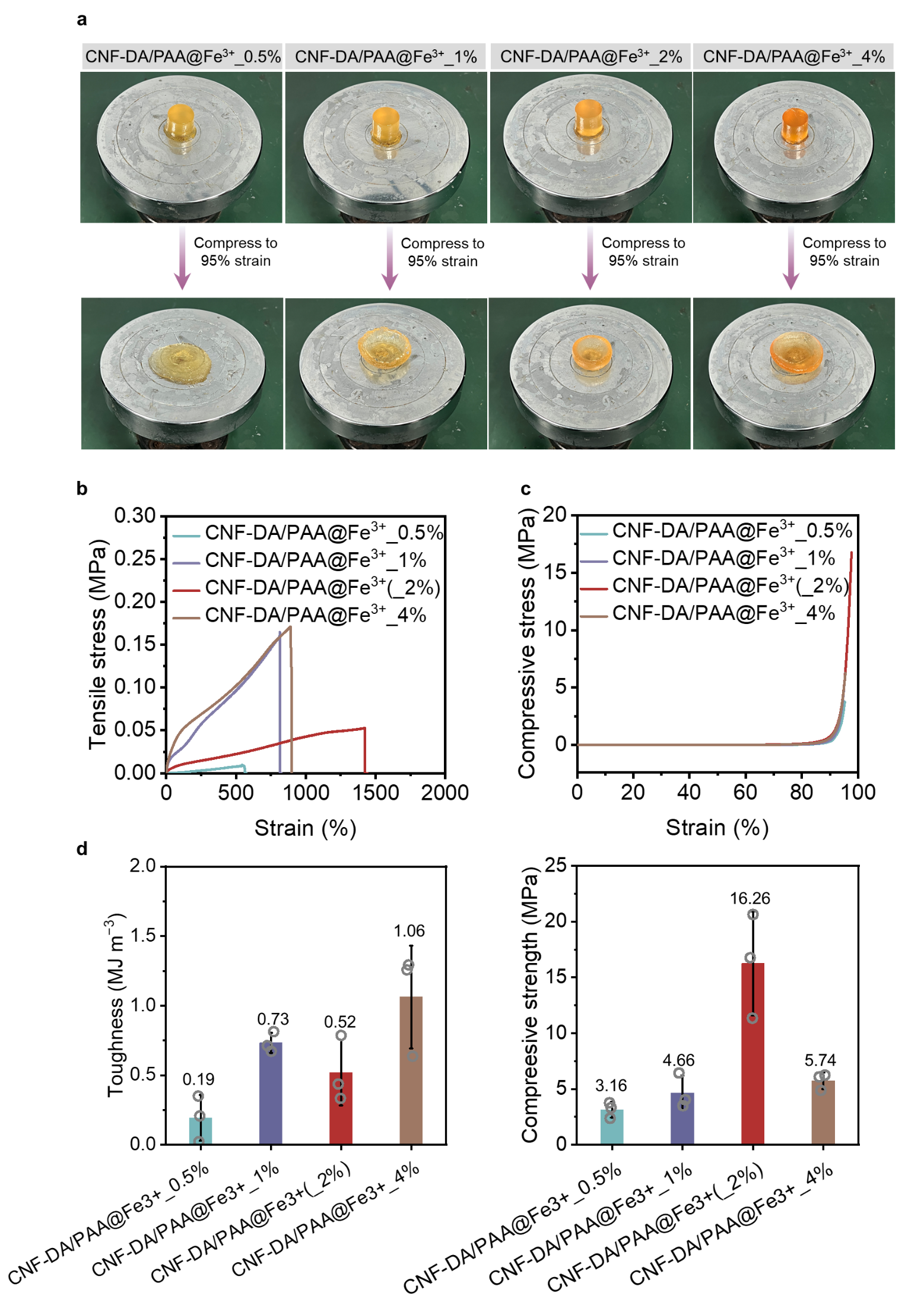
**

**Supplementary Fig. 5 | The mechanical properties of CNF-DA/PAA@Fe^3+^ hydrogels with different Fe^3+^ contents. a** The digital images of the CNF-DA/PAA@Fe^3+^ hydrogels before and after compression. **b** Tensile stress-strain curves of the CNF-DA/PAA@Fe^3+^ hydrogels with different Fe contents. **c** Compressive stress-strain curves of the CNF-DA/PAA@Fe^3+^ hydrogels with different Fe contents. **d** Comparison of toughness and compressive strength of the CNF-DA/PAA@Fe^3+^ hydrogels with different Fe contents. Data in **d** are reported as their menas ±SDs from *n* = 3 independent samples.

As shown in Supplementary Fig. 5a, the CNF-DA/PAA@Fe^3+^_0.5% hydrogel is flattened and has small cracks in the hydrogel center after being compressed under the action of external force, showing significant irrecoverability and poor mechanical properties. In sharp contrast, CNF-DA/PAA@Fe^3+^(_2%) hydrogel maintains high integrity without distinct fracture and cracks under external force despite larger deformation, indicating its excellent mechanical strength and good recoverability. These impressive physical behaviors suggest that the corporation of Fe^3+^ has a positive effect on enhancing the mechanical strength of the CNF-DA/PAA@Fe^3+^ hydrogel.

To further explore the Fe^3+^ on the mechanical properties of hydrogels, we performed the quantitive analysis of these hydrogels in tensile and compressive tests. As shown in Supplementary Fig. 5b, the ultimate stress of the CNF-DA/PAA@Fe^3+^_0.5% hydrogel is 0.0097 MPa at a maximum fracture strain of 562.66%. It can be seen that both the maximum tensile stress and strain of the hydrogel show an improving trend with the increase of Fe^3+^ contents. In particular, when the mass of doped Fe^3+^ accounted for 2% of the mass of the CNF-DA, the resultant CNF-DA/PAA@Fe^3+^(_2%) hydrogel exhibits a fracture strain of 1425% and maximum stress of 0.053 MPa, showing 2.5-fold and 82% increase compared to the CNF-DA/PAA@Fe^3+^_0.5% hydrogel, suggesting a positive role of Fe^3+^ for the mechanical properties of the developed hydrogel. Meanwhile, the toughness of CNF-DA/PAA@Fe^3+^(_2%) hydrogel is 0.52 MJ m^−3^, respectively, which is 2.7 times of the corresponding CNF-DA/PAA@Fe^3+^_0.5% hydrogel (0.19 MJ m^−3^), implying a significant improved mechanical toughness. However, the tensile properties and toughness of the obtained hydrogels decrease when Fe^3+^ content is continuously added, which is due to the heterogeneous system caused by excess Fe^3+^.

Furthermore, this interesting mechanical behavior concerning the positive effect of Fe^3+^ was also found on the compressive performance of the hydrogel, that is, with the increase of Fe^3+^ content, the compressive strength of the hydrogel showed a trend of first increasing and then decreasing (Supplementary Fig. 5c). Especially, the compressive strength of the CNF-DA/PAA@Fe^3+^(_2%) hydrogel is 16.26 MPa, an increase of 5.2 times compared to that of 3.16 MPa for the CNF-DA/PAA@Fe^3+^_0.5% hydrogel (Supplementary Fig. 5d), indicating the increase of Fe^3+^ content improves the compressive strength, which is also good agreement with the results of the mechanical demonstration in Supplementary Fig. 5.

**
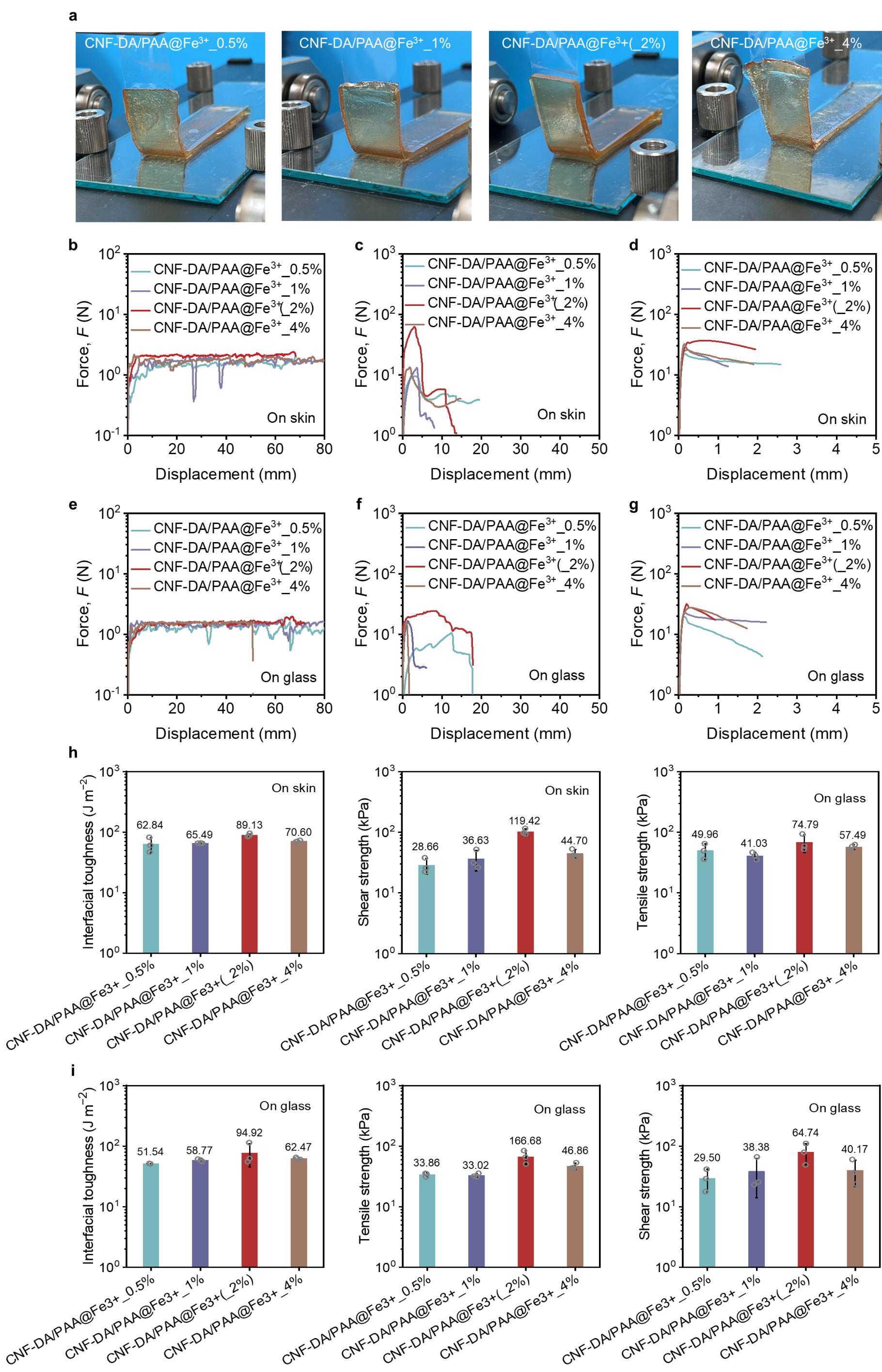
**

**Supplementary Fig. 6 | The adhesive performance of CNF-DA/PAA@Fe^3+^ hydrogels with different Fe^3+^ contents. a** The digital images of the CNF-DA/PAA@Fe^3+^ hydrogels peel from engineering glass. **b−d** 90-degree peel, lap-shear, and tensile force-displacement curves of the CNF-DA/PAA@Fe^3+^ hydrogels prepared with different material ratios on the substrate of freshly excised porcine skin. **e−g** 90-degree peel, lap-shear, and tensile force-displacement curves of the CNF-DA/PAA@Fe^3+^ hydrogels prepared with different material ratios on the substrate of engineering glass. **h, i** Comparison of interfacial toughness, shear strength, and tensile strength of the CNF-DA/PAA@Fe^3+^ hydrogels prepared with different material ratios on the substrates of skin and glass. Data in **h**, **i** are reported as their means ±SDs from *n* = 3 independent samples.

To explore the adhesive properties of various CNF-DA/PAA@Fe^3+^ hydrogels with different Fe contents, we performed 90-degree peeling, lap-shear, and tensile tests on the substrates of freshly excised porcine skin and engineering glass. As shown in Supplementary Fig. 6a, the CNF-DA/PAA@Fe^3+^_0.5% hydrogel can be easily removed from the engineering glass, showing a weak adhesive strength. In sharp contrast, the CNF-DA/PAA@Fe^3+^(_2%) hydrogel exhibits a pull on the glass observed without visible fracture when detached from the glass, indicating an enhanced adhesive strength and mechanical toughness because CNF-DA and Fe^3+^ form a strong coordination interaction and reinforced the interface interaction. However, the CNF-DA/PAA@Fe^3+^_4% hydrogel presents a decayed peeling behavior compared to the CNF-DA/PAA@Fe^3+^(_2%) hydrogel, since an excess of Fe^3+^ leads to a non-uniform network system.

Furthermore, we quantitatively evaluated the adhesive properties of prepared hydrogels with different CNF-DA contents by 90-degree peeling, lap-shear, and tensile tests on the substrates of freshly excised porcine skin and engineering glass (Supplementary Fig. 6b−g). According to these force-displacement curves, we can find that, when the mass ratio of added Fe^3+^ to CNF-DA is less than 2%, as the Fe^3+^ content increases, the peel force, shear force, and tensile force all show a rising trend, which because that the introduction of Fe^3+^ has a positive effect on the cohesive energy of the hydrogel, facilitating more active groups to bond with functional groups on the substrates of skin surface and glass. As expected, the peeling force, shear force, and tensile force of hydrogels show an attenuation when Fe^3+^ is continued to be added, which is ascribed to the excess Fe^3+^ resulting in a non-uniform hydrogel, enabling it easily friable to a weak adhesion on the skin and glass.

More intuitively, the interfacial toughness, shear strength, and tensile strength of various CNF-DA/PAA@Fe^3+^ hydrogels on the substrates of skin and glass were correspondingly obtained, respectively, and a similar adhesion trend to that of the force-displacement curves could be found (Supplementary Fig. 6h, i). Specifically, the CNF-DA/PAA@Fe^3+^(_2%) hydrogel exhibits 89.13 J m^−2^ for interfacial toughness, 119.42 kPa for shear strength, and 74.79 kPa for shear strength on the skin (94.92 J m^−2^ for interfacial toughness, a 166.68 kPa for shear strength, and a 64.74 kPa for shear strength on the glass). These impressive adhesion observations reveal a CNF-DA/PAA@Fe^3+^ hydrogel with excellent adhesion properties through optimal Fe content, that is, the ratio of Fe^3+^ to the mass of the CNF-DA as 2%.


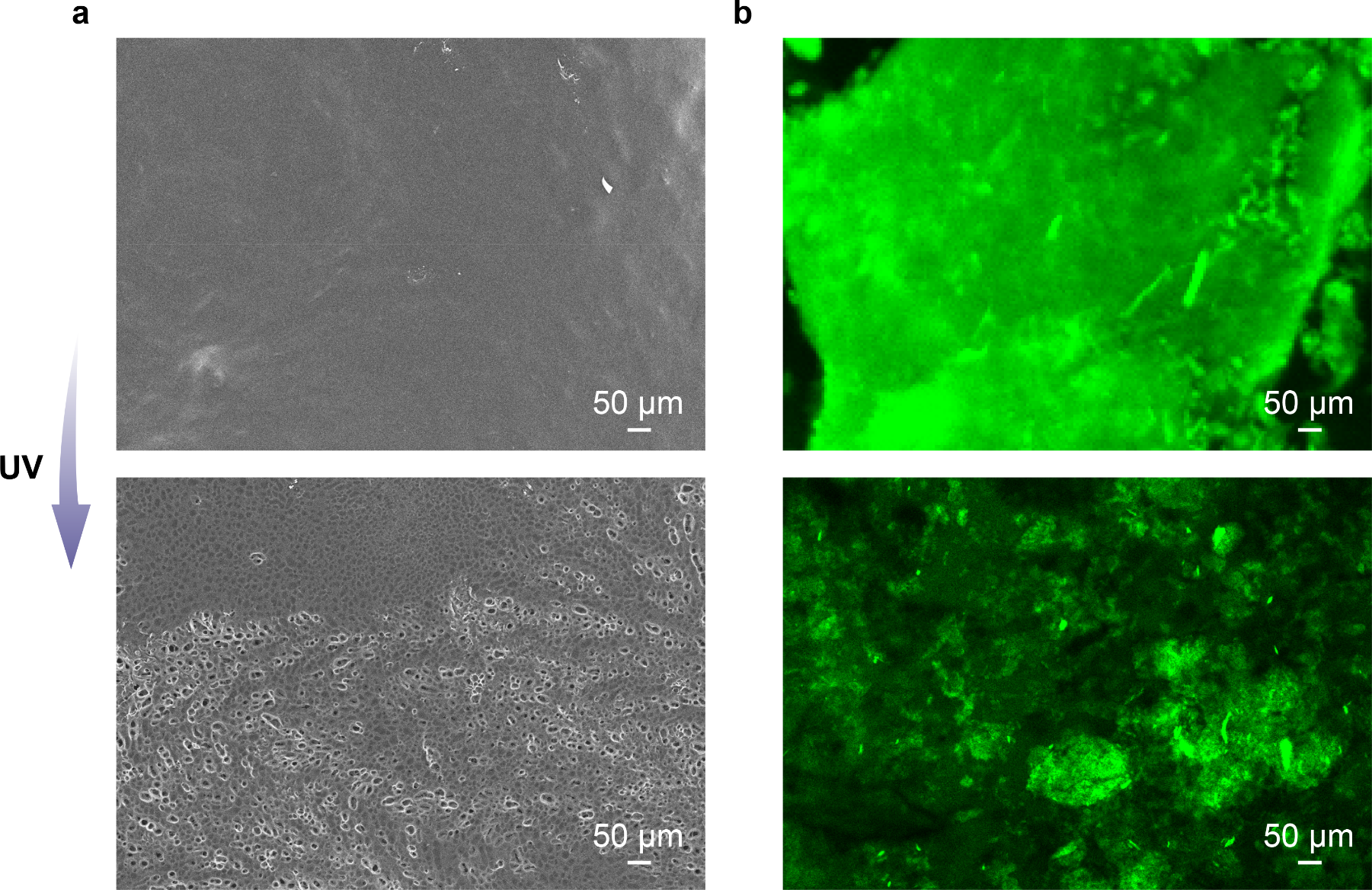


**Supplementary Fig. 7 | Microstructural images of the CNF-DA/PAA@Fe^3+^ hydrogel.** **a** SEM images showing the evolution of the photo-detachable hydrogel before and after UV irradiation. Scale bar, 50 μm. **b** Confocal images showing the microstructures of the photo-detachable hydrogel before and after UV irradiation. Scale bar, 50 μm. The SEM and confocal images exhibit distinct changes in the morphology of CNF-DA/PAA@Fe^3+^ hydrogel from the initial homogeneous dense structure without UV irradiation to the loose porous zone on the surface after the UV irradiation. Scale bar, 50 μm.


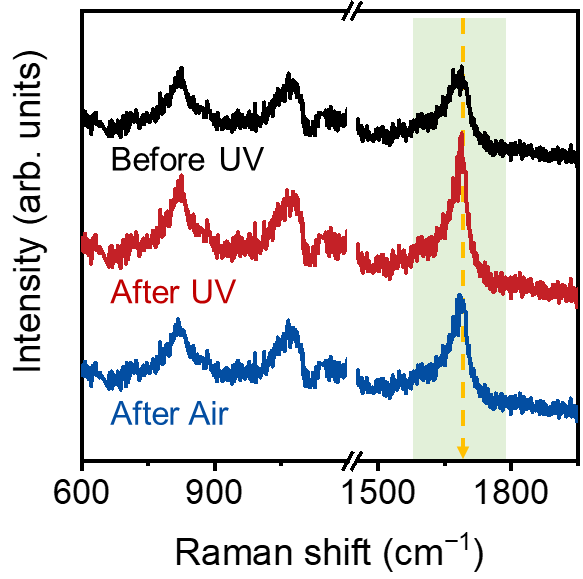


**Supplementary Fig. 8 | One-dimensional Raman spectra of the CNF-DA/PAA@Fe^3+^ hydrogel.**

**
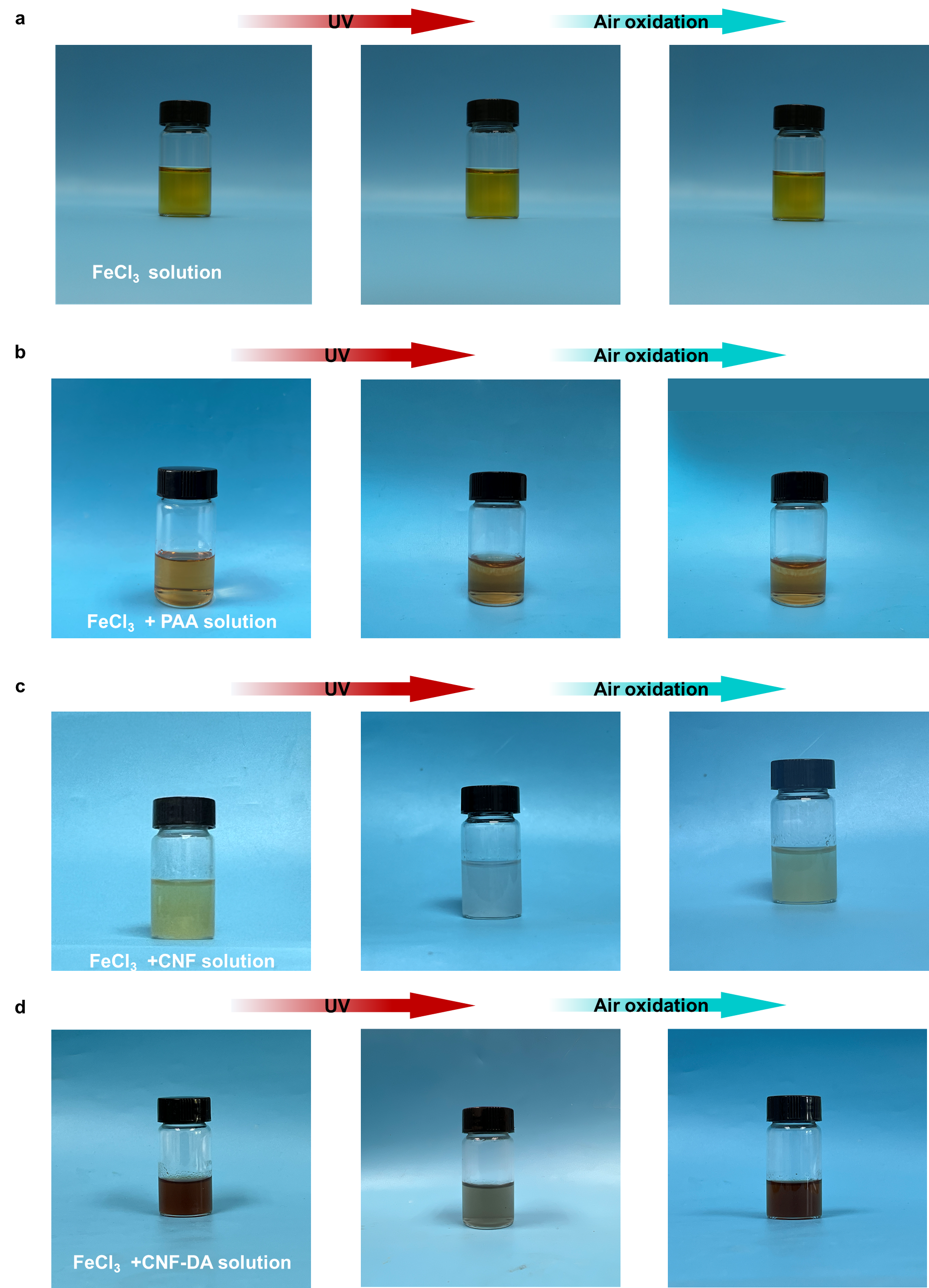
**

**Supplementary Fig. 9 | Photographs of the different solutions during the UV irradiation and air oxidation process.** Initially, both the FeCl_3_, the PAA/FeCl_3_, the CNF/FeCl_3_, and the CNF-DA/FeCl_3_ solutions in brown color are stable. With exposure to UV light and air, the color of the CNF/FeCl_3_ and the CNF-DA/FeCl_3_ solutions change from the initial brown to white and then to brown again. In contrast, the FeCl_3_ the PAA/FeCl_3_ solutions remain in the original state, which indicates the vital role of the CNF in the P.F. reaction.


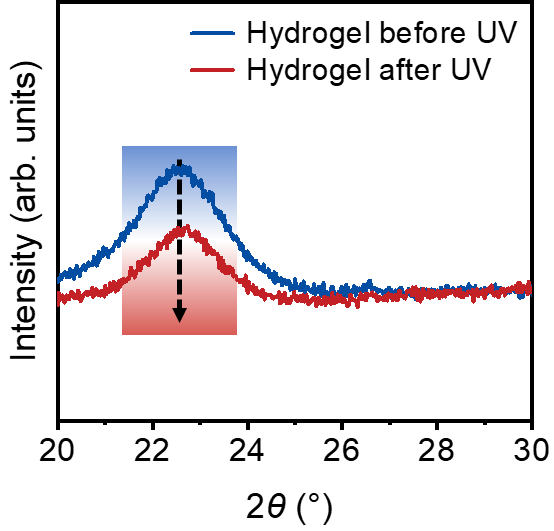


**Supplementary Fig. 10 | X-Ray diffraction (XRD) spectroscopy of the CNF-DA/PAA@Fe^3+^ hydrogel before and after UV irradiation.** The intensity of diffraction peaks around 22° gradually decreases and the waveforms are more flatting in the hydrogel after UV irradiation, indicating the degradation of complexation interaction and thus looser supramolecular network due to Fe ions valence transitions in the CNF-DA/PAA@Fe^3+^ hydrogel when stimulated by UV irradiation.


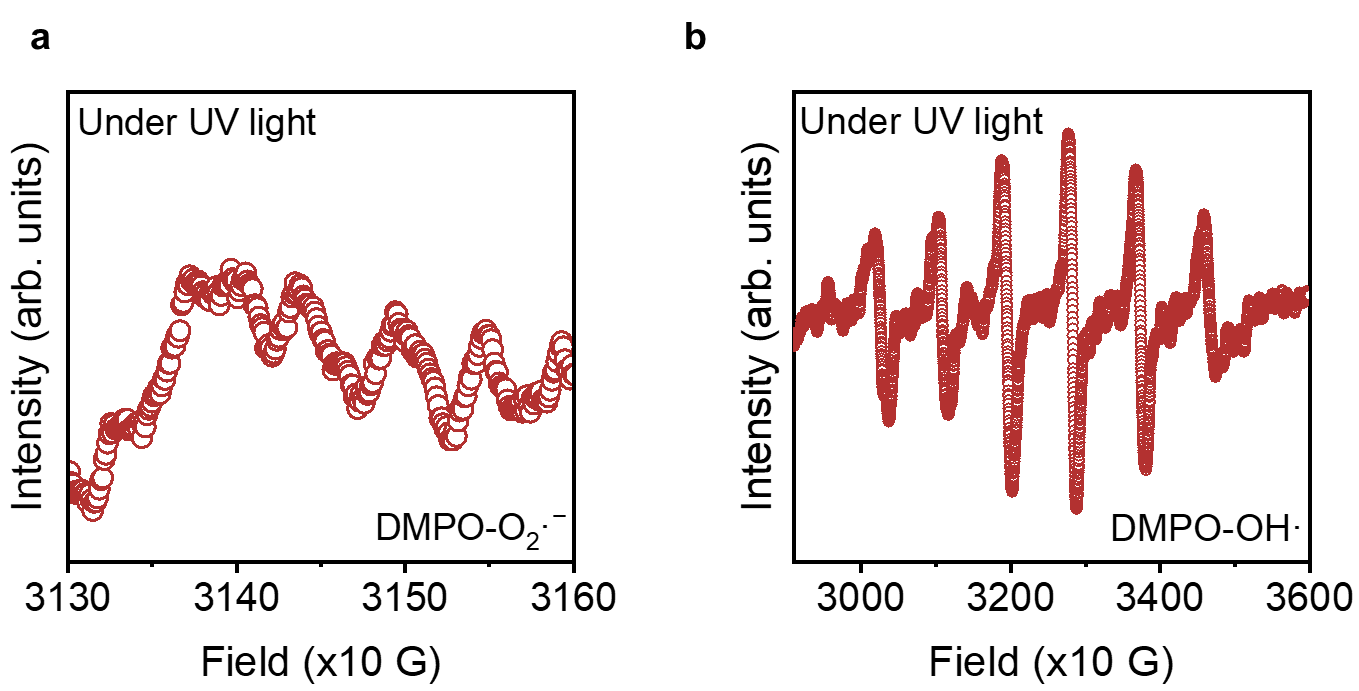


**Supplementary Fig. 11 | The electron paramagnetic resonance (EPR) spectra of the CNF-Fe^3+^ solution after UV irradiation using the spin trap 5,5-dimethyl-1-pyrroline-N-oxide (DMPO).** Adjacent serial peaks assigned to a strong resonance signal of DMPO-O_2_ were observed in the CNF-Fe^3+^ solution, indicating the generation of the O_2_· radical after UV light illumination (Supplementary Fig. 11a)^5^. Meanwhile, the characteristic peaks with a different rough intensity ratio present the formation of OH· radical under UV irradiation (Supplementary Fig. 11b). These observations show that the O_2_· and OH· participate in the P.F. reaction process of CNF-Fe^3+^ solution^6^.
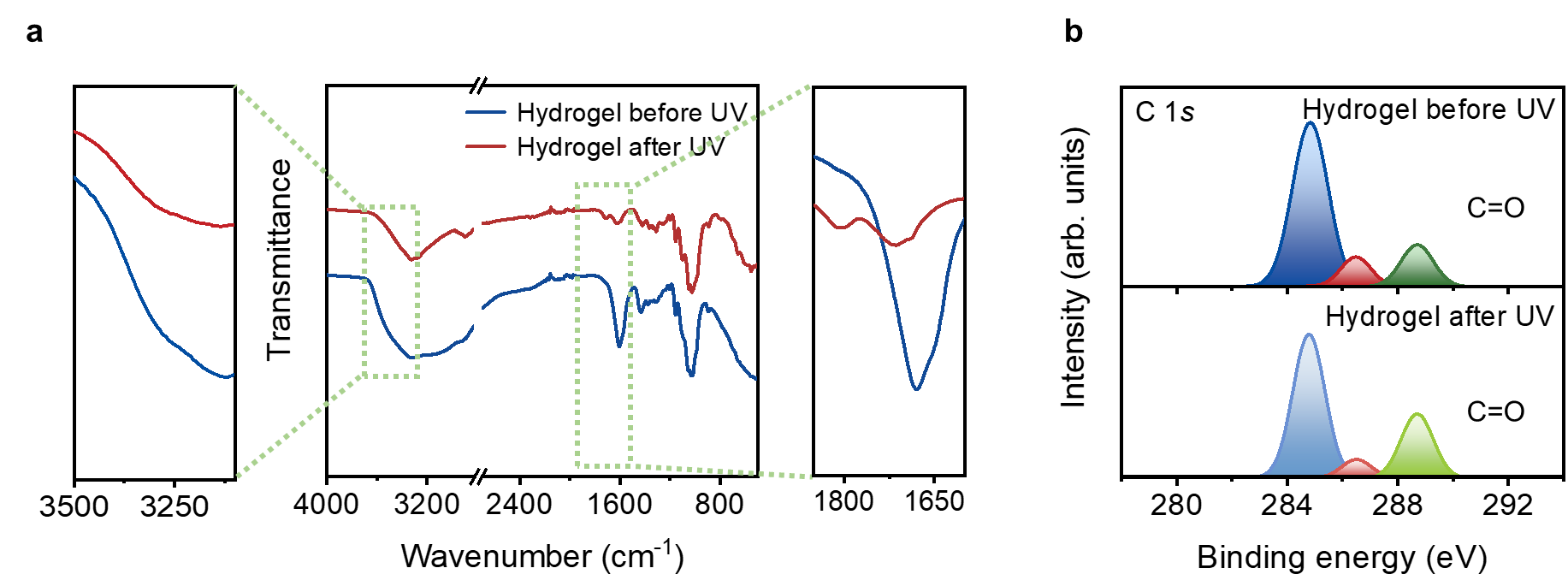


**Supplementary Fig. 12 | Photo-detachable mechanism of the CNF-DA/PAA@Fe^3+^ hydrogel via the light-driven supramolecular network engineering.** **a** Fourier transform infrared (FTIR) spectra of the hydrogel before and after UV irradiation. **b** X-ray photoelectron spectroscopy (XPS) spectra of C 1*s* in the hydrogel before and after UV irradiation.

Carboxyl groups (the characteristic peak at 1682 cm^−1^) of CNF-DA and PAA chains without UV light allow the coordination interactions between Fe^3+^ and carboxyl groups (Supplementary Fig. 12a). Upon exposure to UV light, the position of peaks of carboxyl groups (from 1682 cm^−1^ to 1715 cm^−1^) and hydroxyl groups (from 3150 cm^−1^ to 3315 cm^−1^) slightly shifts, indicating the dissociation of coordination complexes in the CNF-DA/PAA@Fe^3+^ hydrogel^7^. Besides, the higher peak intensity of the C=O in the CNF-DA/PAA@Fe^3+^ hydrogel after UV irradiation than that of before UV irradiation implies that the partial reduction of Fe^3+^ to Fe^2+^ under UV irradiation leads to a destruction of coordination bonding, and thus the supramolecular network crosslinks are further dissociated (Supplementary Fig. 12b).


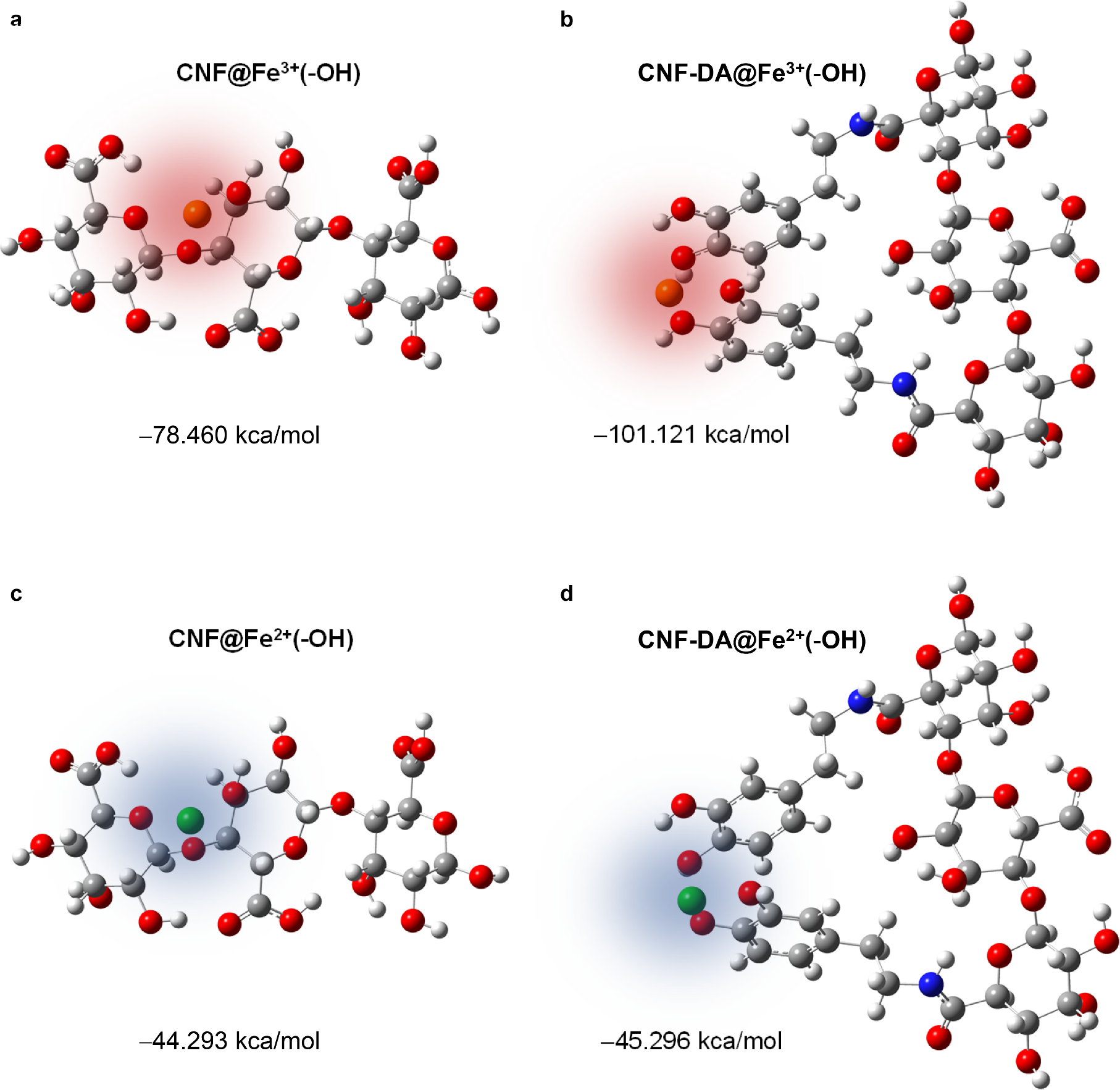


**Supplementary Fig. 13 | The binding energy between Fe^3+^ or Fe^2+^ and hydroxy groups in CNF chains and CNF-DA chains based on Gaussian calculation. a−d** Impact of Fe^3+^, Fe^2+^ respectively, and hydroxy groups of a CNF chain, a CNF-DA chain coordination interaction from Gaussian calculation. In the CNF-DA/PAA@Fe^3+^ hydrogel system, Fe^3+^ ions and catechol groups of CNF-DA form Fe^3+^-catechol coordination interactions, and the hydrogen bonding interaction is also generated between catechol groups. These impressive bonds facilitate a tight network architecture and are consistent with related works^8,9^. Furthermore, under the stimulation of UV light, the weaker Fe^2+^-catechol interactions accompanied by the transformation of Fe^3+^ ions to Fe^2+^ ions lead to a loose supramolecular network, which in turn promotes the easily peeled adhesion behavior for photo-detachable performance.


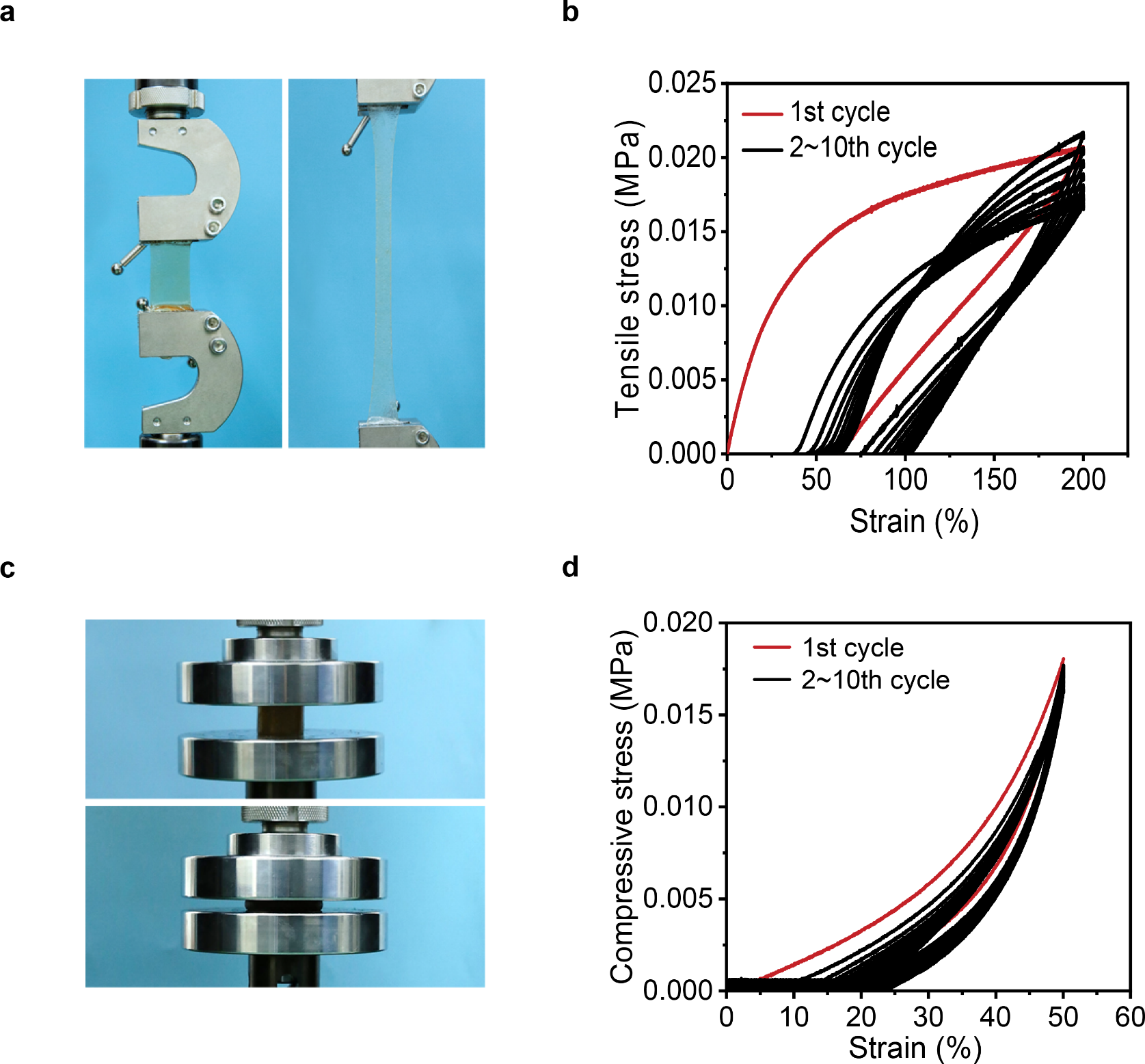


**Supplementary Fig. 14 | Cyclic mechanical properties of the CNF-DA/PAA@Fe^3+^ hydrogel. a** Stretchable photograph of the photo-detachable hydrogel. **b** Cyclic tensile stress-strain curves of the photo-detachable hydrogel. **c** Compressive photographs of the photo-detachable hydrogel. **d** Cyclic compressive stress-strain curves of the photo-detachable hydrogel. The CNF-DA/PAA@Fe^3+^ hydrogel can be stretched 10 times under the external force without breaking, which can recover its original shape immediately after removing the external force, demonstrating the strong stretchability and durability of the CNF-DA/PAA@Fe^3+^ hydrogel.


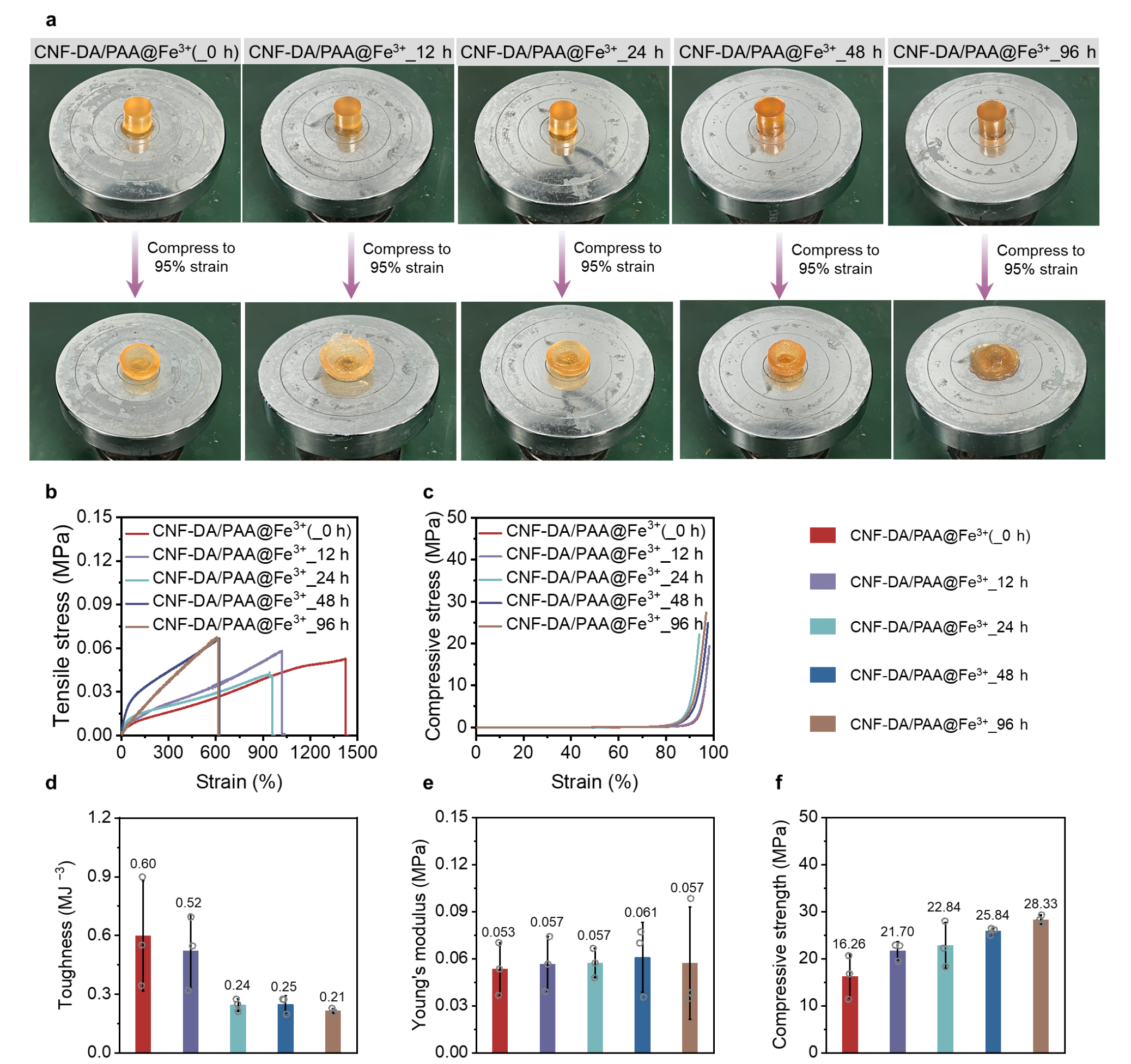


**Supplementary** **Fig.** **15 | Stability of mechanical properties of the CNF-DA/PAA@Fe^3+^ hydrogel with different air oxidation times.** **a** The digital images of the CNF-DA/PAA@Fe^3+^ hydrogels with different times before and after compression. **b** Tensile stress-strain curves of the CNF-DA/PAA@Fe^3+^ hydrogels with different times. **c** Compressive stress-strain curves of the CNF-DA/PAA@Fe^3+^ hydrogels prepared at different times. **d−f** Comparison of the toughness, tensile Young’s modulus, and compressive strength of CNF-DA/PAA@Fe^3+^ hydrogels at different times. Data in **d−f** are reported as their means ±SDs from *n* = 3 independent samples.

As shown in Supplementary Fig. 15a, the initial CNF-DA/PAA@Fe^3+^_0h hydrogel, even after being compressed to extreme deformation (up to 95% compression strain), showed no significant fractures and cracks, indicating excellent mechanical properties. Even with the increase of oxidation time by O_2_, no obvious cracks can be observed, which further proves the mechanical stability of the hydrogel.

Furthermore, tensile and compressive stress-strain tests were conducted to analyze quantitatively the mechanical properties of the hydrogels with prolonged oxidation time. As shown in Supplementary Fig. 15b, the hydrogels exhibit increased tensile stress and decayed tensile strain with extending oxidation time, which is due to the increased crosslinking density of the hydrogel from O_2_ oxidation. In particular, with the oxidation time up to 96 h, the tensile strength and strain of the CNF-DA/PAA@Fe^3+^_96 h hydrogel were 0.066 MPa and 615.46%, respectively. Likewise, similar mechanical behavior is also shown for compressive properties (Supplementary Fig. 15c). Compared with the compressive strength of 16.26 MPa of the CNF-DA/PAA@Fe^3+^_0h hydrogel, the CNF-DA/PAA@Fe^3+^_96h hydrogel correspondingly improves to 28.33 MPa, increased by 1.7 times, respectively (Supplementary Fig. 15d−f). These interesting mechanical observations jointly demonstrate the high strength and stiffness of the hydrogel accompanied by oxidative growth, and it can still maintain good flexibility after being placed in the air for a long time, demonstrating its strong stretchability, durability, and stability.


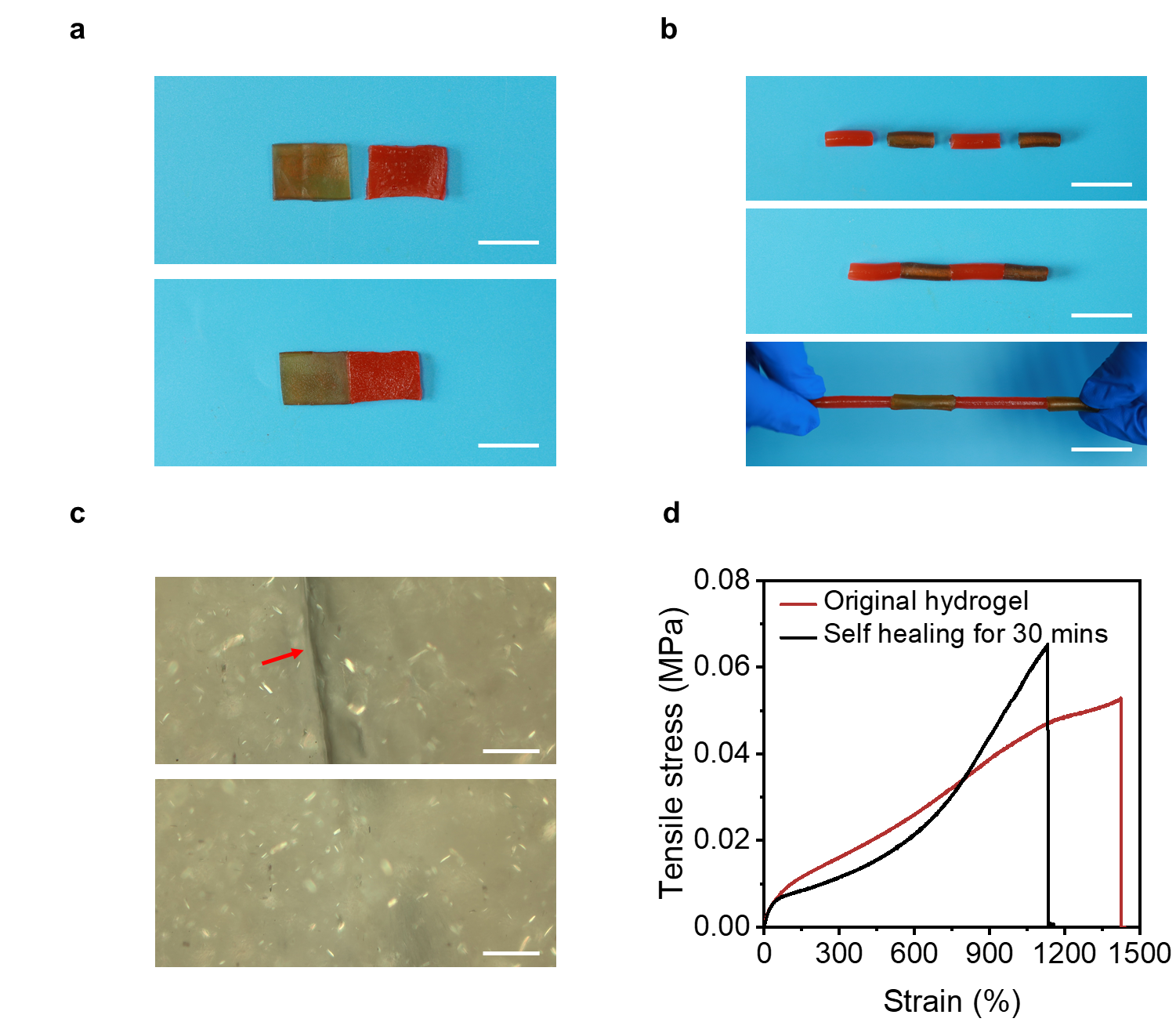


**Supplementary Fig. 16 | Self-healing properties of the CNF-DA/PAA@Fe^3+^ hydrogel.** **a**, **b** The self-healing behavior of the photo-detachable hydrogel. Scale bar, 3 cm. **c** Optical microscopy images of the photo-detachable hydrogel before and after self-healing. Scale bar, 200 μm. **d** Representative tensile stress-strain curves of the hydrogel before and after self-healing. Due to the dynamic and reversible metal coordination, the CNF-DA/PAA@Fe^3+^ hydrogel can still restore its original intact shape after being torn apart, presenting its good self-healing performance.


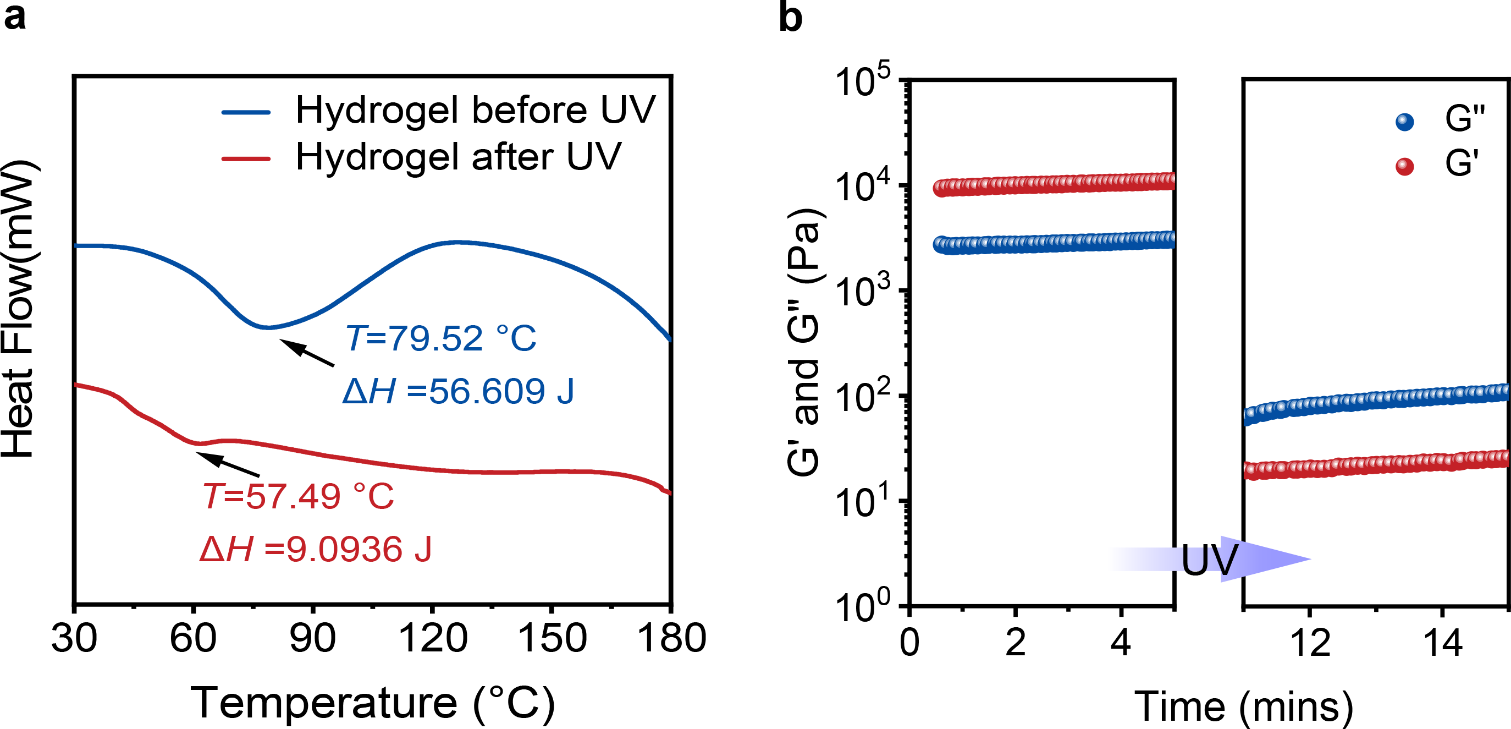


**Supplementary Fig. 17 | Thermal and rheological behavior of the CNF-DA/PAA@Fe^3+^ hydrogel. a** Differential scanning calorimetry (DSC) measurement of the hydrogel before and after UV irradiation. **b** In-situ rheological behavior of the hydrogel in a continuous process of UV irradiation and air oxidation.


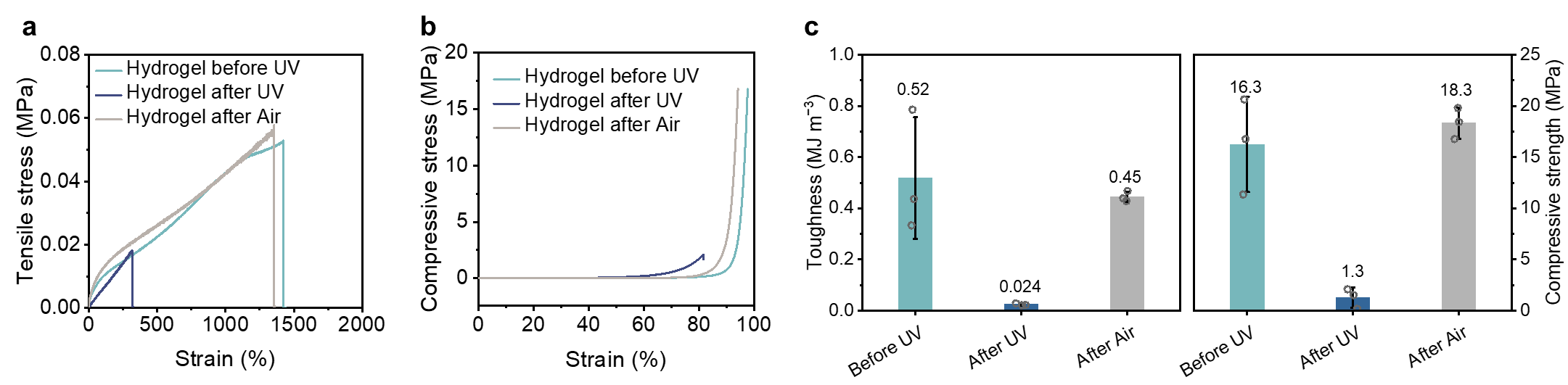


**Supplementary Fig. 18 | Comparison of mechanical properties of CNF-DA/PAA@Fe^3+^ hydrogels during the UV irradiation and air-oxidation process.** **a** Tensile stress-strain curves of the CNF-DA/PAA@Fe^3+^ hydrogels during the UV irradiation and air-oxidation process. **b** Compressive stress-strain curves of the CNF-DA/PAA@Fe^3+^ hydrogels during the UV irradiation and air-oxidation process. **c** Comparison of the toughness and compressive strength of the CNF-DA/PAA@Fe^3+^ hydrogels during the UV irradiation and air-oxidation process. Data in **c** are reported as their means ±SDs from *n* = 3 independent samples.

The CNF-DA/PAA@Fe^3+^ hydrogel exhibits the maximum tensile stress of 0.053 MPa at a fracture strain of 1425%, 66% higher than those of the hydrogel after UV irradiation, which then recover basically when exposure to the air (Supplementary Fig. 18a). The CNF-DA/PAA@Fe^3+^ hydrogel presents a high compressive strength of 16.8 MPa at extreme compressibility up to 95% strain, 7.3 times higher than the hydrogel after UV irradiation (2.3 MPa), and further then recover basically when exposure to the air (Supplementary Fig. 18b). These results demonstrate the tunability of the CNF-DA/PAA@Fe^3+^ hydrogel in terms of the mechanical properties during UV-stimulus process due to the photo-induced Fe^3+^ to Fe^2+^ conversion to tune the supramolecular network structure, and further recover basically when exposure to the air.


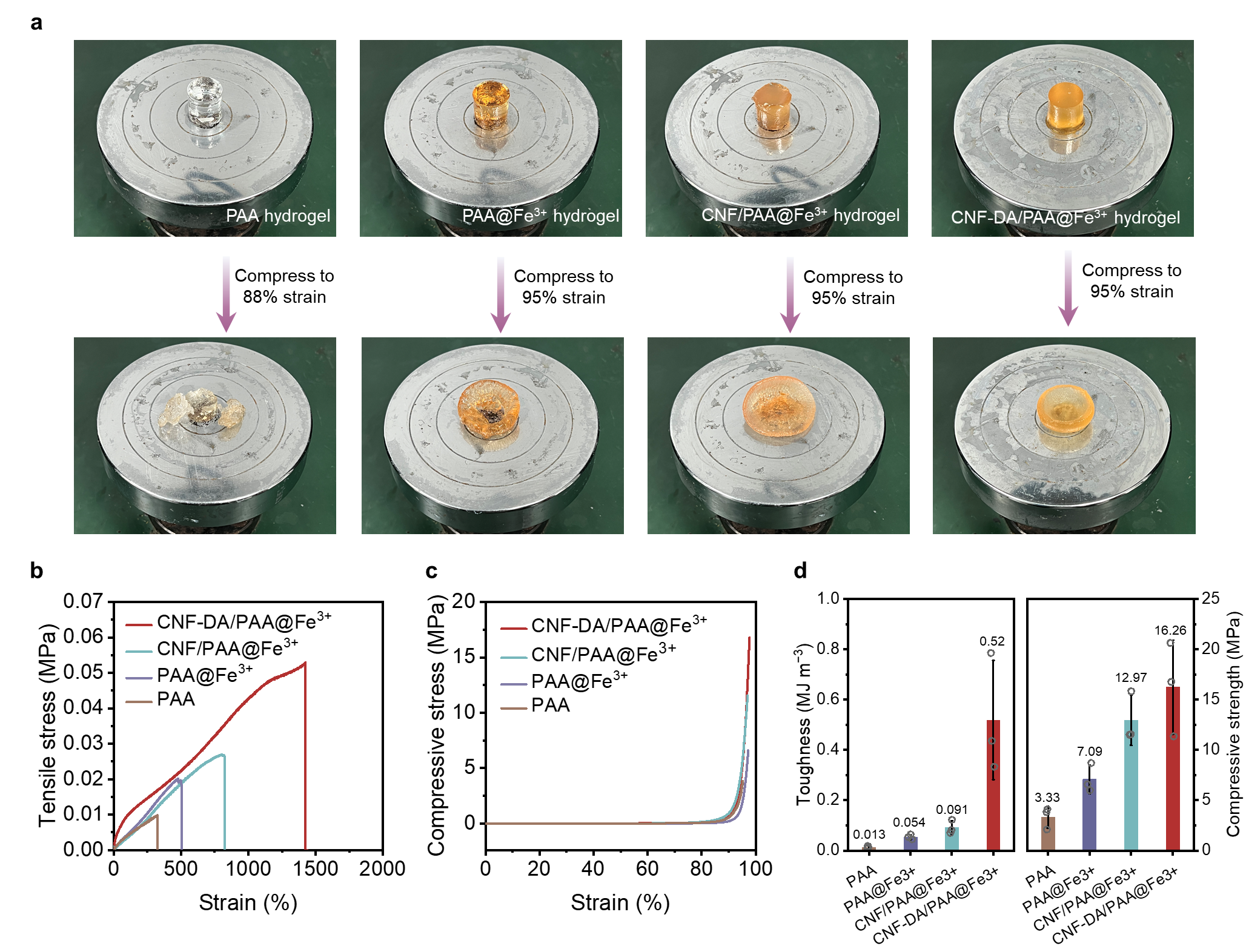


**Supplementary Fig. 19 | Comparison of mechanical properties of different hydrogels. a** The digital images of the PAA, PAA@Fe^3+^, CNF/PAA@Fe^3+^, and CNF-DA/PAA@Fe^3+^ hydrogels before and after compression. **b** Tensile stress-strain curves of the PAA, PAA@Fe^3+^, CNF/PAA@Fe^3+^, and CNF-DA/PAA@Fe^3+^ hydrogels. **c** Compressive stress-strain curves of the PAA, PAA@Fe^3+^, CNF/PAA@Fe^3+^, and CNF-DA/PAA@Fe^3+^ hydrogels. **d** Comparison of the toughness and compressive strength of PAA, PAA@Fe^3+^, CNF/PAA@Fe^3+^, and CNF-DA/PAA@Fe^3+^ hydrogels. Data in **d** are reported as their means ±SDs from *n* = 3 independent samples.


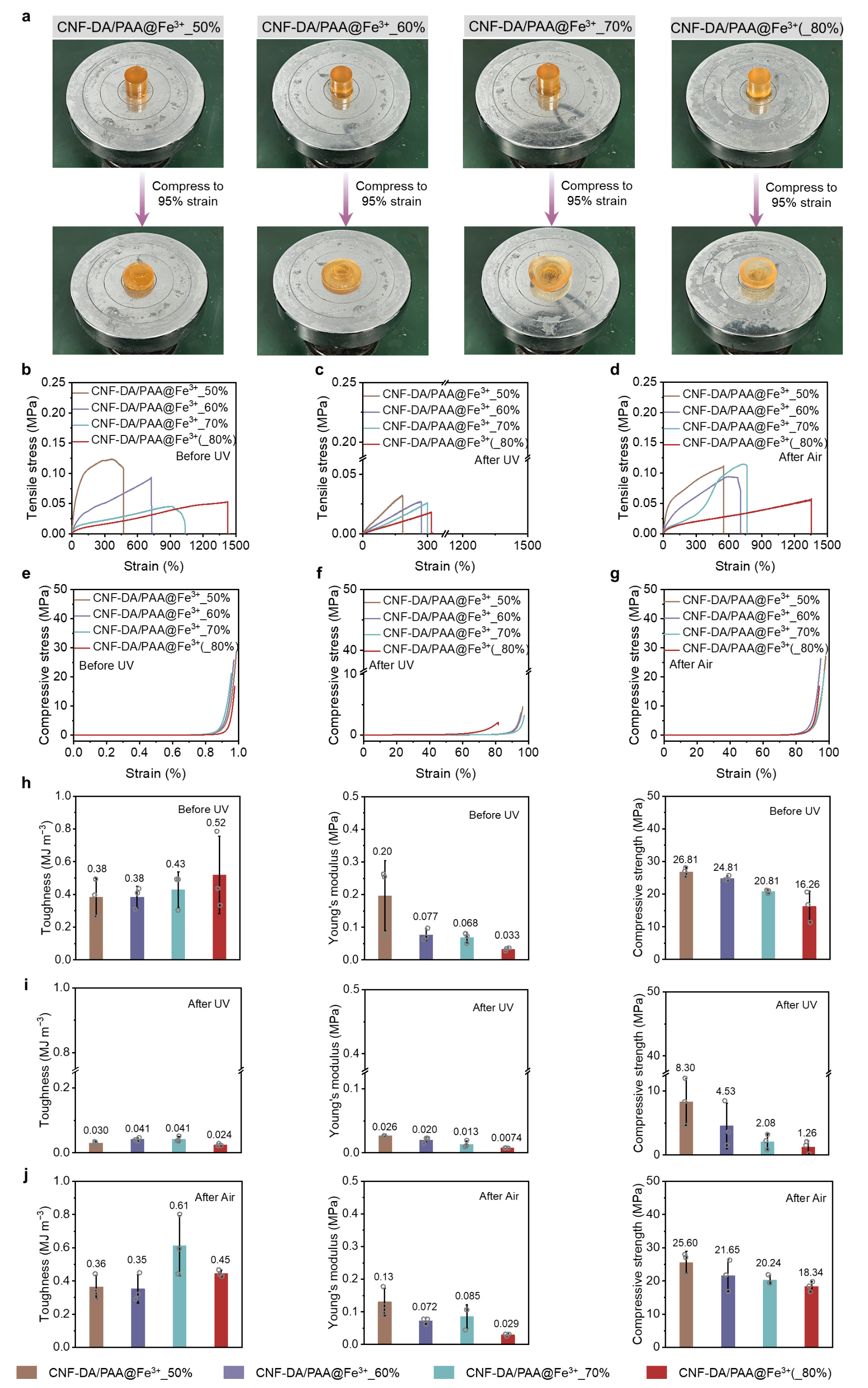


**Supplementary Fig. 20 | The mechanical properties of the CNF-DA/PAA@Fe^3+^ hydrogels prepared with different water contents. a** The digital images of the CNF-DA/PAA@Fe^3+^ hydrogels prepared with different water contents before and after compression. **b−d** Tensile stress-strain curves of the CNF-DA/PAA@Fe^3+^ hydrogels prepared with different water contents during the UV irradiation and air-oxidation process. **e−g** Compressive stress-strain curves of the CNF-DA/PAA@Fe^3+^ hydrogels prepared with different water contents during the UV irradiation and air-oxidation process. **h−j** Comparison of toughness, Young’s modulus, and compressive strength of the CNF-DA/PAA@Fe^3+^ hydrogels prepared with different water contents during the UV irradiation and air-oxidation process. Data in **h−j** are reported as their means ±SDs from *n* = 3 independent samples.

As shown in Supplementary Fig. 20a, the CNF-DA/PAA@Fe^3+^(_80%) hydrogel deforms greatly under external force, in sharp contrast, the CNF-DA/PAA@Fe^3+^_50% hydrogel maintains high integrity without distinct fracture and cracks under external force, indicating excellent mechanical strength and dimensional stability. The tensile and compressive stress-strain curves of various hydrogels before UV light are shown in Supplementary Fig. 20b, e. It can be observed that when the water content is gradually decreasing, both the ultimate tensile and compressive stress of the CNF-DA/PAA@Fe^3+^ hydrogel display an increasing trend. In particular, the CNF-DA/PAA@Fe^3+^_50% hydrogel exhibits the best tensile and properties, that is, the tensile Young’s modulus is 0.20 MPa corresponding to the toughness of 0.38 MJ m^−3^, 6.1 times higher than the CNF-DA/PAA@Fe^3+^(_80%) hydrogel (0.033 MPa); and the compressive ultimate strength is 26.81 MPa corresponding to the compressive fracture strain of 95%, 1.6 times higher than the CNF-DA/PAA@Fe^3+^(_80%) hydrogel (16.26 MPa) (Supplementary Fig. 20h). These jointly indicated that as a blocker, the water molecules inside the hydrogel are reduced, the coordination interaction among CNF-DA, Fe^3+^ ions, and PAA chains is enhanced, and the supramolecular network structure of the hydrogel is tighter.

With exposure to UV light, the mechanical performance of CNF-DA/PAA@Fe^3+^ hydrogel prepared with different water content is tunably transformed (Supplementary Fig. 20c, f). The CNF-DA/PAA@Fe^3+^_50% hydrogel displaced low tensile Young’s modulus of 0.026 MPa corresponding to the toughness of 0.030 MJ m^−3^, 3.5 times higher than the CNF-DA/PAA@Fe^3+^(_80%) hydrogel (0.0074 MPa); and the compressive ultimate strength is 8.30 MPa, 6.6 times higher than the CNF-DA/PAA@Fe^3+^(_80%) hydrogel (1.26 MPa), showing that UV light can regulate the stretchability and compressibility of the hydrogel (Supplementary Fig. 20i). Then when the hydrogel is oxidized in the air, the mechanical performance of the hydrogel recovers that before UV (Supplementary Fig. 20d, g and 20j).


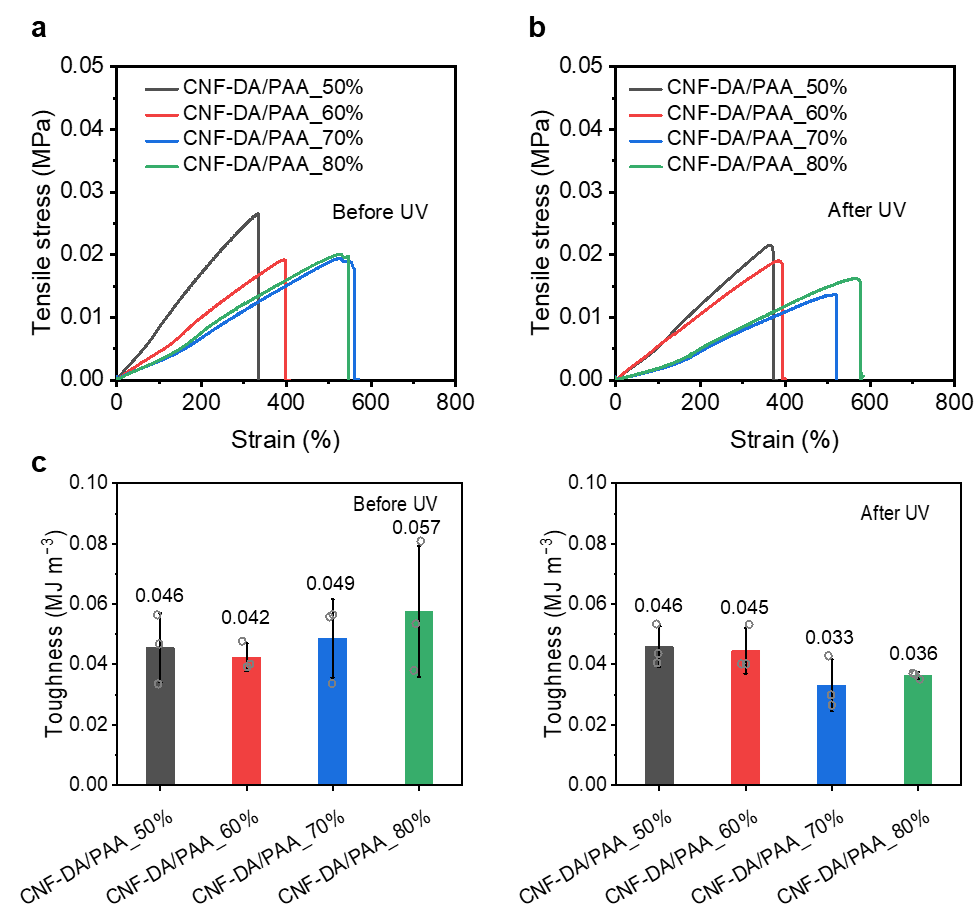


**Supplementary Fig. 21 | The mechanical properties of the CNF-DA/PAA hydrogels (without Fe ions) with different water contents.** **a,** **b** Tensile curves of the CNF-DA/PAA hydrogels with different water content (50%, 60%, 70%, 80%) before and after UV irradiation. **c** Comparison of the toughness of CNF-DA/PAA hydrogels with different water content (50%, 60%, 70%, 80%) before UV irradiation (n = 3). **d** Comparison of the toughness of CNF-DA/PAA hydrogels with different water content (50%, 60%, 70%, 80%) after UV irradiation. Data in **c**, **d** are reported as their means ±SDs from *n* = 3 independent samples.

We carried out the tensile tests of a series of CNF-DA/PAA hydrogels with different water contents to explain the role of Fe ions in enhancing the cross-linking of the polymer network. As shown in Supplementary Figure 21, the CNF-DA/PAA hydrogels with different water contents show weaker tensile toughness (0.046 MJ m^−3^ for 50%, 0.042 MJ m^−3^ for 60%, 0.049 MJ m^−3^ for 70%, 0.057 MJ m^−3^ for 80%) in comparison to those CNF-DA/PAA@Fe^3+^ hydrogels with different water contents, and the toughness of the CNF-DA/PAA hydrogels basically maintained a stable state during the UV irradiation process, where the Fe ions play a key role via forming strong yet photo-responsive coordination Fe^3+^-CNFs-DA supramolecular network.

**
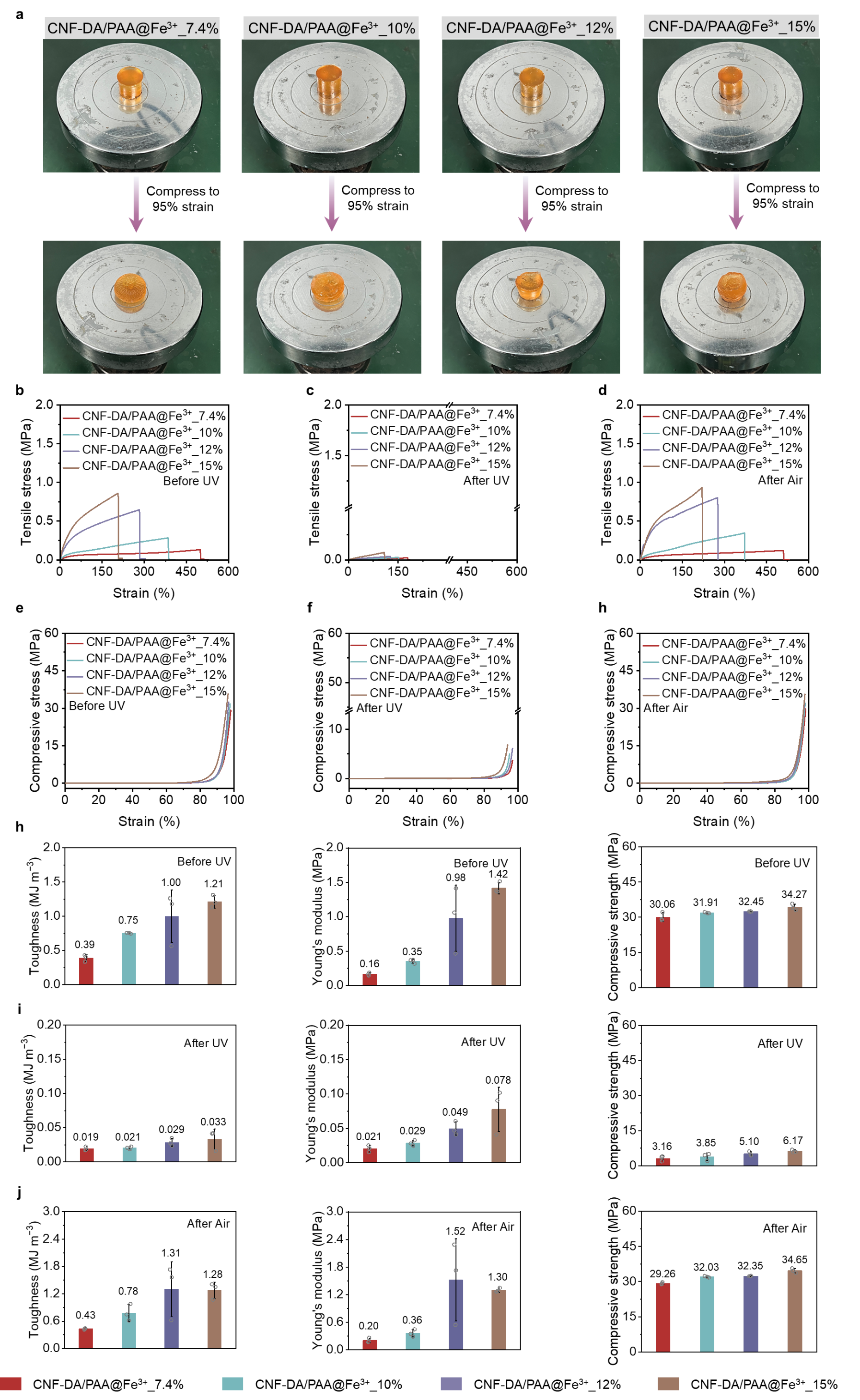
**

**Supplementary Fig. 22 | a** The digital images of the CNF-DA/PAA@Fe^3+^ hydrogels prepared with different CNF-DA content before and after compression (50% for water content). **b−d** Tensile stress-strain curves of the CNF-DA/PAA@Fe^3+^ hydrogels prepared with different CNF-DA content (50% for water content) during the UV irradiation and air-oxidation process. **e−g** Compressive stress-strain curves of the CNF-DA/PAA@Fe^3+^ hydrogels prepared with different CNF-DA content (50% for water content) during the UV irradiation and air-oxidation process. **h−j** Comparison of toughness, Young’s modulus, and compressive strength of the CNF-DA/PAA@Fe^3+^ hydrogels prepared with different CNF-DA content (50% for water content) during the UV irradiation and air-oxidation process. Data in **h−j** are reported as their means ±SDs from *n* = 3 independent samples.

As shown in Supplementary Fig. 22a, the CNF-DA/PAA@Fe^3+^_15% hydrogel maintains high integrity without distinct fracture and cracks under external force, indicating excellent mechanical strength and dimensional stability, in contrast, the CNF-DA/PAA@Fe^3+^_7.4% hydrogel deforms slightly under external force (50% water content for all the hydrogels). The tensile and compressive stress-strain curves of various hydrogels during the UV light irradiation and air-oxidation process are shown in Supplementary Fig. 22b−g. When the CNF-DA content is gradually increasing, both the ultimate tensile and compressive stress of the CNF-DA/PAA@Fe^3+^ hydrogel display an increasing trend. In particular, the CNF-DA/PAA@Fe^3+^_15% hydrogel exhibits the best tensile and compressive properties, that is, the toughness of 1.21 MJ m^−3^ corresponding to the tensile Young’s modulus is 1.42 MPa, 3.1 times higher than the CNF-DA/PAA@Fe^3+^_7.4% hydrogel (0.39 MJ m^−3^); and the compressive ultimate strength is 34.27 MPa corresponding to the compressive fracture strain of 95%, 1.1 times higher than the CNF-DA/PAA@Fe^3+^_7.4% hydrogel (30.06 MPa). With exposure to the UV light and further air-oxidation process, the strong yet reversible stretchability and compression of the CNF-DA/PAA@Fe^3+^_15% hydrogel recovers that before UV irradiation (Supplementary Fig. 22h−j). More interestingly, by adjusting the water content of the hydrogel or adjusting the cellulose content by maintaining the water content, the mechanical properties of the hydrogel have made a qualitative leap.


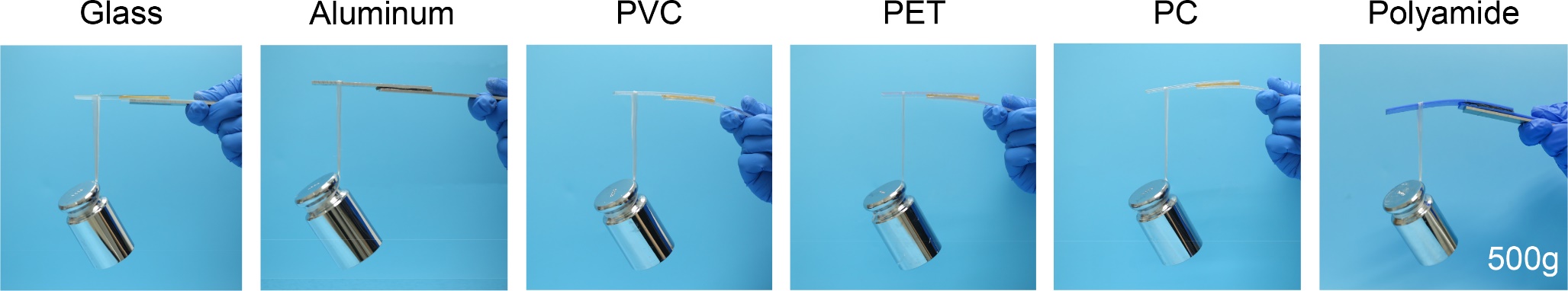


**Supplementary Fig. 23 | Photographs of the same two pieces of various representative substrates adhered by the CNF-DA/PAA@Fe^3+^ hydrogel to support a weight of 500 g.** The developed hydrogel can also tightly adhere to the same two pieces of representative substrates and easily supports a 500 g weight hanging from the end of the substrate, revealing its capacity for strong and stable adhesion.


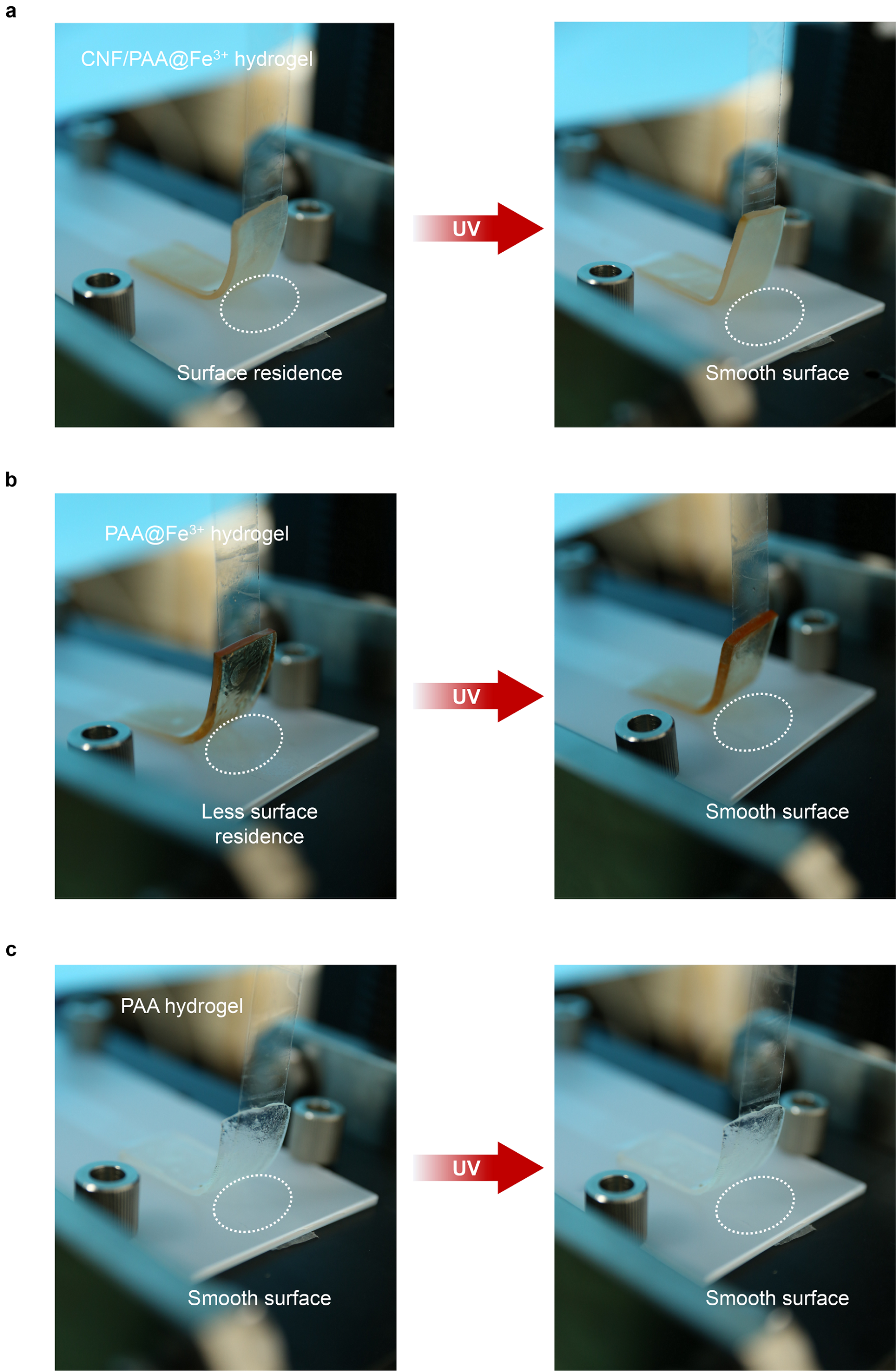


**Supplementary Fig. 24 | The standard 90-degree peel test of the CNF/PAA@Fe^3+^ hydrogel, PAA@Fe^3+^ hydrogel, and PAA hydrogel.**


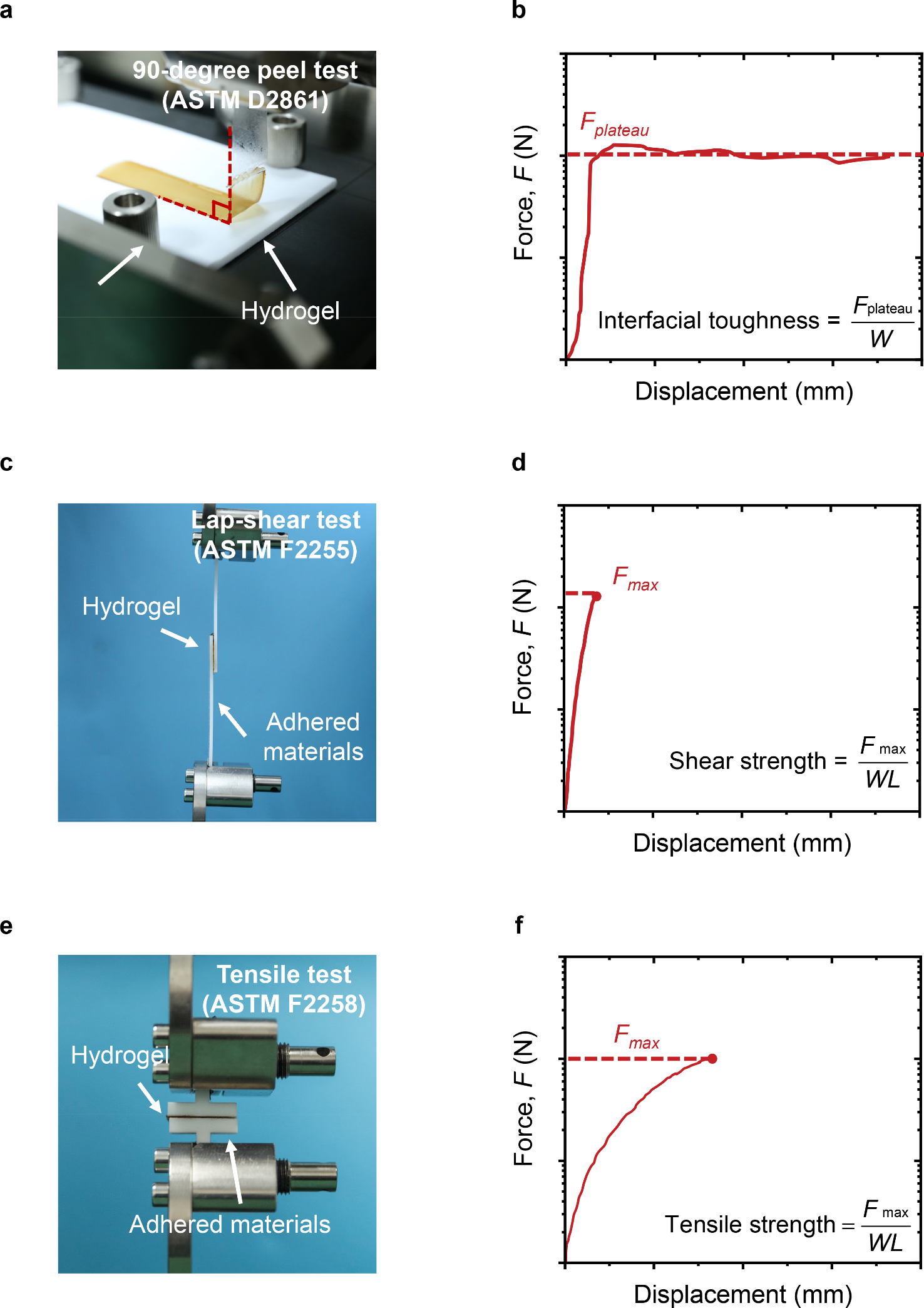


**Supplementary Fig. 25 | Setups for mechanical testing of adhesion performance of the CNF-DA/PAA@Fe^3+^ hydrogel. a**, **b** Photograph and setup for measurement of interfacial toughness based on the standard 90-degree peel test (ASTM D2861). **c**, **d** Photograph and setup for measurement of shear strength based on the standard lap-shear test (ASTM F2255). **e**, **f** Photograph and setup for measurement of tensile strength based on the standard tensile test (ASTM F2258). *F* force; *F*_plateau_, plateau force in a peeling test; *F*_max_, maximum force in the lap-shear and tensile test; *L*, length; *W,* width.


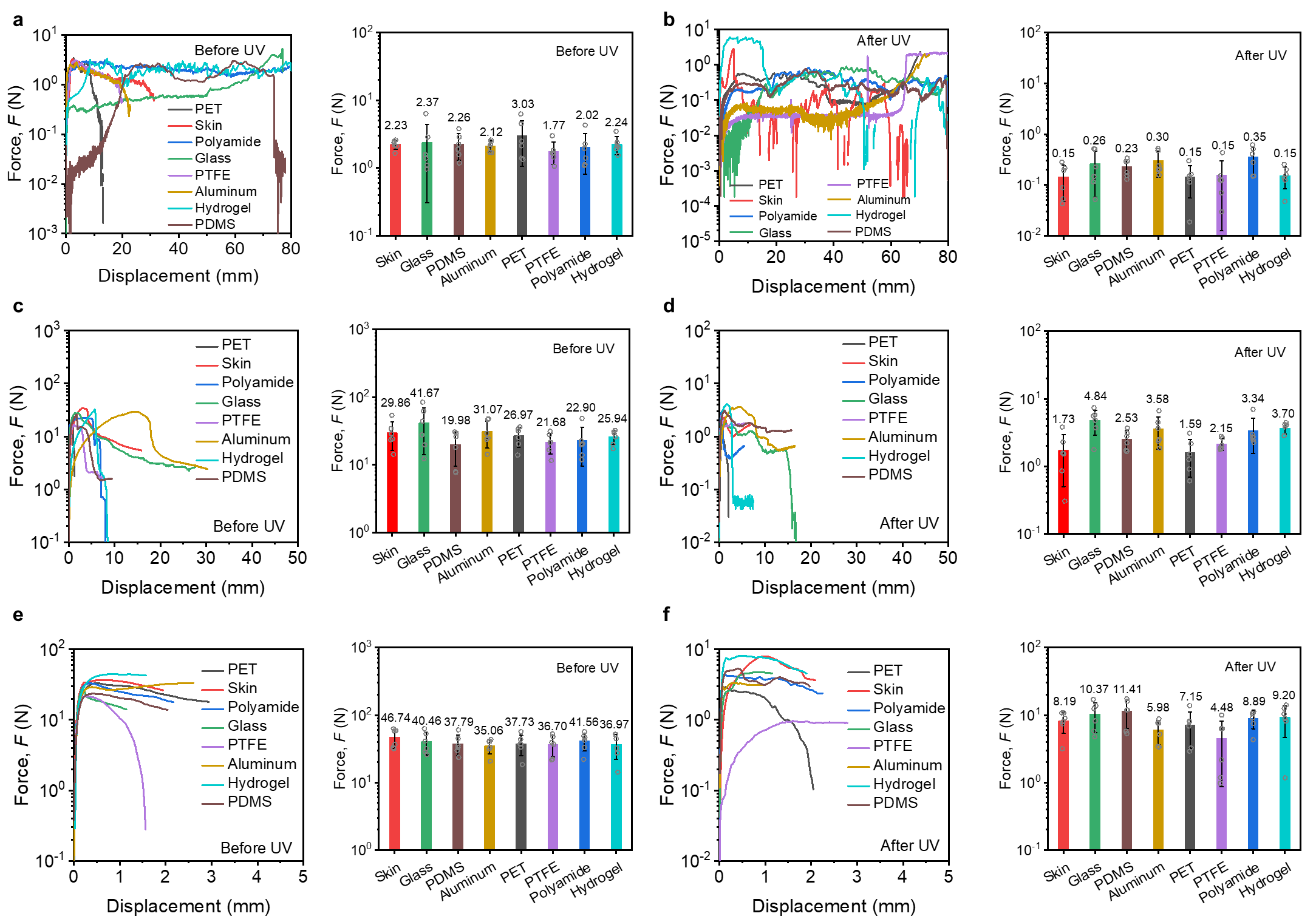


**Supplementary Fig. 26 | Comparison of adhesion force of the CNF-DA/PAA@Fe^3+^ hydrogel to different substrates. a**, **b** 90-degree peeling test curve of the CNF-DA/PAA@Fe^3+^ hydrogel before and after UV irradiation. **c**, **d** Lap-shear test curve of the CNF-DA/PAA@Fe^3+^ hydrogel before and after UV irradiation. **e**, **f** Tensile test curve of the photo-detachable hydrogels before and after UV irradiation. Data in **a−f** are reported as their means ±SDs from *n* = 6 independent samples.

**
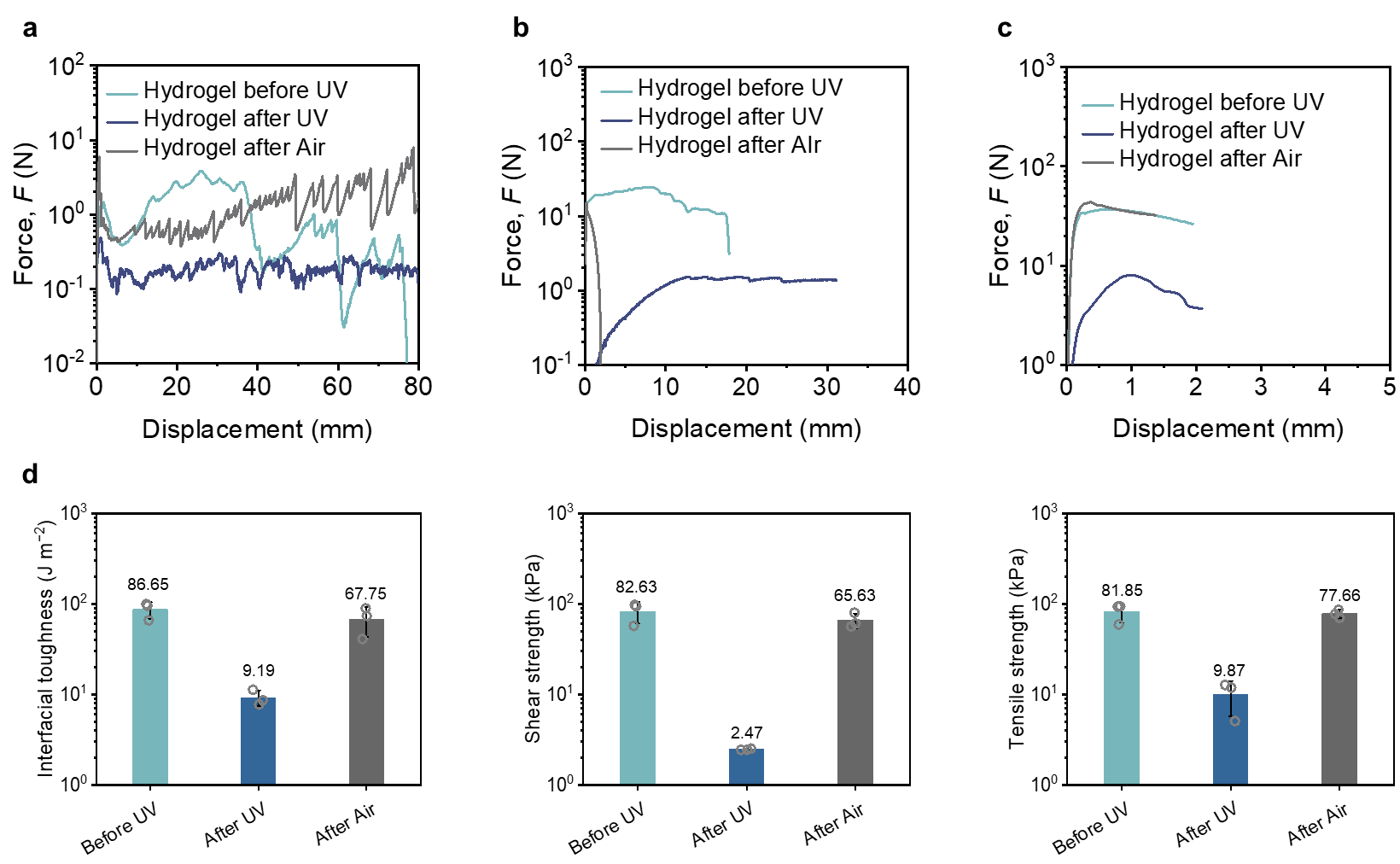
**

**Supplementary Fig. 27 | Comparison of adhesion force of the CNF-DA/PAA@Fe^3+^ hydrogel during the UV irradiation and air-oxidation process on the freshly excised porcine skin. a−c** The peel, shear, and tensile force-displacement curves of the CNF-DA/PAA@Fe^3+^ hydrogels on the substrate of freshly excised porcine skin during the UV irradiation and air-oxidation process. **d** Comparison of the interfacial toughness, shear strength, and tensile strength of the CNF-DA/PAA@Fe^3+^ hydrogels on the substrate of freshly excised porcine skin during the UV irradiation and air-oxidation process. Data in **d** are reported as their means ±SDs from *n* = 3 independent samples.

**
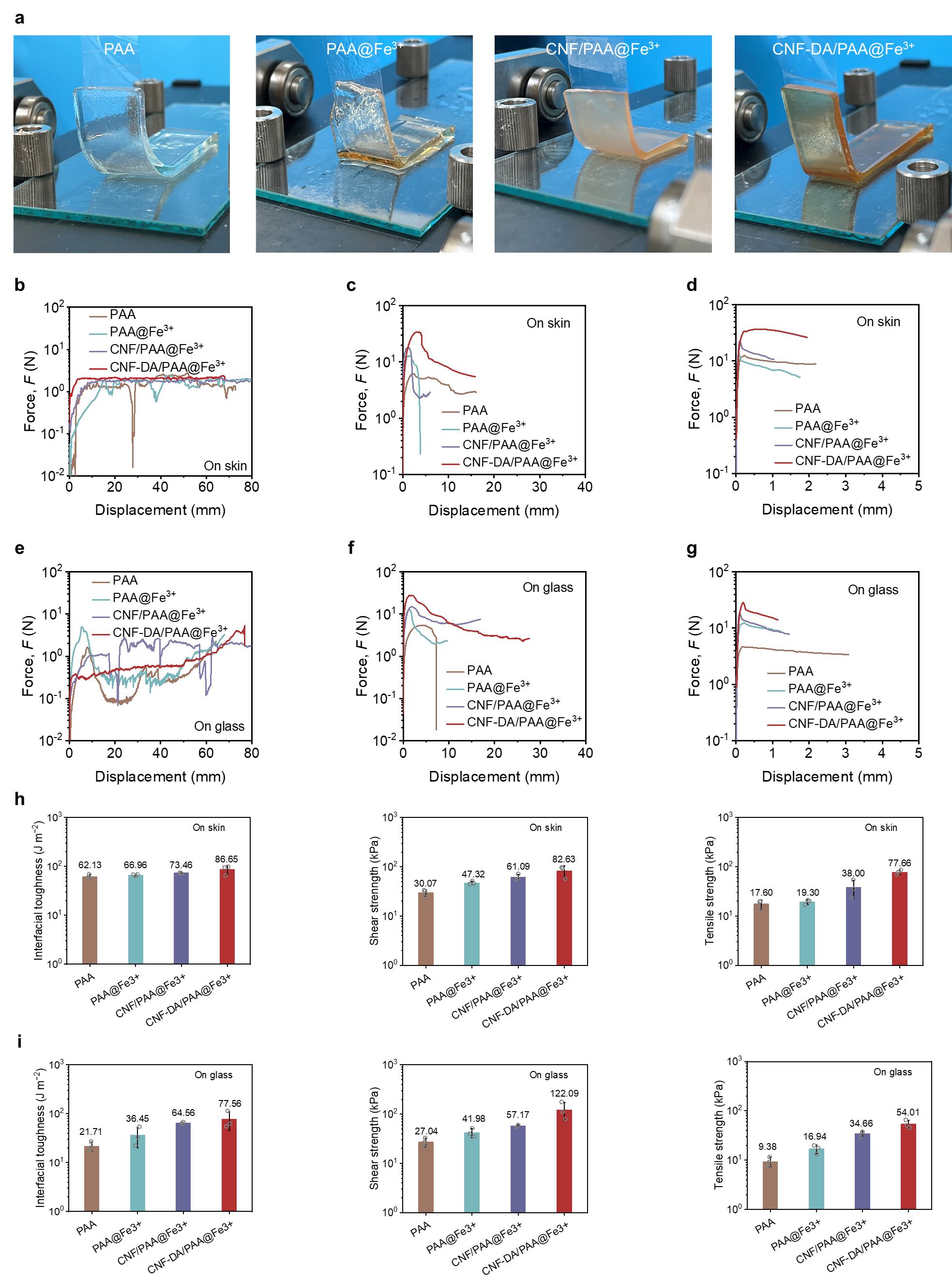
**

**Supplementary Fig. 28 | Comparison of the adhesion energy and strain of different hydrogels.** **a** The peeling process digital images of the PAA, PAA@Fe^3+^, CNF/PAA@Fe^3+^, and CNF-DA/PAA@Fe^3+^ hydrogels on the substrate of engineering glass. **b−d** 90-degree peel, lap-shear, and tensile force-displacement curves of the PAA, PAA@Fe^3+^, CNF/PAA@Fe^3+^, and CNF-DA/PAA@Fe^3+^ hydrogels on the substrate of freshly excised porcine skin. **e−g** 90-degree peel, lap-shear, and tensile force-displacement curves of the PAA, PAA@Fe^3+^, CNF/PAA@Fe^3+^, and CNF-DA/PAA@Fe^3+^ hydrogels on the substrate of engineering glass. **h,** **i** Comparison of the interfacial toughness, shear strength, and tensile strength of the PAA, PAA@Fe^3+^, CNF/PAA@Fe^3+^, and CNF-DA/PAA@Fe^3+^ hydrogels on the substrate of freshly excised porcine skin and engineering glass. Data in **h**, **i** are reported as their means ±SDs from *n* = 3 independent samples.


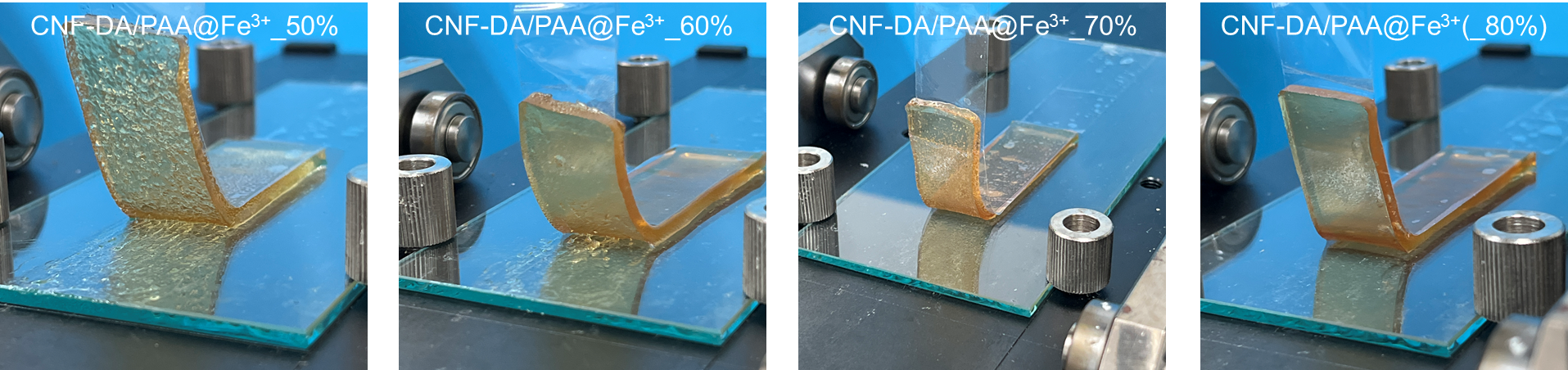


**Supplementary Fig. 29 | The digital images of the CNF-DA/PAA@Fe^3+^ hydrogels with different water contents.**

As shown in Supplementary Fig. 29, the CNF-DA/PAA@Fe^3+^(_80%) hydrogel, which can be easily removed from the engineering glass, shows good adhesive strength. In particular, the CNF-DA/PAA@Fe^3+^_50% hydrogel exhibits a pull on the glass observed without visible fracture when detached from the glass, and the hydrogel surface appeared with the obvious bristle-like patterns, indicating an enhanced adhesive strength and mechanical toughness because of the reduction of the surface hydration layer of the hydrogel, exposing more interfacial adhesive groups, and increasing the number of adhesion sites on the interface with the object to adhere, as well as improved interfacial interaction.


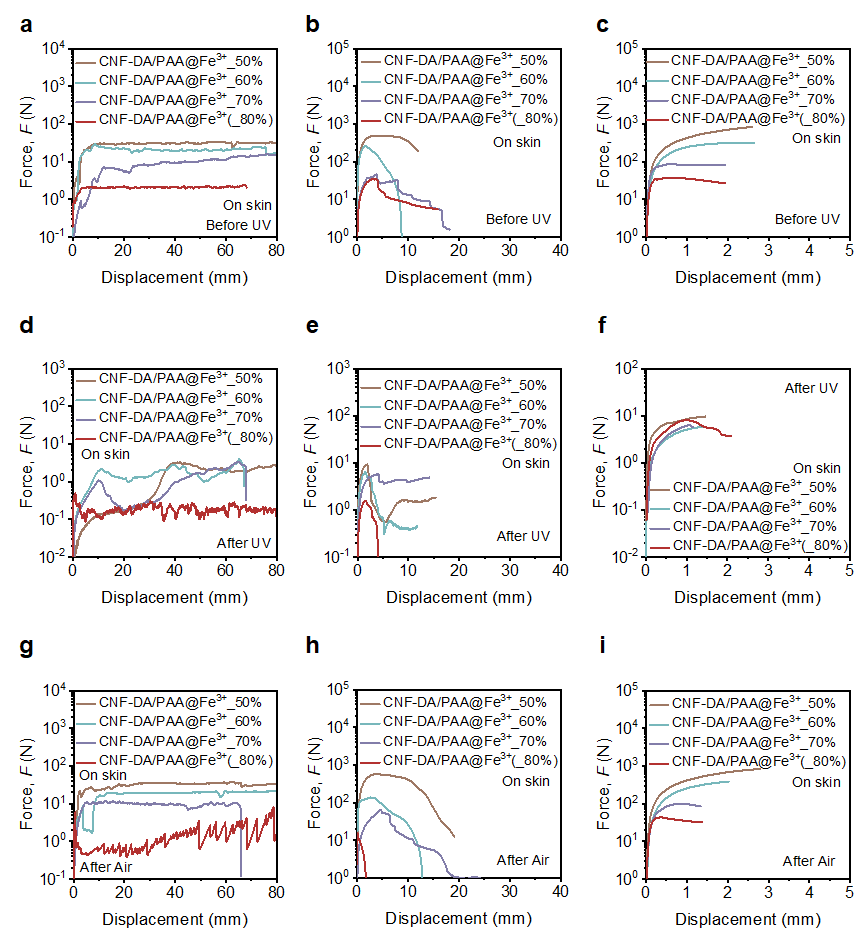


**Supplementary Fig. 30 | The adhesive force-displacement curves of the CNF-DA/PAA@Fe^3+^ hydrogels prepared with different water content on the freshly excised porcine skin.** **a−c** 90-degree peel, lap-shear, and tensile force-displacement curves of the CNF-DA/PAA@Fe^3+^ hydrogels prepared with different water content on the substrate of freshly excised porcine skin before UV irradiation. **d−f** 90-degree peel, lap-shear, and tensile force-displacement curves of the CNF-DA/PAA@Fe^3+^ hydrogels prepared with different water content on the substrate of freshly excised porcine skin after UV irradiation. **g−i** 90-degree peel, lap-shear, and tensile force-displacement curves of the CNF-DA/PAA@Fe^3+^ hydrogels prepared with different water content on the substrate of freshly excised porcine skin after air oxidation.


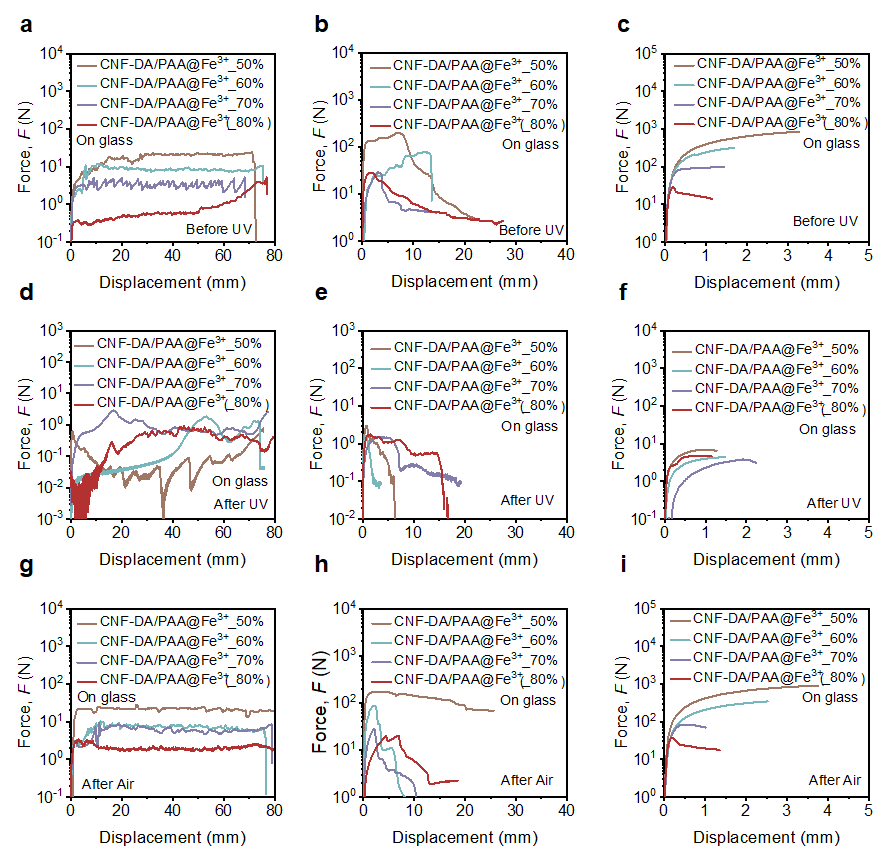


**Supplementary Fig. 31 | The adhesive force-displacement curves of the CNF-DA/PAA@Fe^3+^ hydrogels prepared with different water contents on the engineering glass.** **a−c** 90-degree peel, lap-shear, and tensile force-displacement curves of the CNF-DA/PAA@Fe^3+^ hydrogels prepared with different water content on the substrate of engineering glass before UV irradiation. **d−f** 90-degree peel, lap-shear, and tensile force-displacement curves of the CNF-DA/PAA@Fe^3+^ hydrogels prepared with different water content on the substrate of engineering skin after UV irradiation. **g−i** 90-degree peel, lap-shear, and tensile force-displacement curves of the CNF-DA/PAA@Fe^3+^ hydrogels prepared with different water content on the substrate of engineering glass after air oxidation.

We quantitatively evaluated the reversible adhesive properties of prepared hydrogels with different water content by 90-degree peeling, lap-shear, and tensile tests on the substrates of freshly excised porcine skin and engineering glass during the in-situ UV irradiation and air oxidation process (Supplementary Figs. 30 and 31). The peel force-displacement, shear force-displacement, and tensile force-displacement curves of hydrogels on the skin before UV irradiation are shown in Supplementary Fig. 30a−c, and the plateau force, shear force, and tensile force of CNF-DA/PAA@Fe^3+^_80% hydrogel are around 2.23 N, 29.86 N, and 46.74 N on the skin (2.37 N for plateau force, 41.67 N for shear force, and 40.46 N for tensile force on the glass) (Supplementary Fig. 28a−c), showing inherent weak adhesion strength. With reducing the water content of the hydrogel, the platform force, shear force, and tensile force of the CNF-DA/PAA@Fe^3+^ hydrogel display an upward trend, among which, the CNF-DA/PAA@Fe^3+^_50% hydrogel exhibits the highest platform force, shear force, and tensile force before UV irradiation (30.46 N for plateau force, 338.16 N for shear force, and 778.15 N for tensile force on the skin; 17.38 N for plateau force, 173.28 N for shear force, and 812.01 N for tensile force on the glass).

In addition, it is worth mentioning that after UV irradiation, the plateau force, shear force, and tensile force of CNF-DA/PAA@Fe^3+^_80% hydrogel are around 0.15 N, 8.19 N and 1.73 N on the skin (0.26 N for plateau force, 10.37 N for shear force, and 4.84 N for tensile force on the glass) (Supplementary Fig. 30d−f and Supplementary Fig. 31d−f), which are 14.9 times for plateau force, 3.6 times for shear force, and 27.0 times for tensile force of the CNF-DA/PAA@Fe^3+^_80% hydrogel on the skin before UV irradiation (9.1 times for plateau force, 4.0 times for shear force, and 8.4 times for tensile force on the glass). In particular, with the reduction of water content, the platform force, shear force, and tensile force of the CNF-DA/PAA@Fe^3+^ hydrogel display a downward trend, among which, the CNF-DA/PAA@Fe^3+^_50% hydrogel exhibits the highest tunable adhesive force ratios after UV irradiation (comparing before UV, 14.0 times for plateau force, 56.0 times for shear force, and 128.0 times for tensile force on the skin; 61.2 times for plateau force, 85.4 times for shear force, and 133.8 times N for tensile force on the glass) (Supplementary Fig. 30d−f and Supplementary Fig. 31d−f). These 90-degree peeling, lap-shear, and tensile tests results after air oxidation are consistent with the peeling, shear, and tensile behavior of hydrogels before UV irradiation from the skin and glass shown in Supplementary Fig. 30g−i and Supplementary Fig. 31g−i.


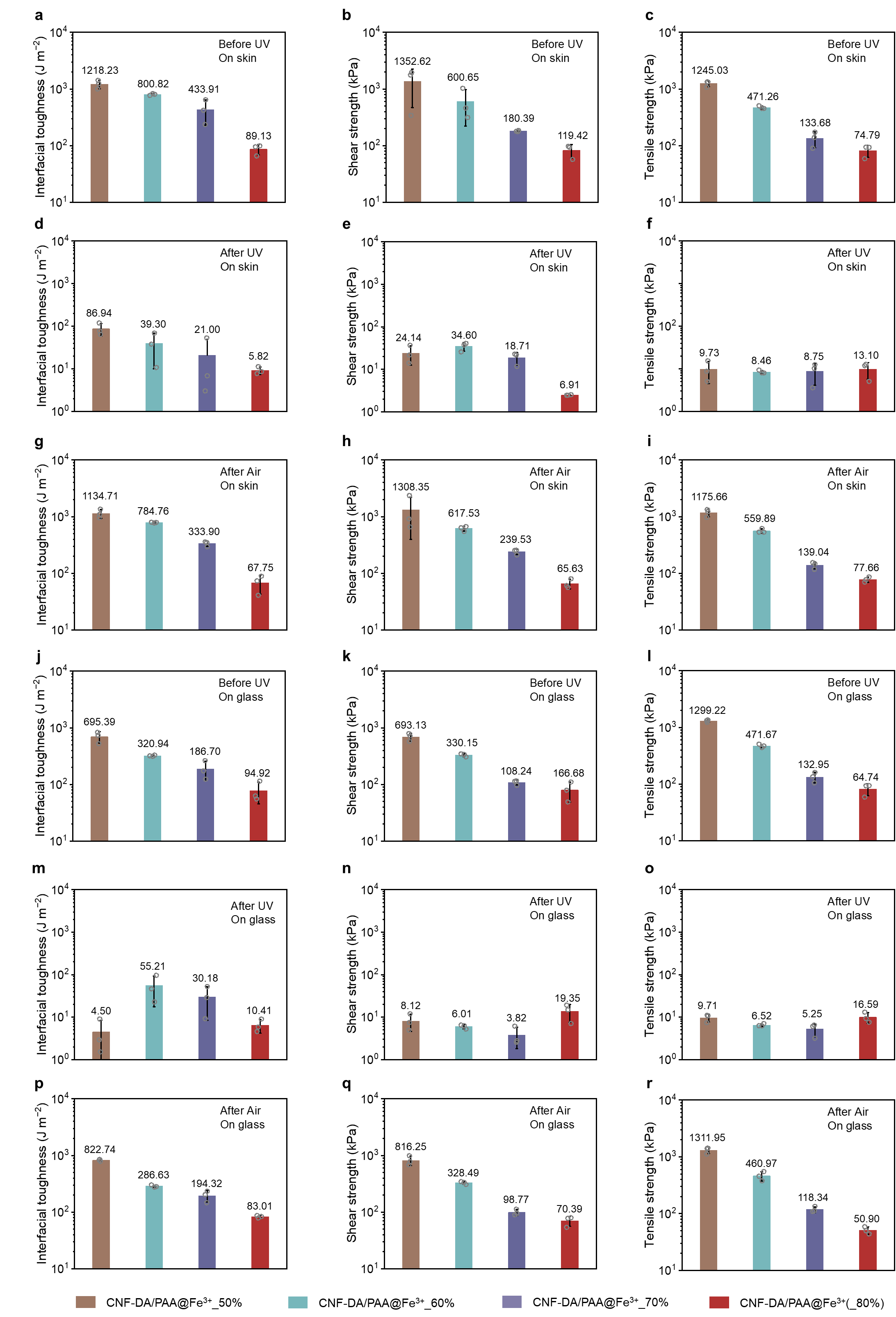


**Supplementary Fig. 32 | Comparison of the adhesive performance of the CNF-DA/PAA@Fe^3+^ hydrogels with different water contents on the skin and glass. a−c** Comparison of interfacial toughness, shear strength, and tensile strength of the CNF-DA/PAA@Fe^3+^ hydrogels prepared with different water contents on the substrates of skin before UV irradiation. **d−f** Comparison of interfacial toughness, shear strength, and tensile strength of the CNF-DA/PAA@Fe^3+^ hydrogels prepared with different water content on the substrates of glass before UV irradiation. **g−i** Comparison of interfacial toughness, shear strength, and tensile strength of the CNF-DA/PAA@Fe^3+^ hydrogels with different water contents on the substrates of skin after UV irradiation. **j−l** Comparison of interfacial toughness, shear strength, and tensile strength of the CNF-DA/PAA@Fe^3+^ hydrogels prepared with different water contents on the substrates of glass after UV irradiation. **m−o** Comparison of interfacial toughness, shear strength, and tensile strength of the CNF-DA/PAA@Fe^3+^ hydrogels prepared with different water contents on the substrates of skin after air. **p−r** Comparison of interfacial toughness, shear strength, and tensile strength of the CNF-DA/PAA@Fe^3+^ hydrogels prepared with different water contents on the substrates of glass after air. Data in **a−r** are reported as their means ±SDs from *n* = 3 independent samples.

To objectively estimate the reversible adhesive performance of the CNF-DA/PAA@Fe^3+^ hydrogels with various water contents, we compared the interfacial toughness, shear strength, and tensile strength during the UV irradiation and air oxidation process. The interfacial toughness, shear strength, and tensile strength between CNF-DA/PAA@Fe^3+^_50% and CNF-DA/PAA@Fe^3+^_80% hydrogel before UV light on the substrate of freshly excised porcine skin increase 13.7-fold (1218.23 J m^−2^ vs. 89.13 J m^−2^), 11.3-fold (1352.62 kPa vs 119.42 kPa), and 16.6-fold (1245.03 kPa vs 74.79 kPa) (7.3-fold for interfacial toughness, 4.2-fold for shear strength, and 20.1-fold for tensile strength on the engineering glass), respectively. With exposure to UV light, the interfacial toughness, shear strength, and tensile strength gradually decrease, and the tunable interfacial toughness, shear strength, and tensile strength ratios of CNF-DA/PAA@Fe^3+^_50% hydrogel are 92.9%, 98.2% and 99.2%, which is higher than the tunable interfacial toughness, shear strength, and tensile strength ratios of CNF-DA/PAA@Fe^3+^_80% hydrogel (93.4%, 94.2%, 82.5%), then when the hydrogel is oxidized in the air, the adhesive performance of the hydrogel recovers that before UV. More attractively, our obtained hydrogel displayed a high adhesion strength, which is better to most reported switchable adhesive hydrogels.


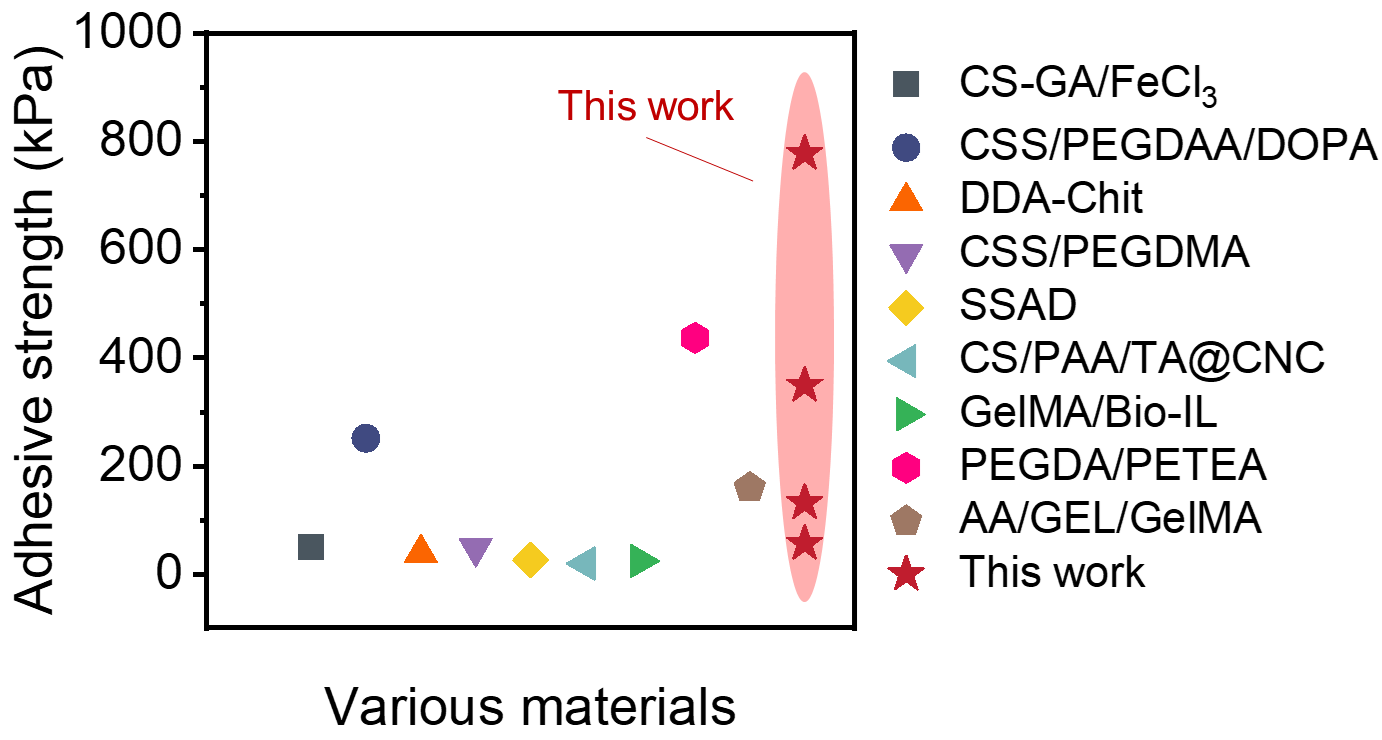


**Supplementary Fig. 33 | Comparison of the adhesive performance of the CNF-DA/PAA@Fe^3+^ hydrogels with different water contents on the glass and other reported works.** Comparison of the adhesive strength with other adhesive materials and CNF-DA/PAA@Fe^3+^ hydrogel with different water contents.^10−12^

**
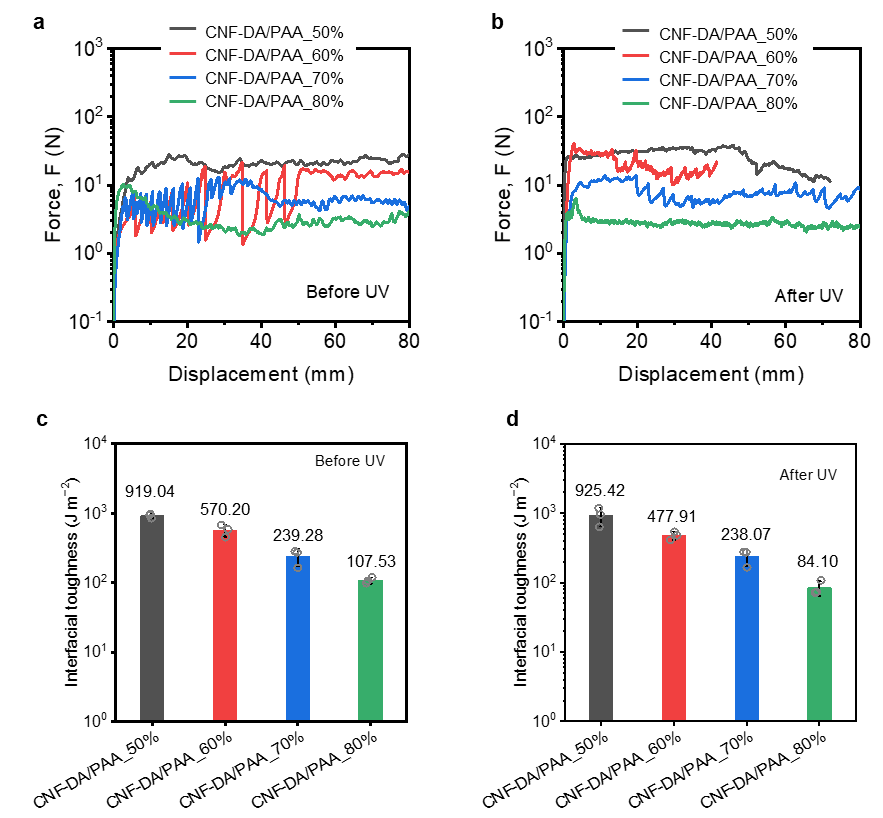
**

**Supplementary Fig. 34 | The adhesive force-displacement curves of the CNF-DA/PAA hydrogels (without Fe ions) with different water contents on the engineering glass. a,** **b** 90-peeling curves of the CNF-DA/PAA hydrogels with different water content (50%, 60%, 70%, 80%) before and after UV irradiation on the substrate of glass. **c, d** Comparison of the interfacial toughness of the CNF-DA/PAA hydrogels with different water content (50%, 60%, 70%, 80%) before and after UV irradiation. Data in **c**, **d** are reported as their means ±SDs from *n* = 3 independent samples.

We explored the interfacial toughness of a series of CNF-DA/PAA hydrogels without Fe ions with different water contents on the glass before and after UV irradiation. The interfacial toughness of all CNF-DA/PAA hydrogels basically maintains a stable state (919.04 J m^−2^ for 50%, 570.20 J m^−2^ for 60%, 239.28 J m^−2^ for 70%, 107.53 J m^−2^ for 80%) during the UV irradiation process. These results demonstrated that Fe ions as part of the internal network of the hydrogel participated in the formation of the network structure, and cooperated with CNF fibers and PAA chains to enhance the cross-linking density of the hydrogel. Simultaneously, upon the UV irradiation, the transformation of Fe^3+^ ions to Fe^2+^ ions in the photo-Fenton-like reaction (the CNF fibers serves as a Fenton-like reagent) led to the dissociation and reconstruction of Fe ions and CNF networks, achieving the regulatory of the cohesive strength of the hydrogels, thereby affecting the interfacial bonding interactions.


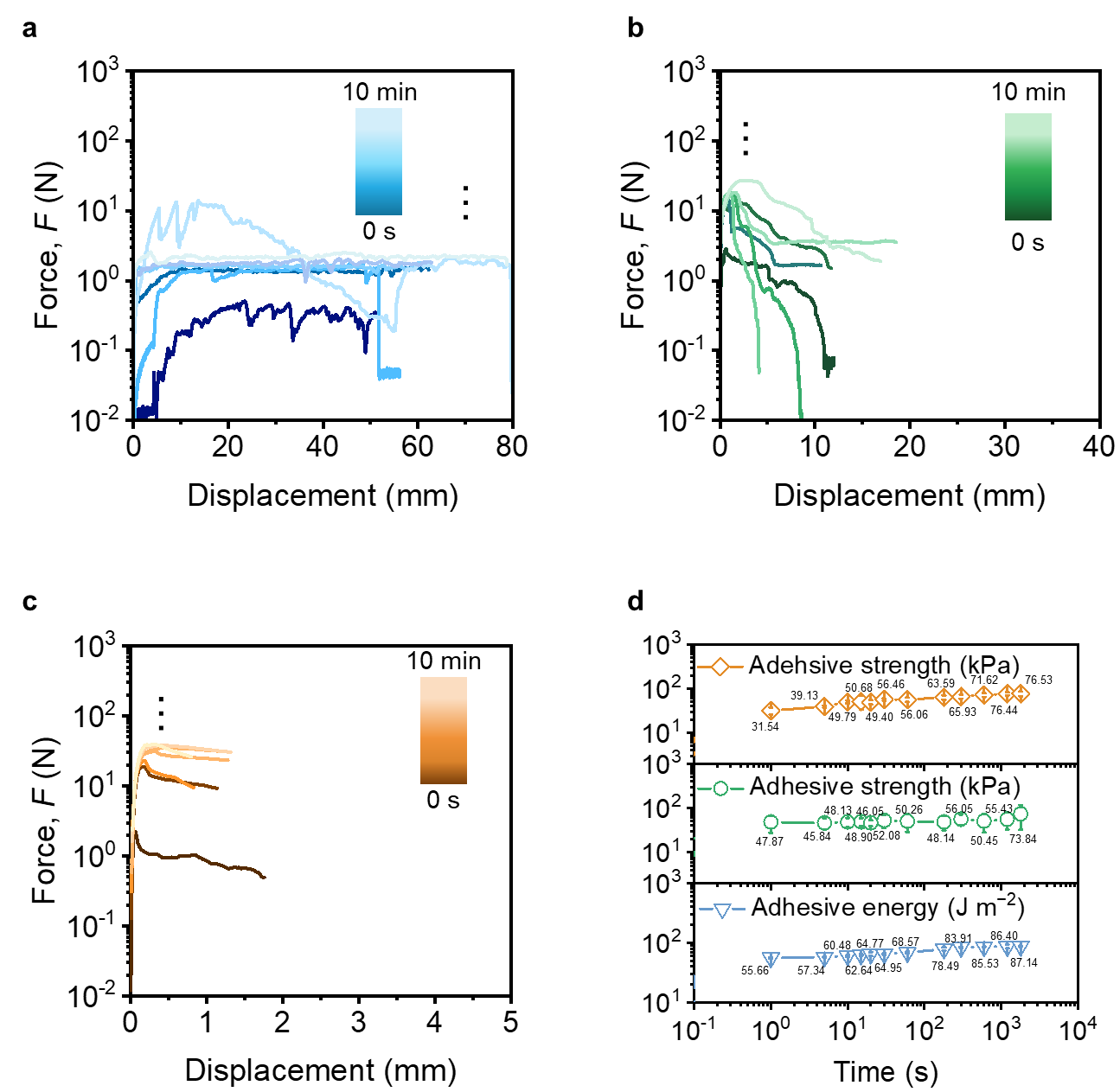


**Supplementary Fig. 35 | The adhesion strength of the CNF-DA/PAA@Fe^3+^ hydrogel to the freshly excised porcine skin after air-oxidation. a-c** 90-degree peel, lap-shear, and tensile force-displacement curves of the CNF-DA/PAA@Fe^3+^ hydrogels with the extension of oxidation time on the skin. **d** Comparison of adhesive energy and adhesive strength of the CNF-DA/PAA@Fe^3+^ hydrogels with the extension of oxidation time on the skin. Data in **d** are reported as their means ±SDs from *n* = 3 independent samples.

To determine the recovery time of the adhesive strength of the CNF-DA/PAA@Fe^3+^ hydrogel with a water content of 80% during the air-oxidation process, we conducted the 90-degree peel, lap-shear, and tensile tests with different oxidation times on the substrate of the freshly excised porcine skin. The peel, shear, and force-displacement curves of CNF-DA/PAA@Fe^3+^ hydrogel are shown in Supplementary Fig. 35a−c, and the plateau, shear, and tensile force of CNF-DA/PAA@Fe^3+^ hydrogel as the oxidation time reached 5 minutes are around 2.10 N, 14.01 N, 41.21 N, respectively (2.23 N, 29.86 N, and 46.73 N before UV irradiation), exhibiting fast and excellent recovery capability of the adhesive strength. Meanwhile, we explored the adhesion energy and adhesive strength of the CNF-DA/PAA@Fe^3+^ hydrogels within 1 hour. It can be seen that the adhesion strength and adhesion energy of the hydrogels have recovered that before UV irradiation with the exposure to the air-oxidation for 5 minutes, and remained unchanged after 5 minutes, which demonstrates that the CNF-DA/PAA@Fe^3+^ hydrogel exhibits the flexibly tunable adhesion properties.


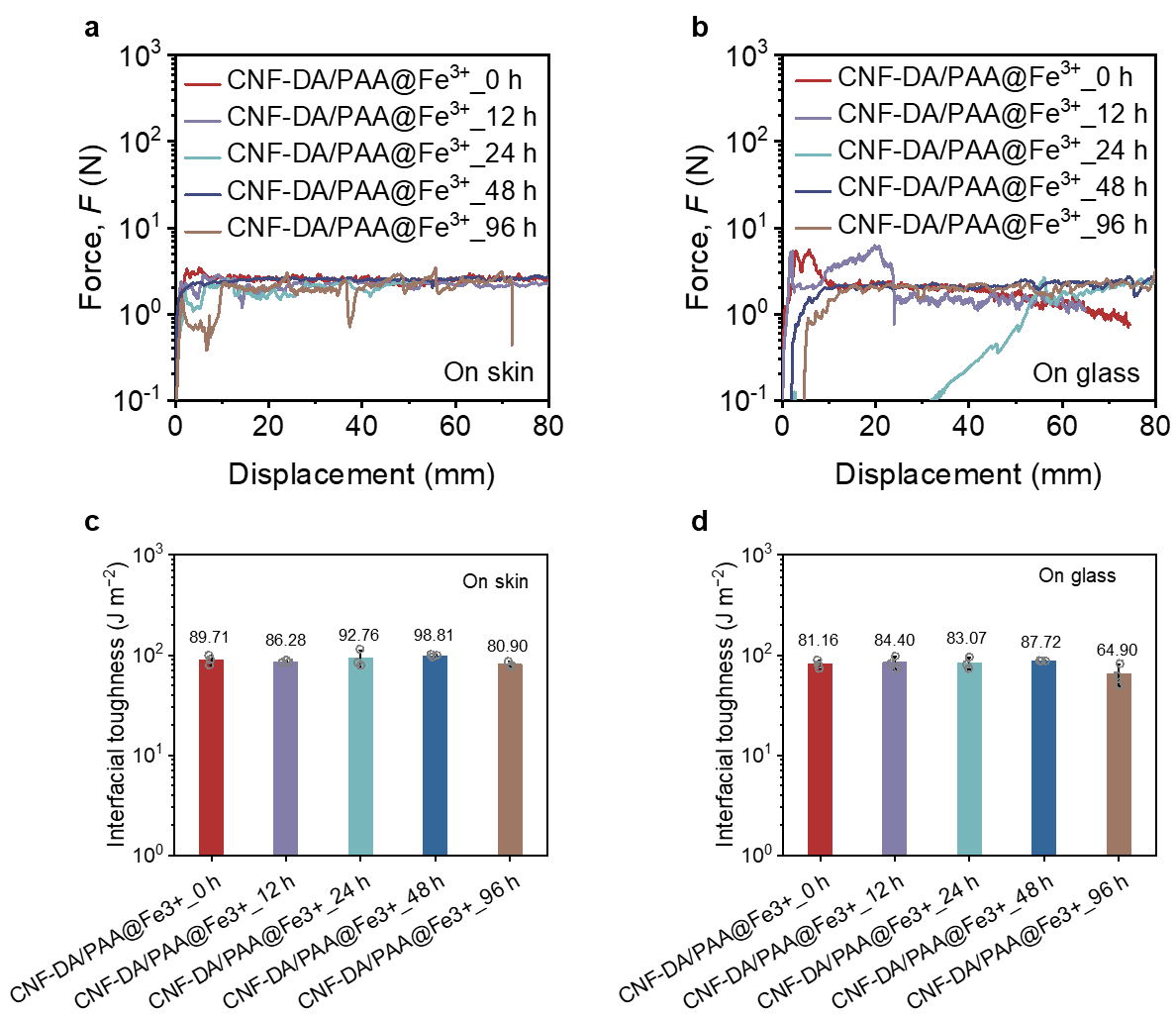


**Supplementary Fig. 36 | Stability of the adhesive properties of the hydrogels.** **a,** **b** 90-degree peel force-displacement curves of the CNF-DA/PAA@Fe^3+^ hydrogels with different times on the substrates of freshly excised porcine skin and engineering glass, respectively. **c, d** Comparison of the interfacial toughness of CNF-DA/PAA@Fe^3+^ hydrogels with different times on the substrates of freshly excised porcine skin and engineering glass, respectively. Data in **c**, **d** are reported as their means ±SDs from *n* = 3 independent samples.

The adhesion properties of these hydrogels with varying oxidation times were further investigated by 90-degree peeling tests on skin and glass (Supplementary Fig. 36a and 36b). For working on skin and glass, both the hydrogels showed similar peel force-displacement curves, that is, the maximum peel force of the hydrogels all displayed a slightly decreasing trend with increasing oxidation time. Typically, compared to CNF-DA/PAA@Fe^3+^_0h hydrogel, the interfacial toughness of CNF-DA/PAA@Fe^3+^_96h hydrogel applied on skin and glass decreased by 1.1 times (from 89.71 J m^−2^ to 80.90 J m^−2^) and 1.3 times (from 81.16 J m^−2^ to 64.90 J m^−2^), showing a considerable attenuation, which is attributed to that the catechol groups in the hydrogel are partially oxidized leading to weaken interfacial interactions with substrates (Supplementary Fig. 36c and 36d). In summary, under the action of O_2_, the catechol groups in the hydrogel were partially oxidized, leading to an increase in the crosslinking density, the hydrogel still maintains excellent toughness and adhesive strength, indicating that the hydrogel has considerable stability.


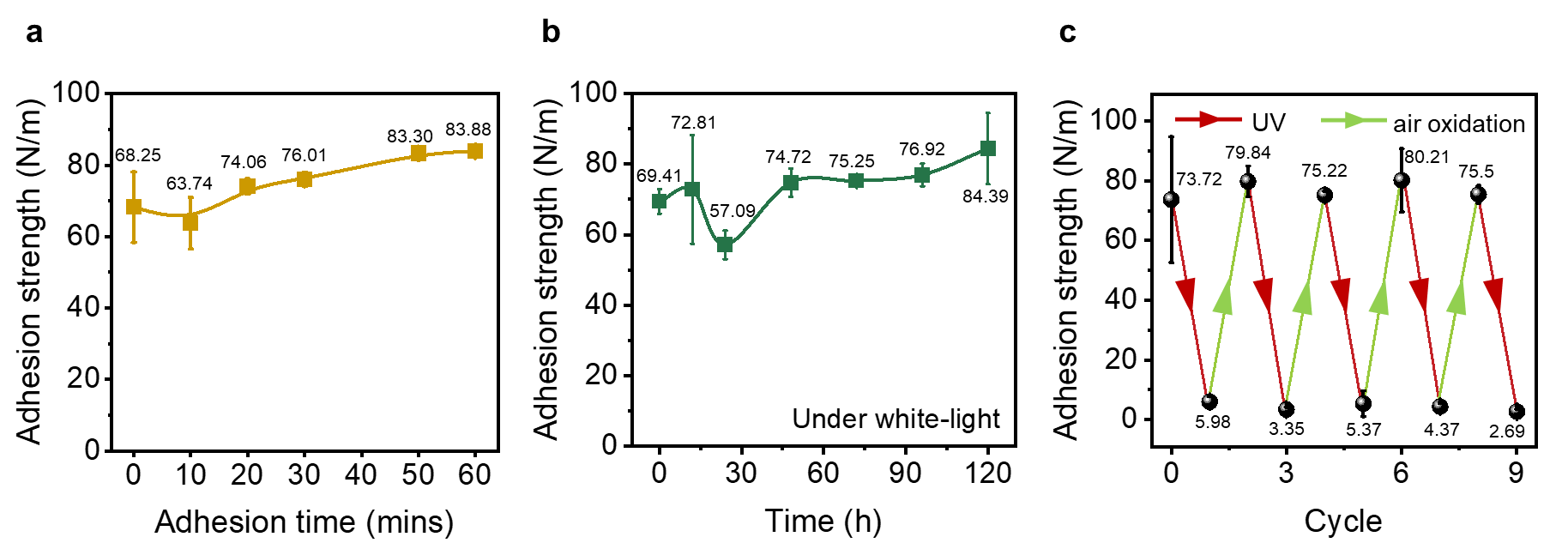


**Supplementary Fig. 37 | The adhesion strength of the CNF-DA/PAA@Fe^3+^ hydrogel to the engineering glass. a** The adhesion strength of the hydrogel maintains stability as the adhesion time increases. **b** Adhesion strength remains stable for a long period after being exposed to white light. **c** The stability and reusability of the hydrogel under periodical change of the UV irradiation and air oxidation elucidates the reusability of the photo-detachable hydrogel. Data in **a−c** are reported as their means ±SDs from *n* = 3 independent samples.

**
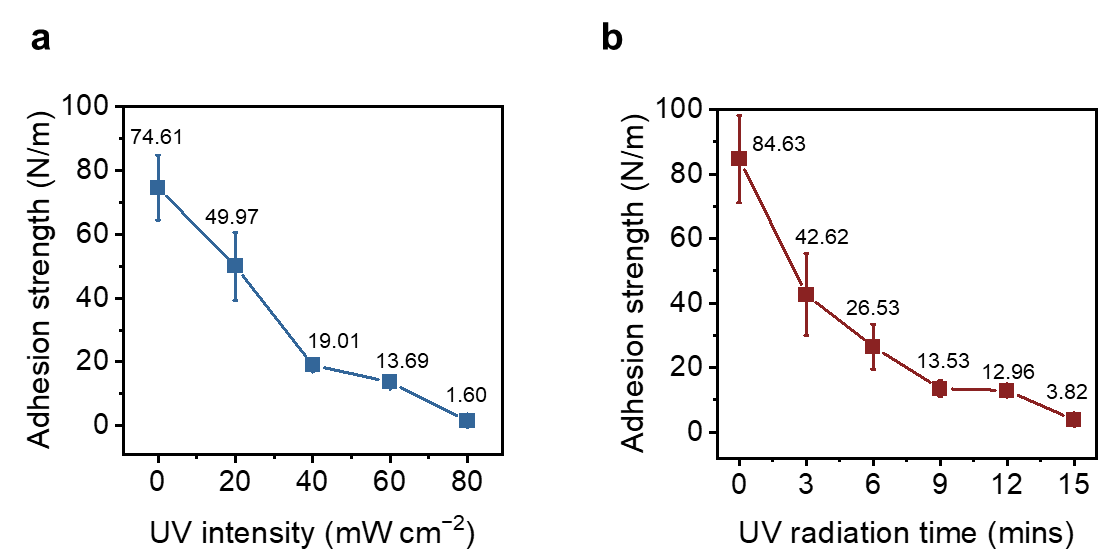
**

**Supplementary Fig. 38 | Adhesion strength as a function of several variables. a**, **b** Relationship of adhesion strength of the CNF-DA/PAA@Fe^3+^ hydrogel to the UV intensity and irradiation time. Data in **a**, **b** are reported as their means ±SDs from *n* = 3 independent samples.

The prepared photo-detachable hydrogel adheres to the glass sheet for 30 min followed by 5 min of UV light exposure (365 nm, 40 mW cm^−2^). We changed the variable and fixed all other variables to the conditions above. The experiment for each value of the variable was repeated with three samples. With a 5 min exposure to UV irradiation, the final adhesion strength decreases as the intensity increases (Supplementary Fig. 38a). In the photo-detaching process, the adhesion strength drops by over 80% after 5 min of UV irradiation (Supplementary Fig. 38b).


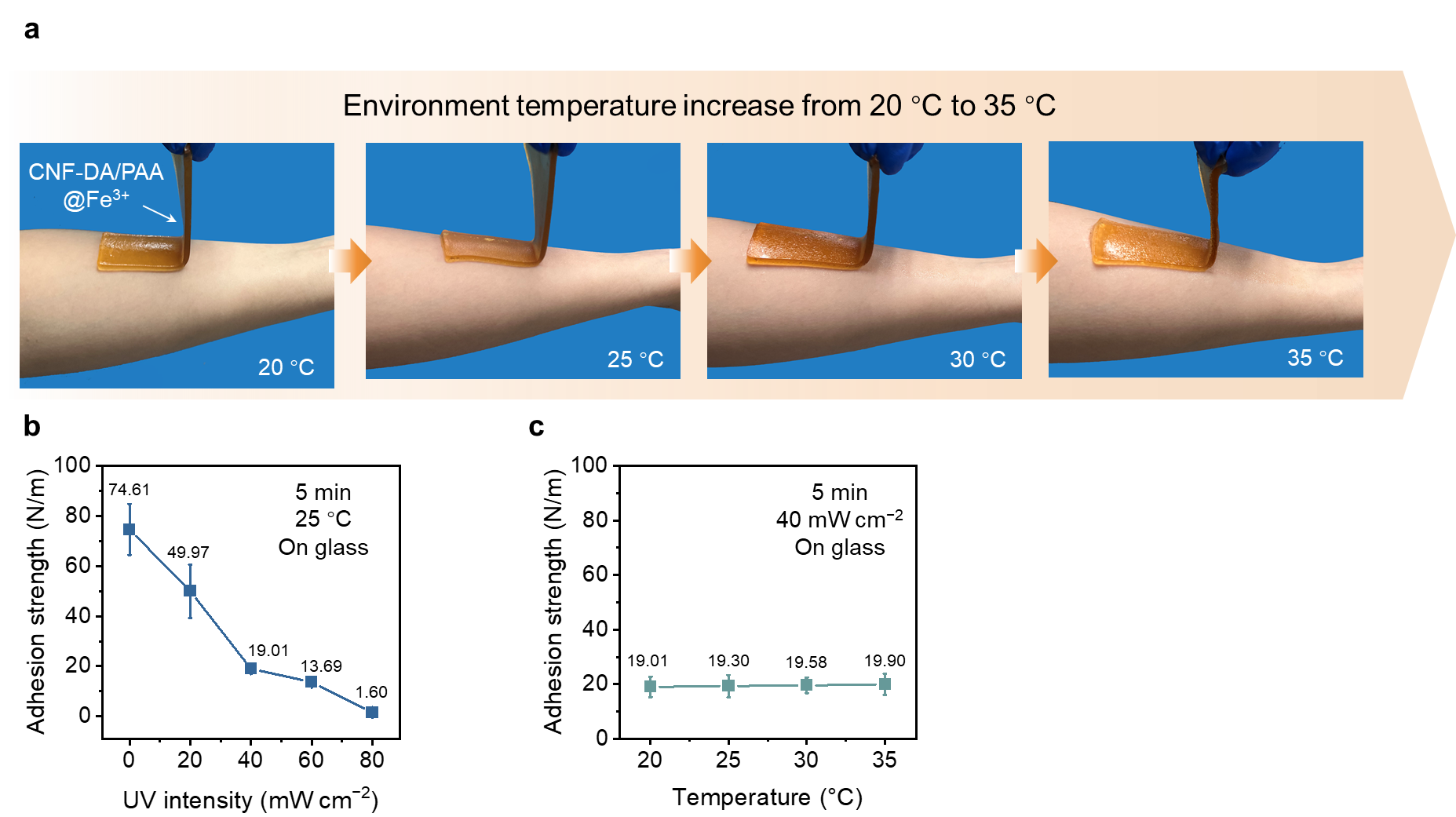


**Supplementary Fig. 39 | Adhesion strength as a function of several variables. a** The peeling process digital images of the CNF-DA/PAA@Fe^3+^ hydrogels with different environment temperatures after UV irradiation. **b,** **c** Relationship of adhesion strength of the CNF-DA/PAA@Fe^3+^ hydrogel to the UV intensity and environment temperature Data in **b**, **c** are reported as their means ±SDs from *n* = 3 independent samples.

The experiment for each value of the variable was repeated with three samples. As shown in Supplementary Fig. 39a, as the environment temperature increases from 0 °C to 35 °C, the CNF-DA/PAA@Fe^3+^ hydrogel still adheres to the skin stably, showing excellent and stable adhesion performance. Furthermore, we investigated the adhesion strength of the CNF-DA/PAA@Fe^3+^ hydrogel on glass under varied UV intensities, and it can be observed that the adhesion strength displays a decreasing trend with the increase of UV intensity (Supplementary Fig. 39b). In particular, at a UV intensity of 40 mW cm^−2^, the adhesion strength of the hydrogel is 19.00 N m^−1^, showing 74.5% of tunability compared to 74.61 N m^−1^ before UV irradiation. It is further observed that the adhesive strength of the hydrogel exhibits negligible fluctuations even at elevated temperatures, revealing that temperature has no significant effect on the adhesive properties of the hydrogel (Supplementary Fig. 39c). In summary, it can be concluded that compared with temperature, the influence of UV light on the CNF-DA/PAA@Fe^3+^ hydrogel is more severe.


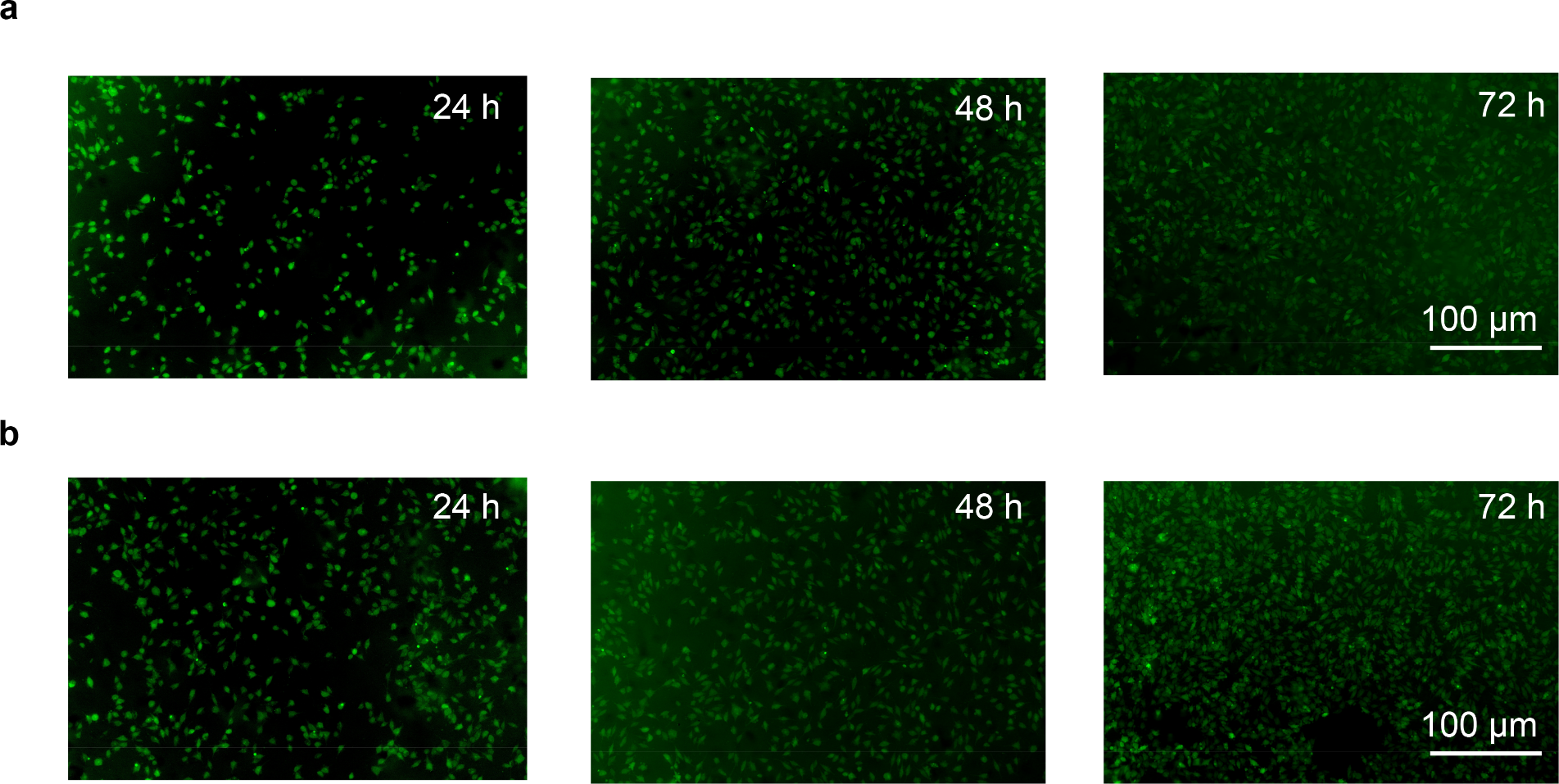


**Supplementary Fig. 40 | Biocompatibility test of the CNF-DA/PAA@Fe^3+^ hydrogel.** **a**, **b** Representative fluorescent staining images of live (green) and dead (red) assay of L-929 fibroblast cells after culturing with non-UV and with UV light, demonstrating a good cytocompatibility of the hydrogel, presenting its conformal and intimate contact with human skin, which is of great significance for e-skin application. Scale bar, 100 μm.

**
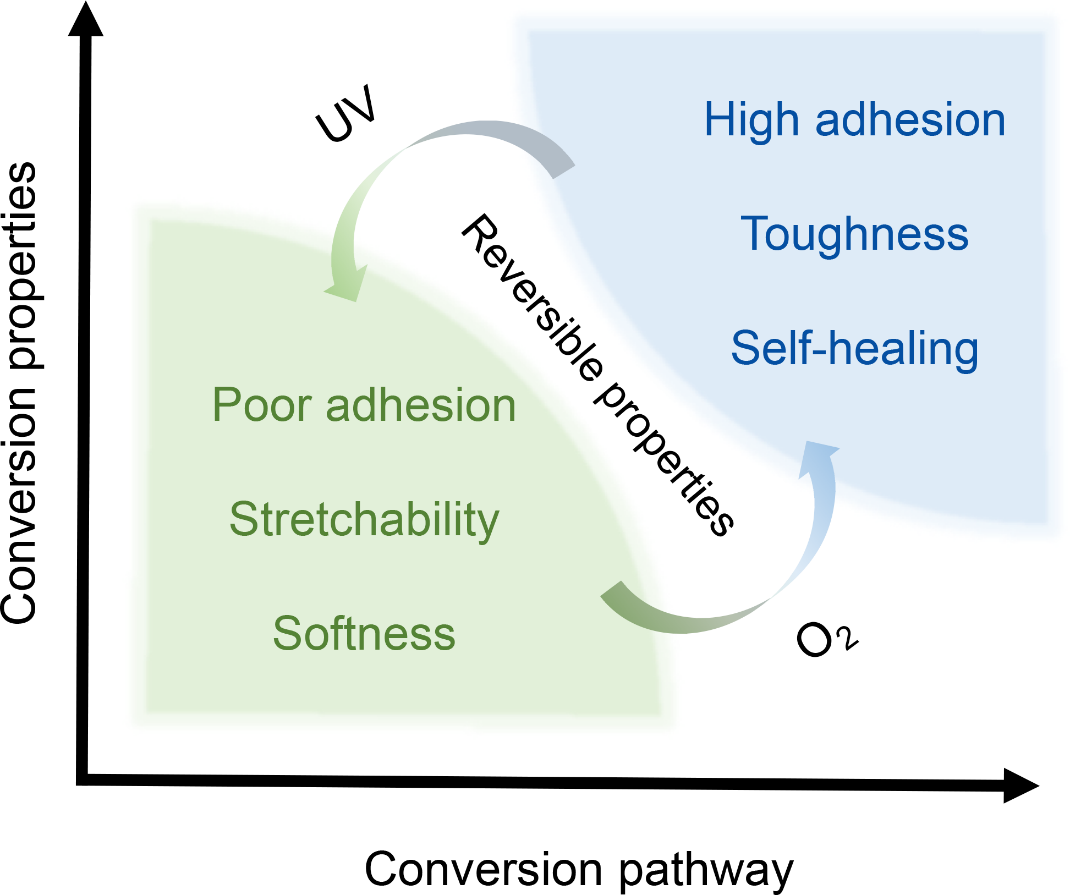
**

**Supplementary Fig. 41 | Schematic diagrams illustrating highly tunable and reversible properties of the CNF-DA/PAA@Fe^3+^ dynamic hydrogel.**
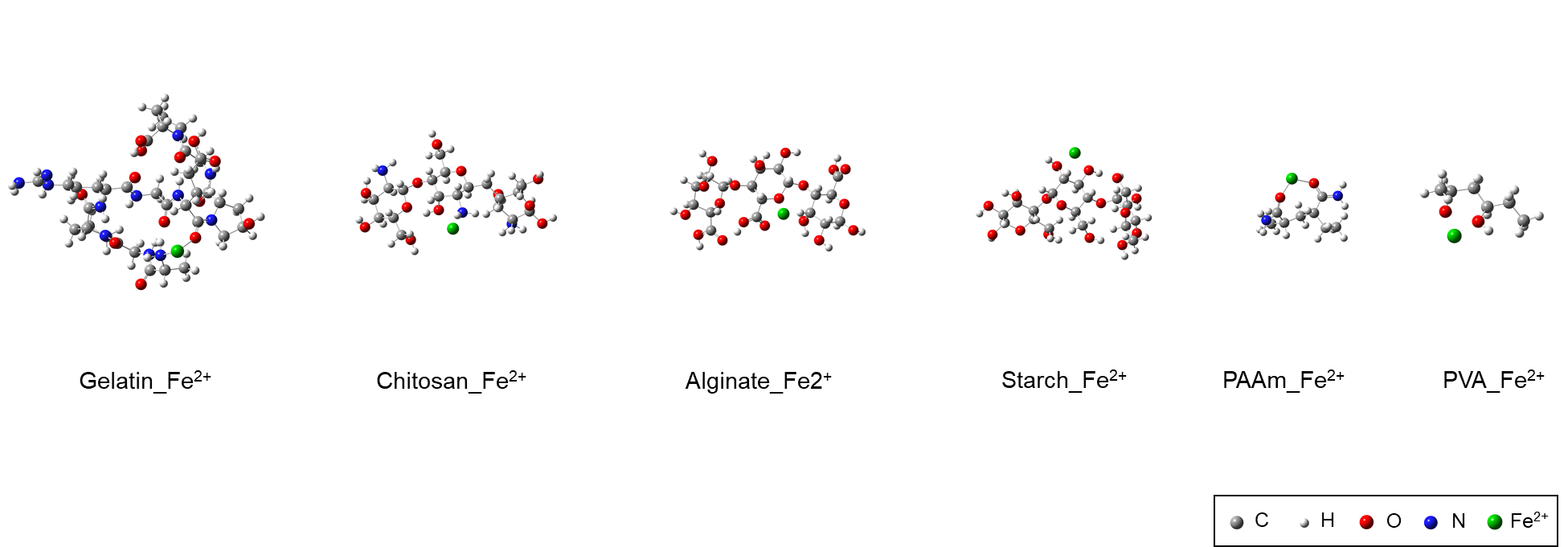
 **Supplementary Fig. 42 | Structural formula of the Fe^2+^ and gelatin, chitosan, alginate, starch, PAAm, and PVA.**


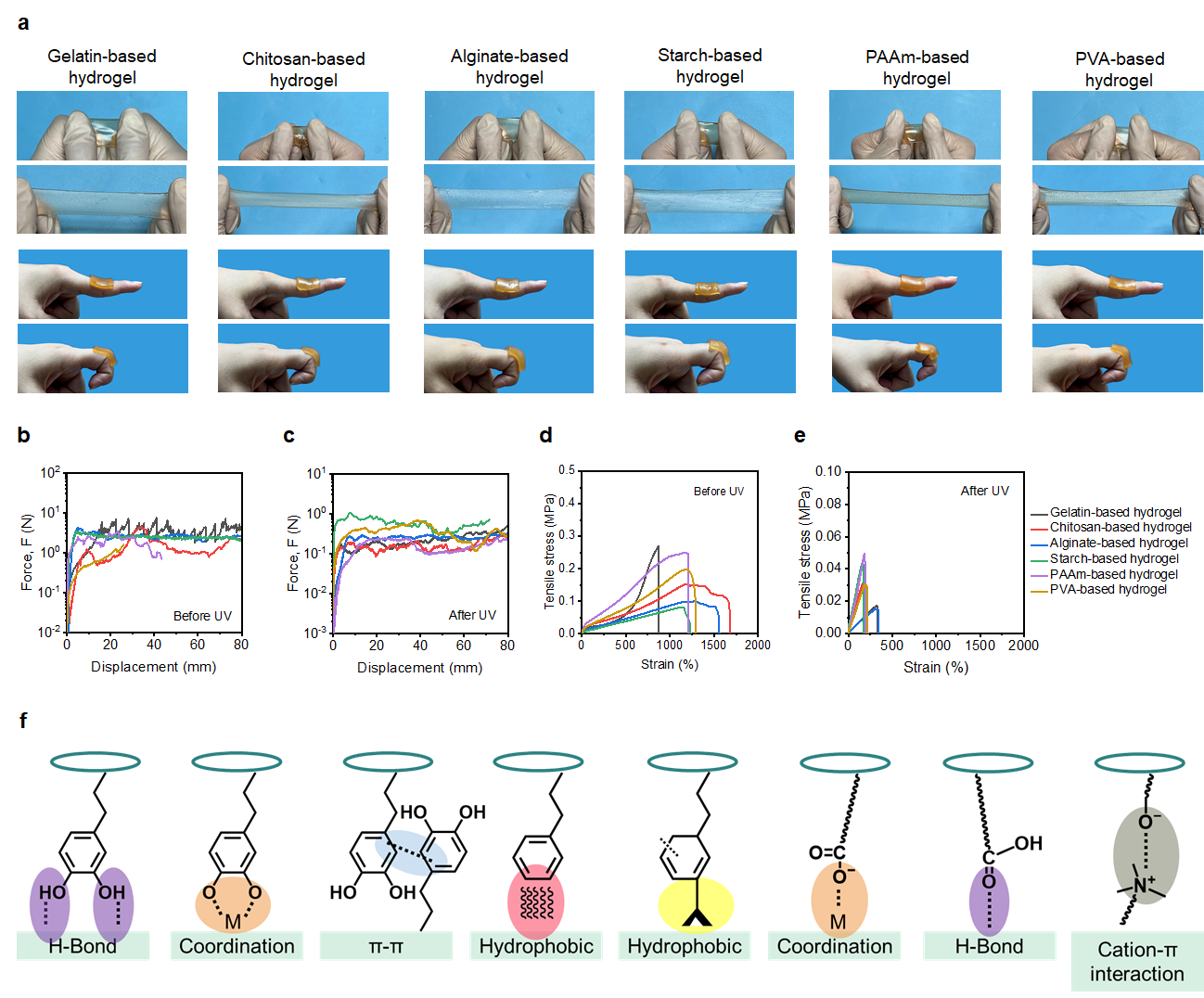


**Supplementary Fig. 43 | Universality of the photo-detachable adhesion strategy.** **a** Digital photographs of the gelatin, chitosan, alginate, starch, PAAm, and PVA-based hydrogels. **b, c** Tensile curves of the gelatin, chitosan, alginate, starch, PAAm, and PVA-based hydrogels before and after UV irradiation. **d, e** 90-degree peeling curves of the gelatin, chitosan, alginate, starch, PAAm, and PVA-based hydrogels before and after UV irradiation. **f** Schematic adhesion mechanism.


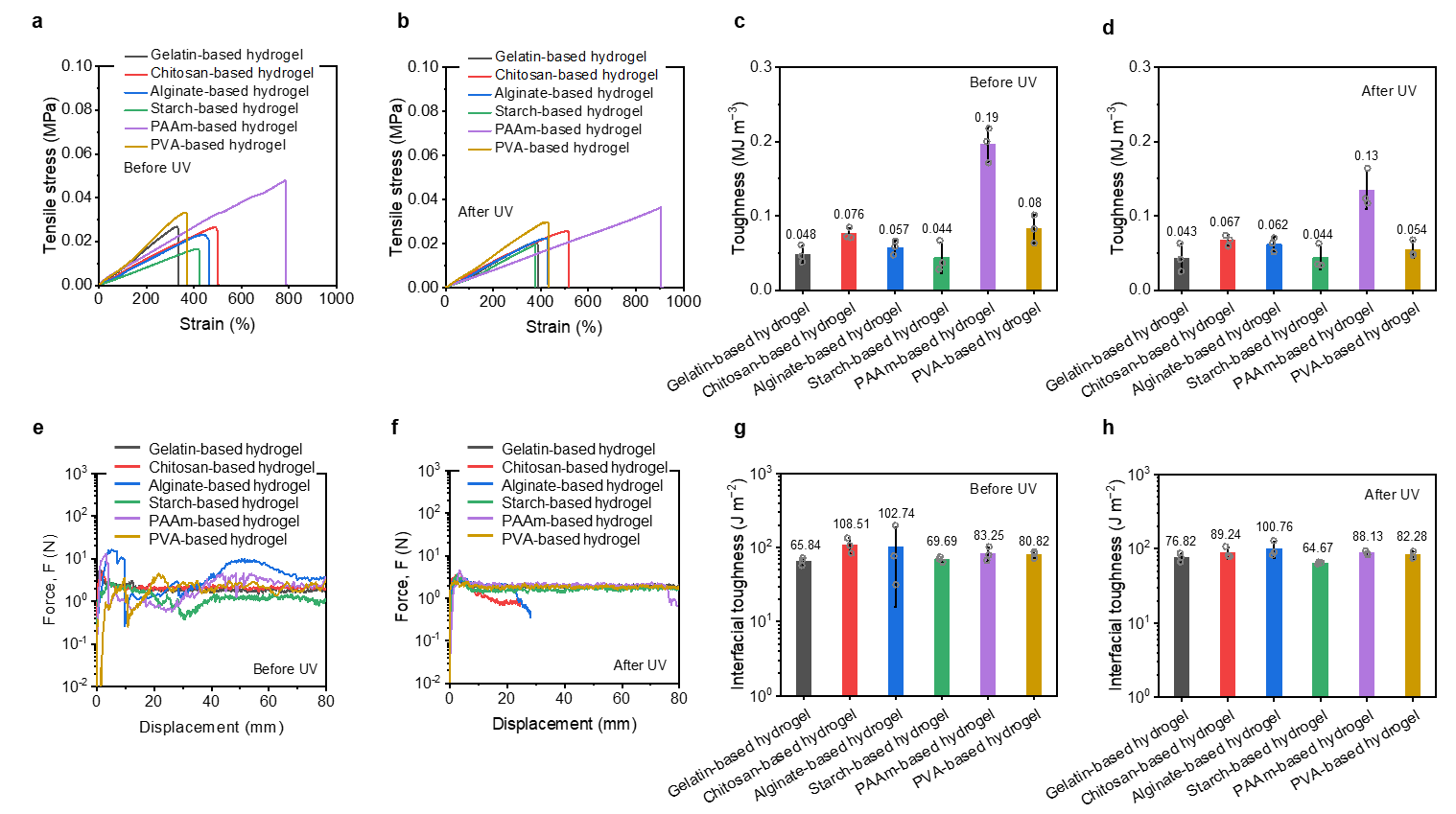


**Supplementary Fig. 44 | Universality of the CNF-mediated photo-detachable adhesion strategy.** **a, b** Tensile curves of the gelatin, chitosan, alginate, starch, PAAm, and PVA-based hydrogels without Fe ions before and after UV irradiation. **c,d** Comparison of the toughness of the gelatin, chitosan, alginate, starch, PAAm, and PVA-based hydrogels without Fe ions before and after UV irradiation (n = 3). **e, f** 90-peeling curves of the gelatin, chitosan, alginate, starch, PAAm, and PVA-based hydrogels without Fe ions before and after UV irradiation on the substrate of glass. **g, h** Comparison of the interfacial toughness of the gelatin, chitosan, alginate, starch, PAAm, and PVA-based hydrogels without Fe ions before and after UV irradiation. Data in **c**, **d** and **g**, **h** are reported as their means ±SDs from *n* = 3 independent samples.


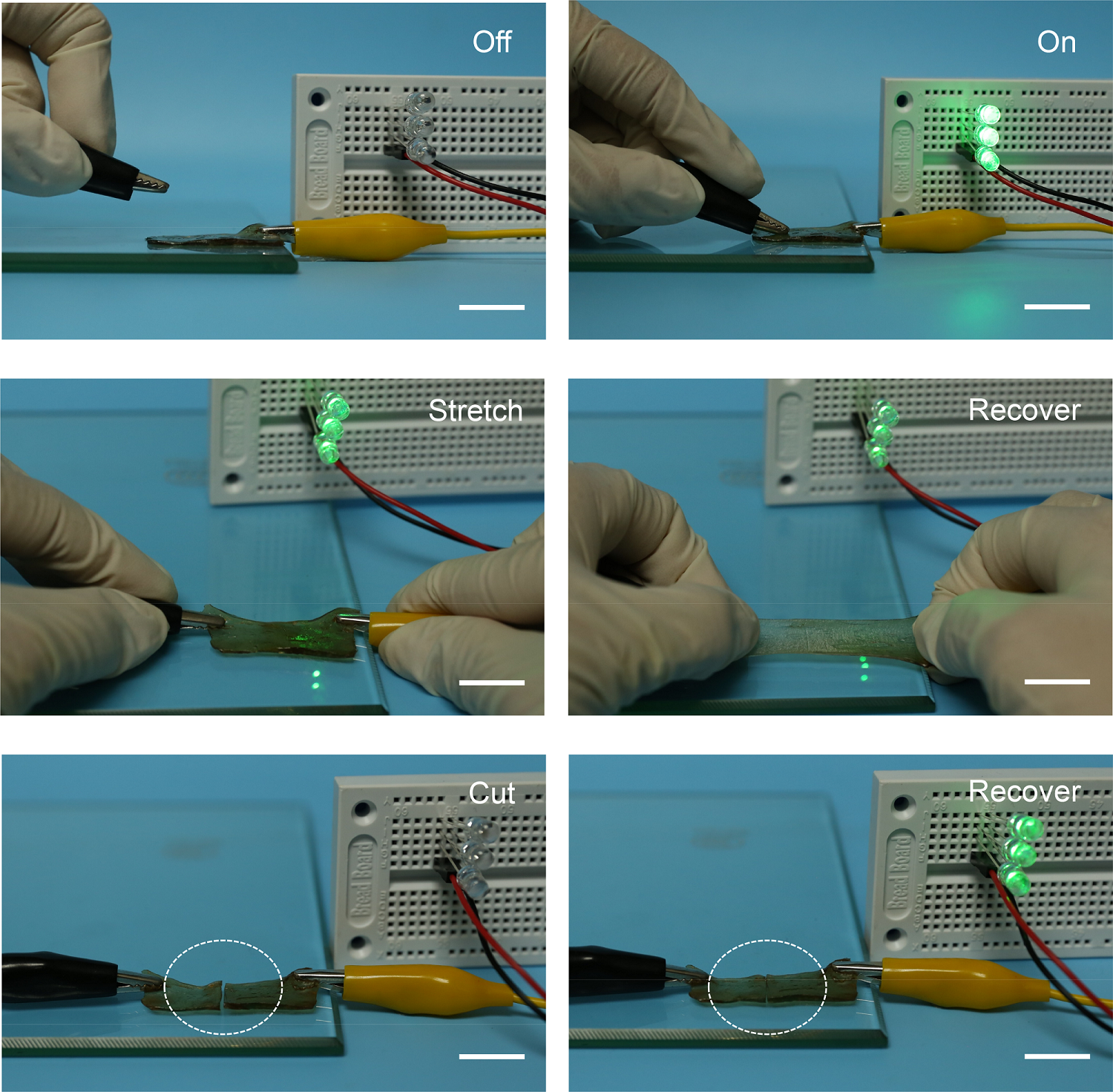


**Supplementary Fig. 45 | Photographs of the photo-detachable CNF-DA/PAA@Fe^3+^ hydrogel to light up LEDs under different conditions.** Scale bar, 1 cm.


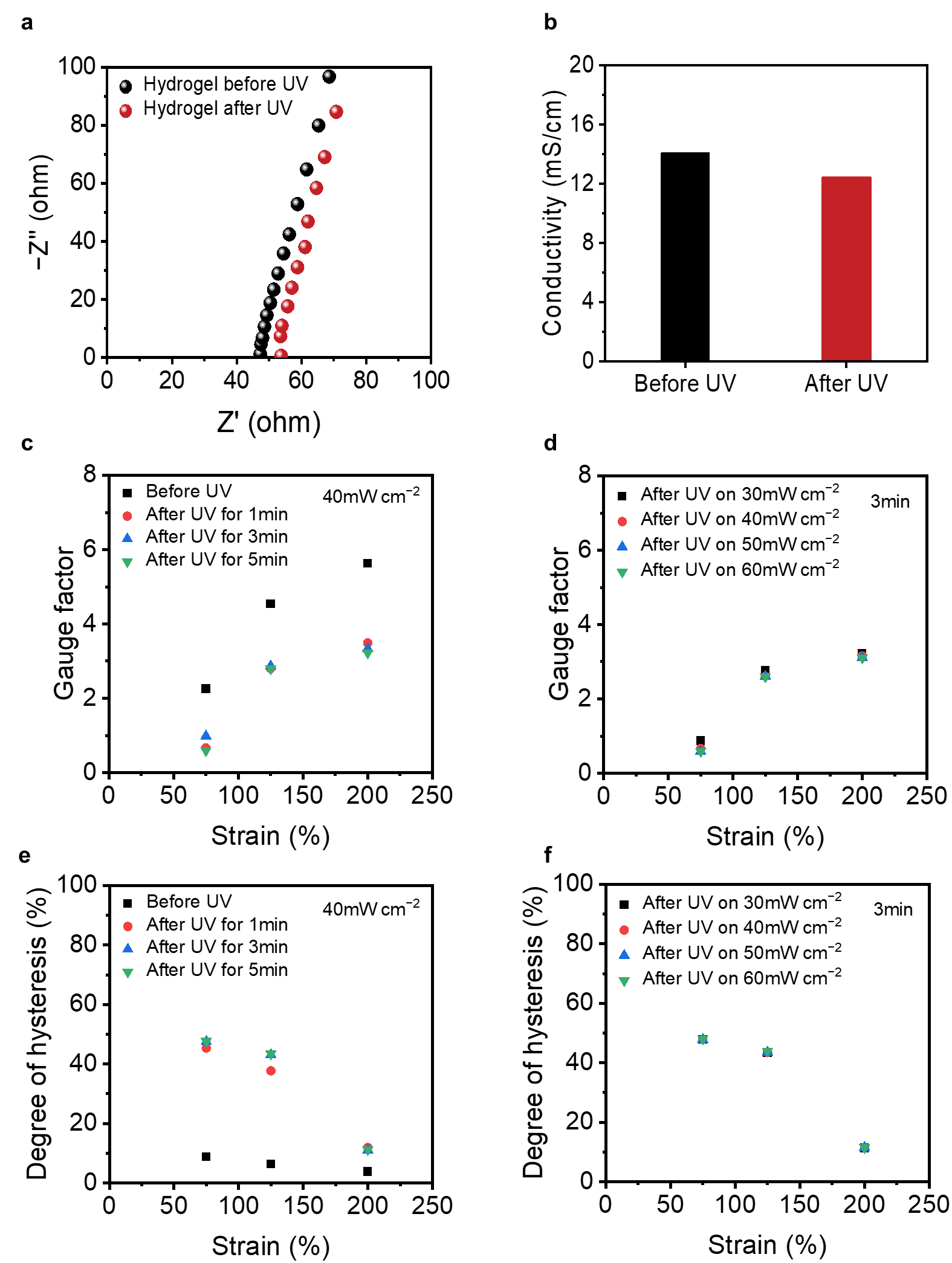


**Supplementary Fig. 46 | Electrical performance of the CNF-DA/PAA@Fe^3+^ hydrogel.** **a** EIS Nyquist plot of the photo-detachable hydrogel before and after UV irradiation. **b** Ionic conductivity of the photo-detachable hydrogel before and after UV irradiation. **c** Gauge factor curves of the photo-detachable hydrogel before and after UV irradiation with different times at 40 mW cm^−2^. **d** Gauge factor curves of the photo-detachable hydrogels after 3 min UV irradiation with different UV irradiation intensities. **e** Degree of hysteresis curves of the photo-detachable hydrogel before and after UV irradiation with different times at 40 mW cm^−2^. **f** Degree of hysteresis curves of the photo-detachable hydrogel after 3 min UV irradiation with different UV irradiation intensities.


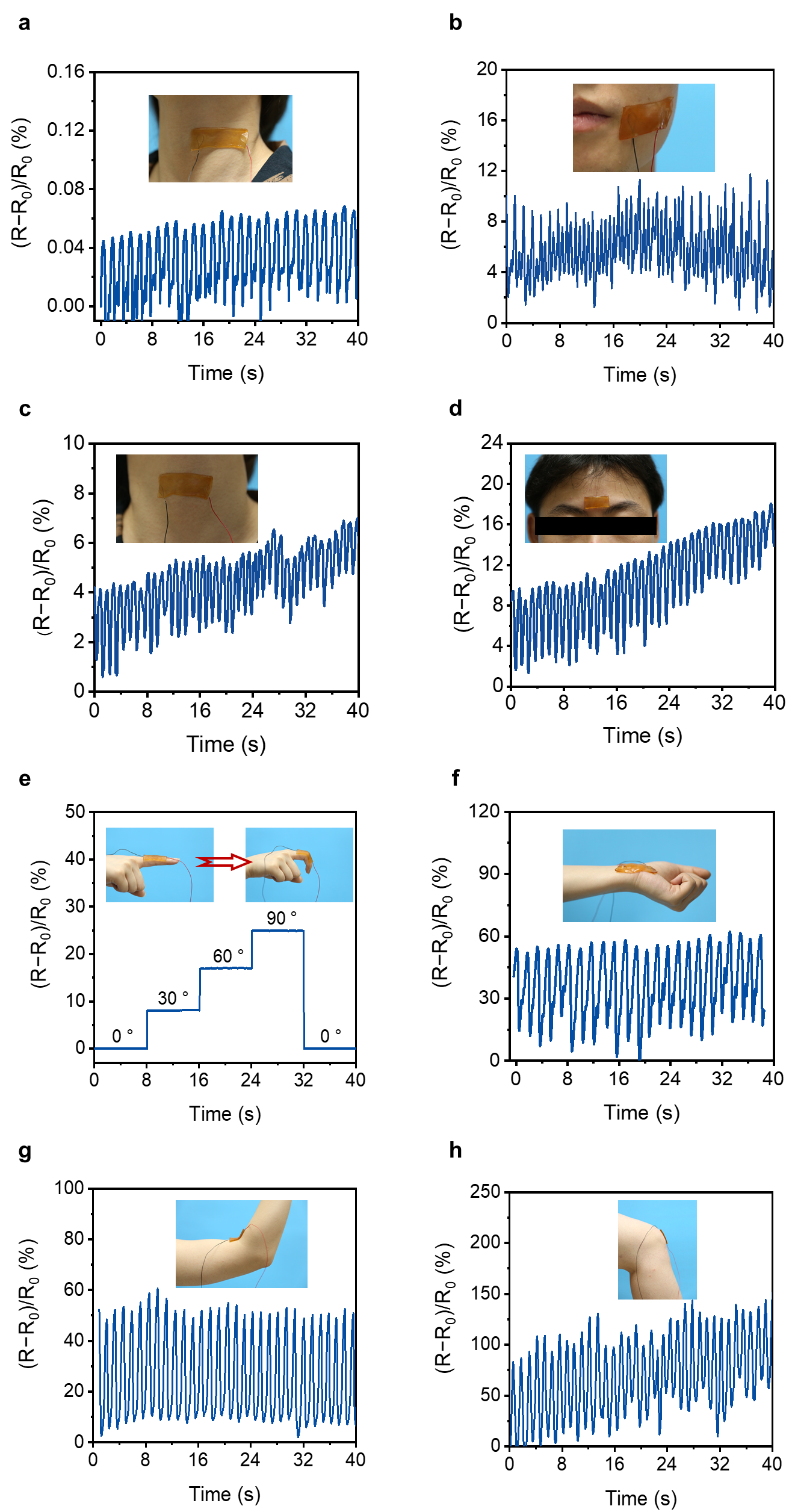


**Supplementary Fig. 47 | Application of the CNF-DA/PAA@Fe^3+^ hydrogel as a self-powered e-kin for whole-body physiological and motion monitoring.** Real-time monitoring of human physical activities based on wearable sensors assembled from photo-detachable hydrogels. **a**−**d** Monitoring various subtle actions and the corresponding images (inset) (including pulse, smiling, swallowing, and frowning). **e**−**h** Monitoring large-scale motion of human joints and the corresponding images (inset) (including finger, wrist, elbow, and knee).


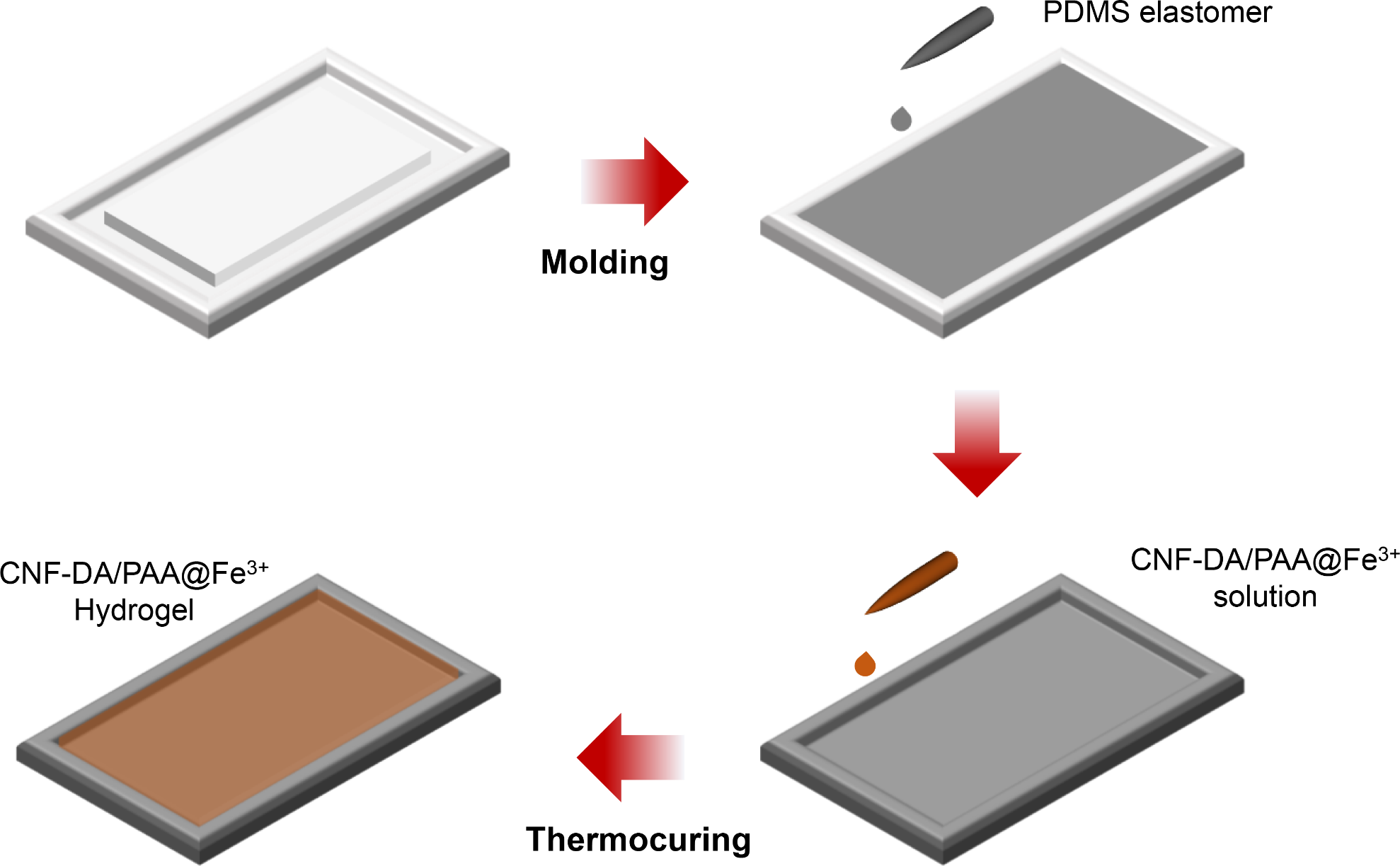


**Supplementary Fig. 48 | Schematic illustration of fabricating the two-layer structured photo-detachable adhesion-triboelectric nanogenerator (PdA-TENG).** The PdA-TENG is composed of a silicone rubber layer (polydimethylsiloxane, PDMS) as the tribo-negative material and the hydrogel (CNF-DA/PAA@Fe^3+^) as both the ionic current collector and the photo-detachable substrate.


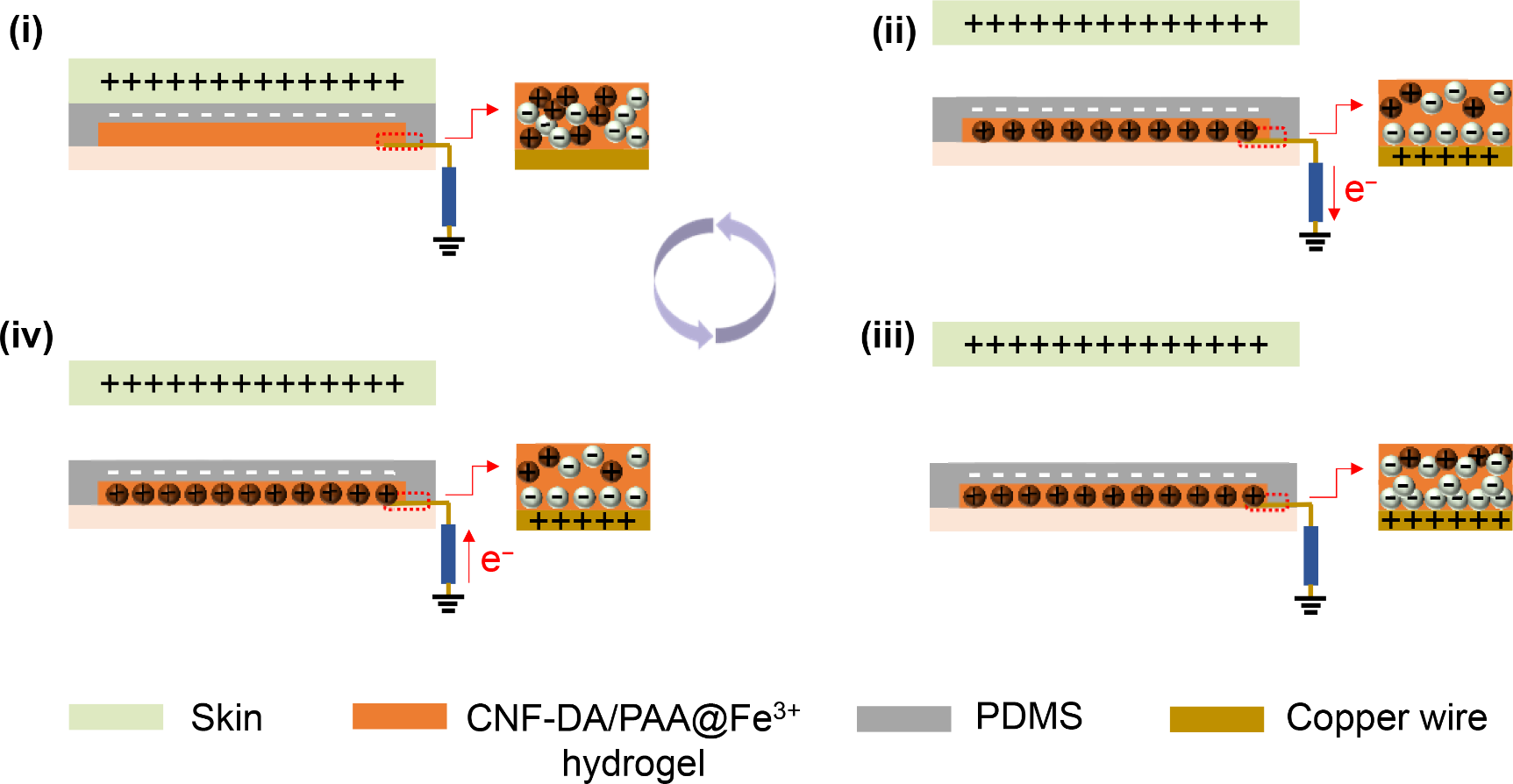


**Supplementary Fig. 49 | Working principle of the single-electrode PdA-TENG with two-layer structure.**


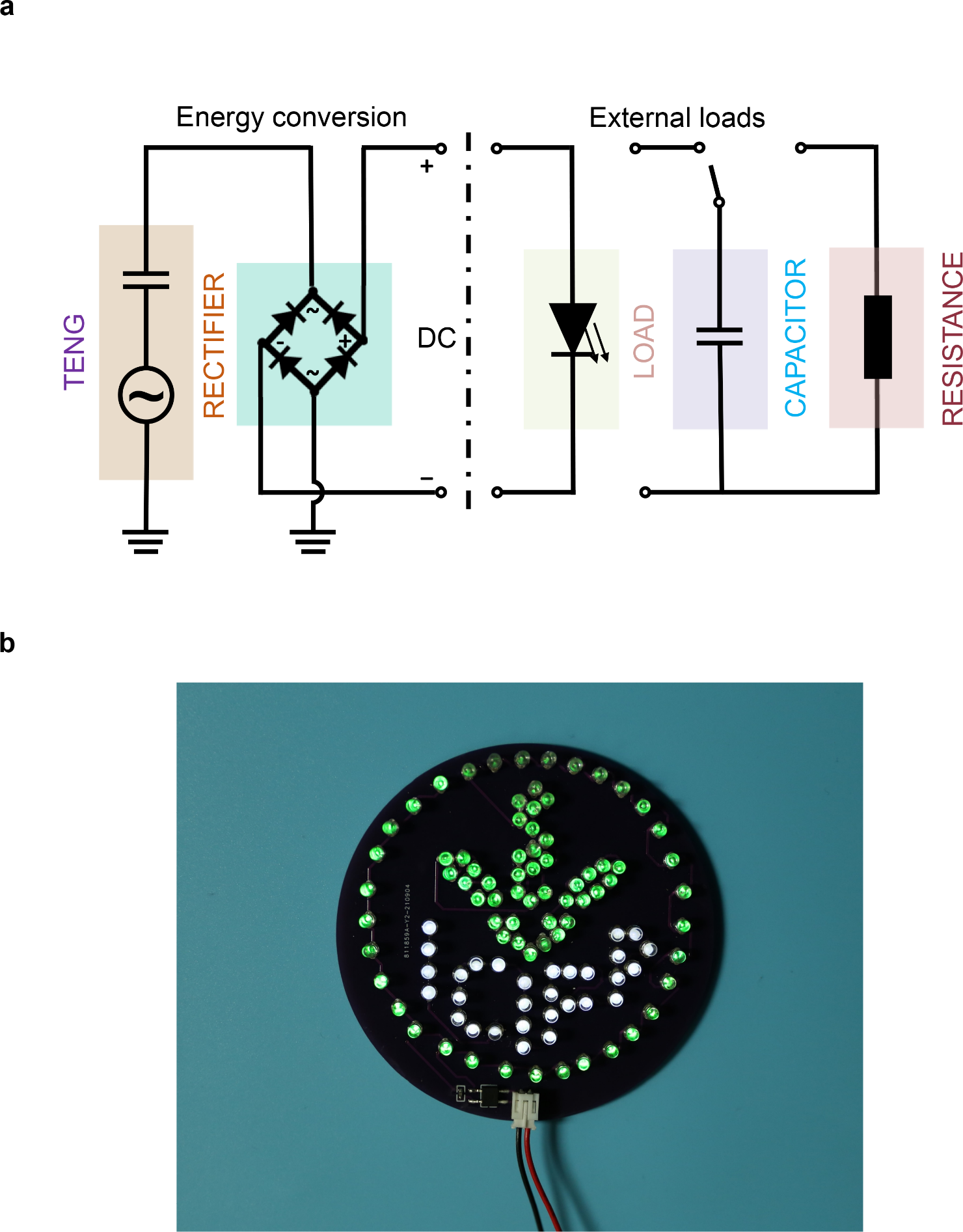


**Supplementary Fig. 50 | Schematic design of the PdA-TENG circuit diagram.** **a** Schematic diagram of the circuit with external loads. **b** Photograph of 104 LEDs powered by hand tapping. The schematic diagram with a rectifier circuit connected with external loads such as resistors, LEDs, electronics, and capacitors, demonstrating the real-time application of the PdA-TENG. Supplementary Movie 5 displays that the PdA-TENG can light up 104 LEDs of green and white.


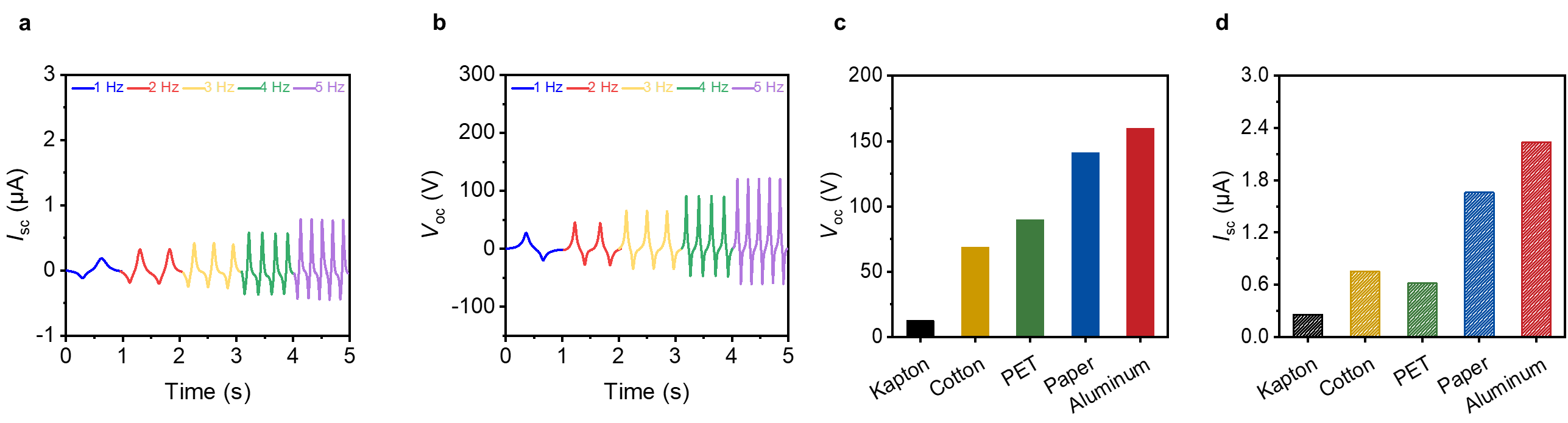


**Supplementary Fig. 51 | The electrical output performance of photo-detachable adhesion-triboelectric nanogenerator (PdA-TENG).** **a**, **b** Frequency-response characteristics of the CNF-DA/PAA@Fe^3+^ hydrogel as a self-powered e-skin (including *I*_sc_, *V*_oc_).**c**, **d** *V*_oc_ and *I*_sc_ output of the PdA-TENG with different sets of tribo-positive materials. As a result of the feasibility of contact electrification with any two different layers, the PdA-TENG can produce stable voltage/current outputs through the relative motion with various other materials^13^.


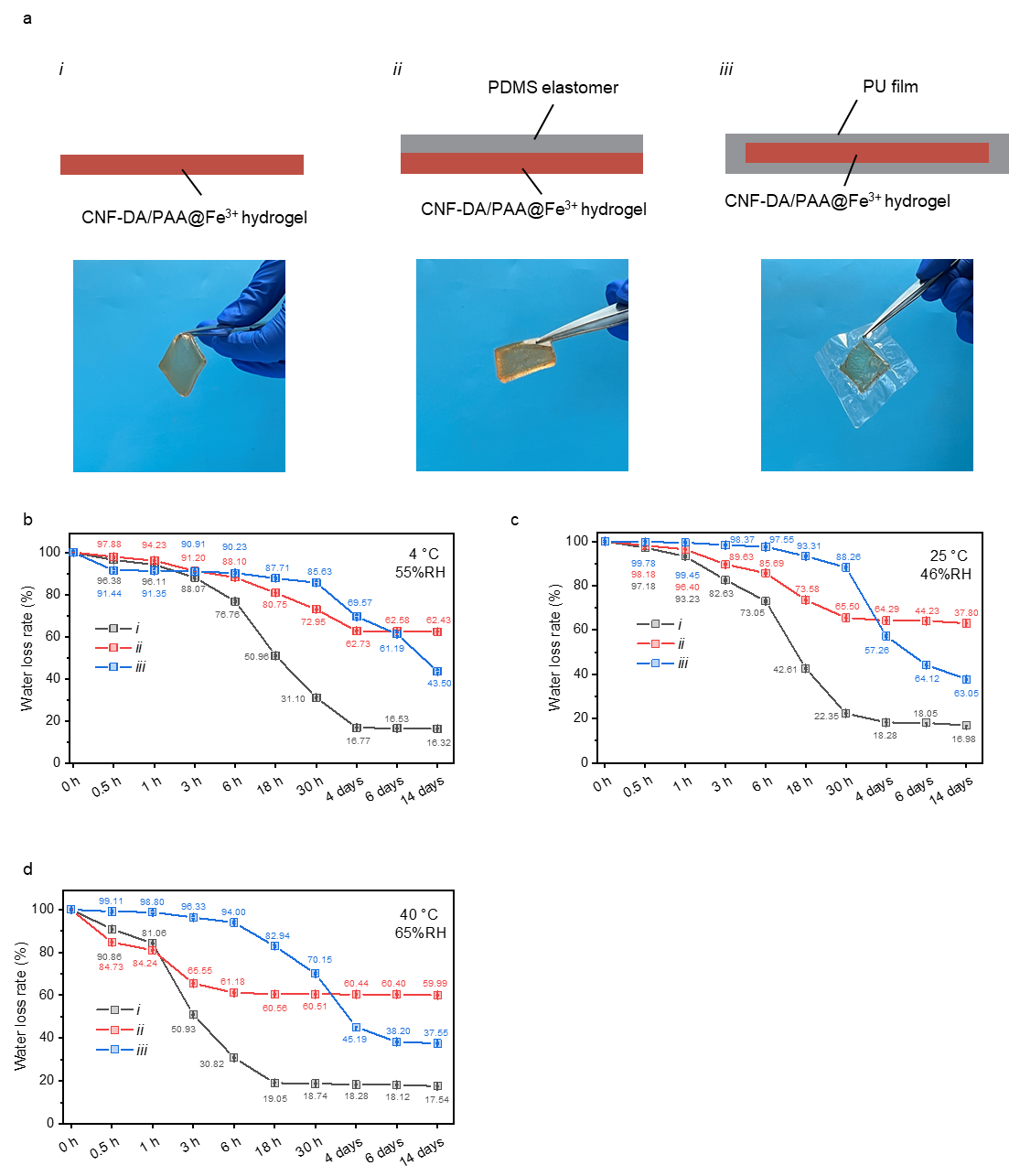


**Supplementary Fig. 52 |** **Water retention properties of the CNF-DA/PAA@Fe^3+^ hydrogel.** **a** Encapsulation methods of the hydrogel to improve its water retention performance: top-down encapsulation and wrap-around encapsulation. **b-d** The water loss curves of different hydrogel e-skins at varying temperatures of (b) 4 °C, (c) 25 °C, and (d) 40 °C, respectively. Data in **b-d** are reported as their means ±SDs from *n* = 3 independent samples.

To stabilize the overall performance of our hydrogel as e-skin, we carried out two packaging methods to improve the water retention of the hydrogel materials: top-down encapsulation and wrap-around encapsulation. The water retention properties of the hydrogels were evaluated in different temperature environments (4 °C, 25 °C, 40 °C). As shown in Supplementary Fig. 52, pure hydrogels show weaker water retention (16.32% for 4 °C, 16.98% for 25 °C, 17.54% for 40 °C), and the top-down encapsulation hydrogel e-skins exhibit the excellent water retention in comparison with the wrap-around encapsulation (62.43% *VS* 43.50% for 4 °C, 63.05% *VS* 37.80% for 25 °C, 59.99% *VS* 37.55% for 40 °C). In our E-skin configuration, we adapted top-down encapsulation to improve the water retention of our hydrogel materials for potential long-term use.


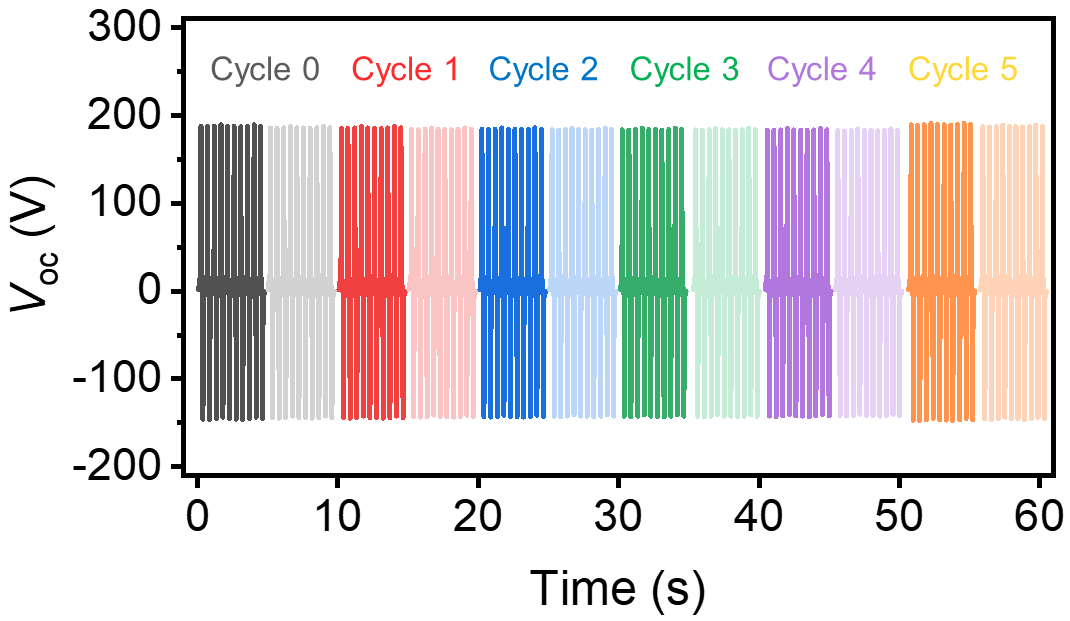


**Supplementary Fig. 53 | The voltage output of the PdA-TENG with repeated cycles contacting under UV light.** Stable voltage output demonstrated good stability and reusability of the PdA-TENG.


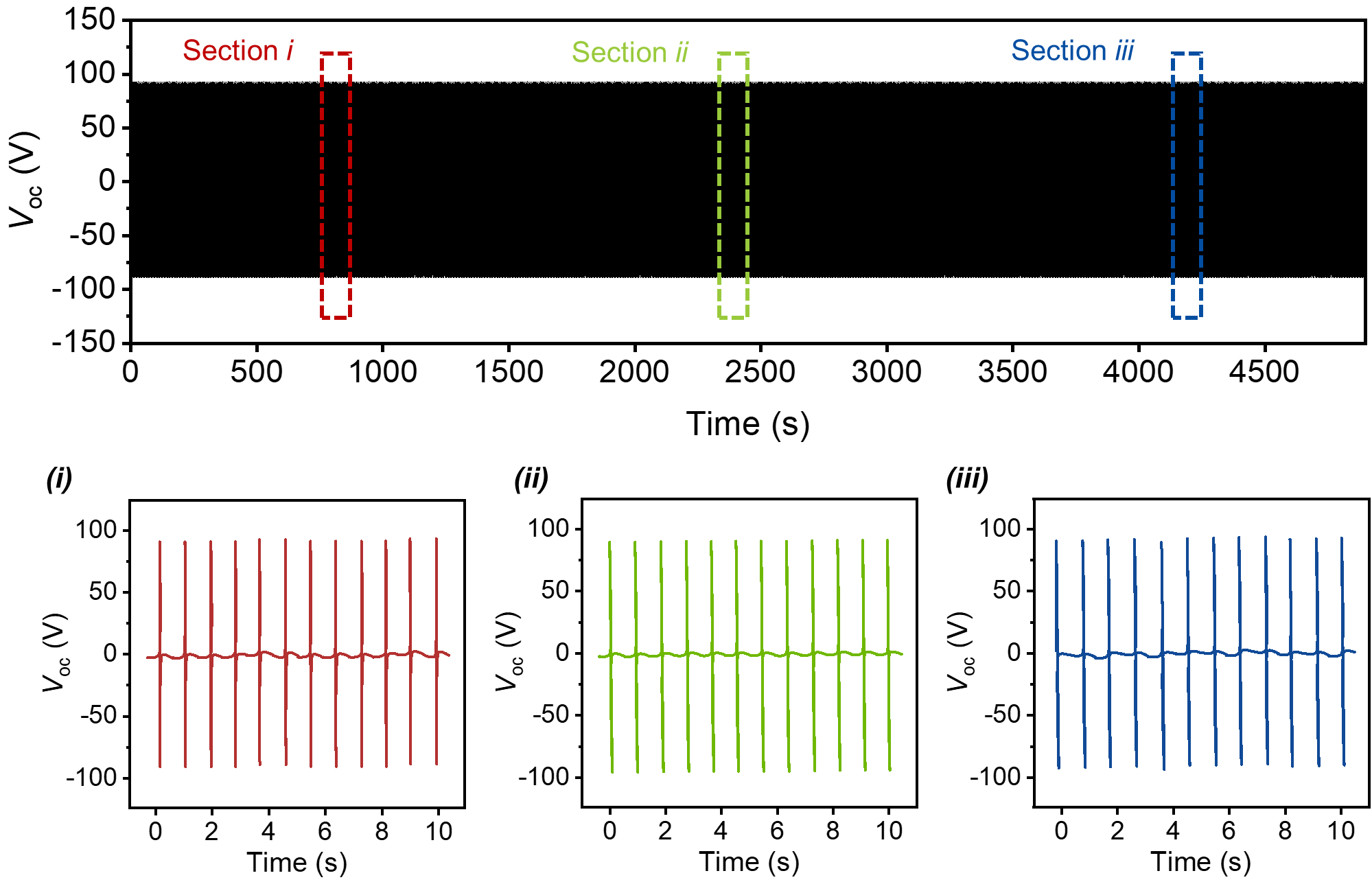


**Supplementary Fig. 54 | Stability and durability test of the PdA-TENG.** The long-term motion cycles were performed to demonstrate the durability of the PdA-TENG. The open-circuit voltage demonstrates a low output fluctuation after 4800 cycles of the repeated separation-contact motion, indicating outstanding stability of the PdA-TENG.


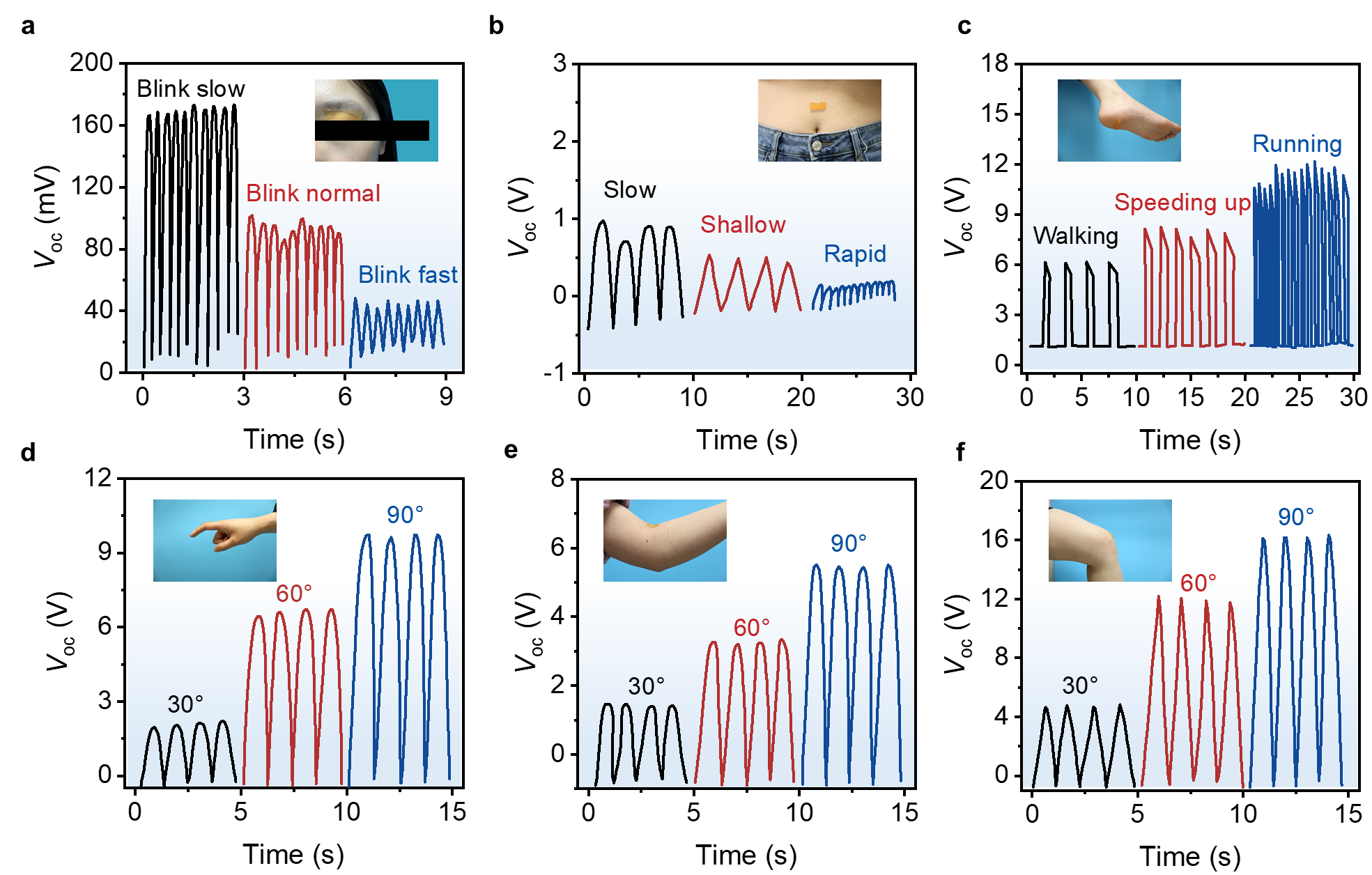


**Supplementary Fig. 55 | PdA-TENG is used as a self-powered e-kin device for whole-body physiological and motion monitoring.** **a** Monitoring blinking behavior is performed by attaching an e-skin to the eyelid. The photograph of the detection part of the eyelid is inserted. **b** Abdominal respiratory monitoring is performed by applying an e-skin on the human belly, including slow, normal (shallow), and rapid breathing. The photograph of the detection part of the belly is inserted. **c** Detection of the foot motion by fixing an e-skin on the heel. The photograph of the detection portion of the heel is inserted. **d** Monitoring of the bending angles of the fingers by attaching an e-skin to the knuckle. The photograph of the detection portion of the knuckle is inserted. **e** Monitoring of the arm flexion by attaching an e-skin to the elbow. The photograph of the detection portion of the elbow is inserted. **f** Monitoring of the angle of the leg swing by attaching an e-skin to the knee. The photograph of the detection portion of the knee is inserted.


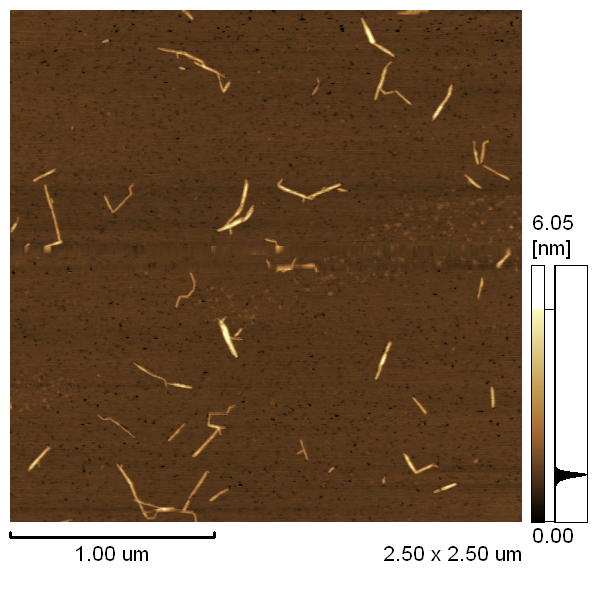


**Supplementary Fig. 56 | AFM image of the CNFs.** The width of individual nanofibrils is 6.05 nm, and the length of individual nanofibrils is estimated to range from 100-500 nm. Scale bar, 1.00 μm.


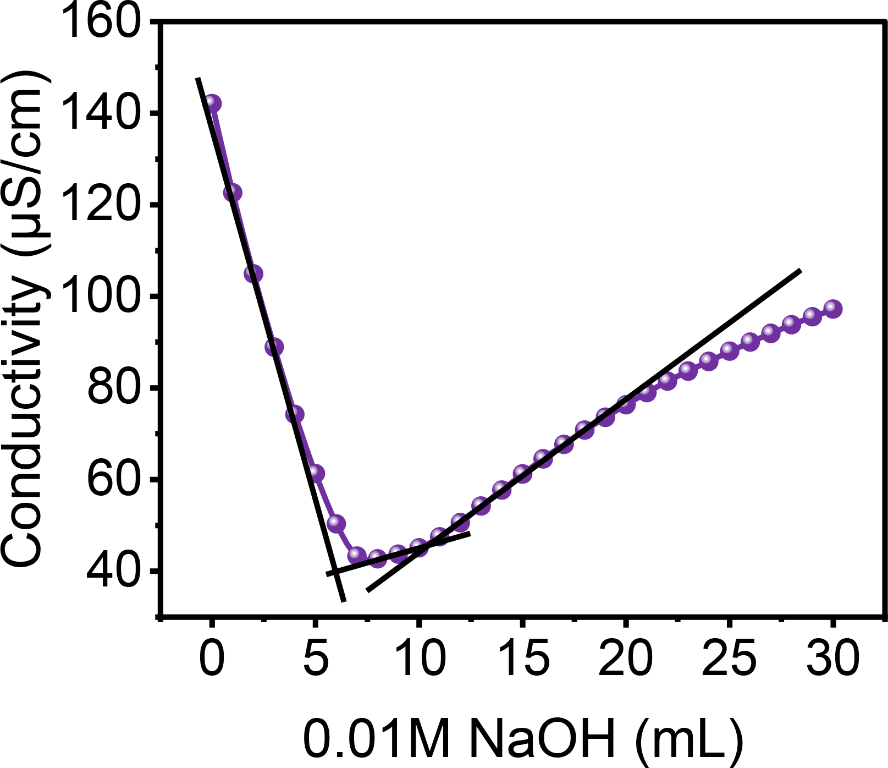


**Supplementary Fig. 57 | Typical conductometric titration curve of the CNFs for determining the surface charge density.** After TEMPO-catalyzed oxidation, C-6 hydroxyl groups of CNFs are converted to negatively charged carboxyl groups. To acquire the exact charge density, a conductive titration is calculated to be 1.2 mmol/g.

**
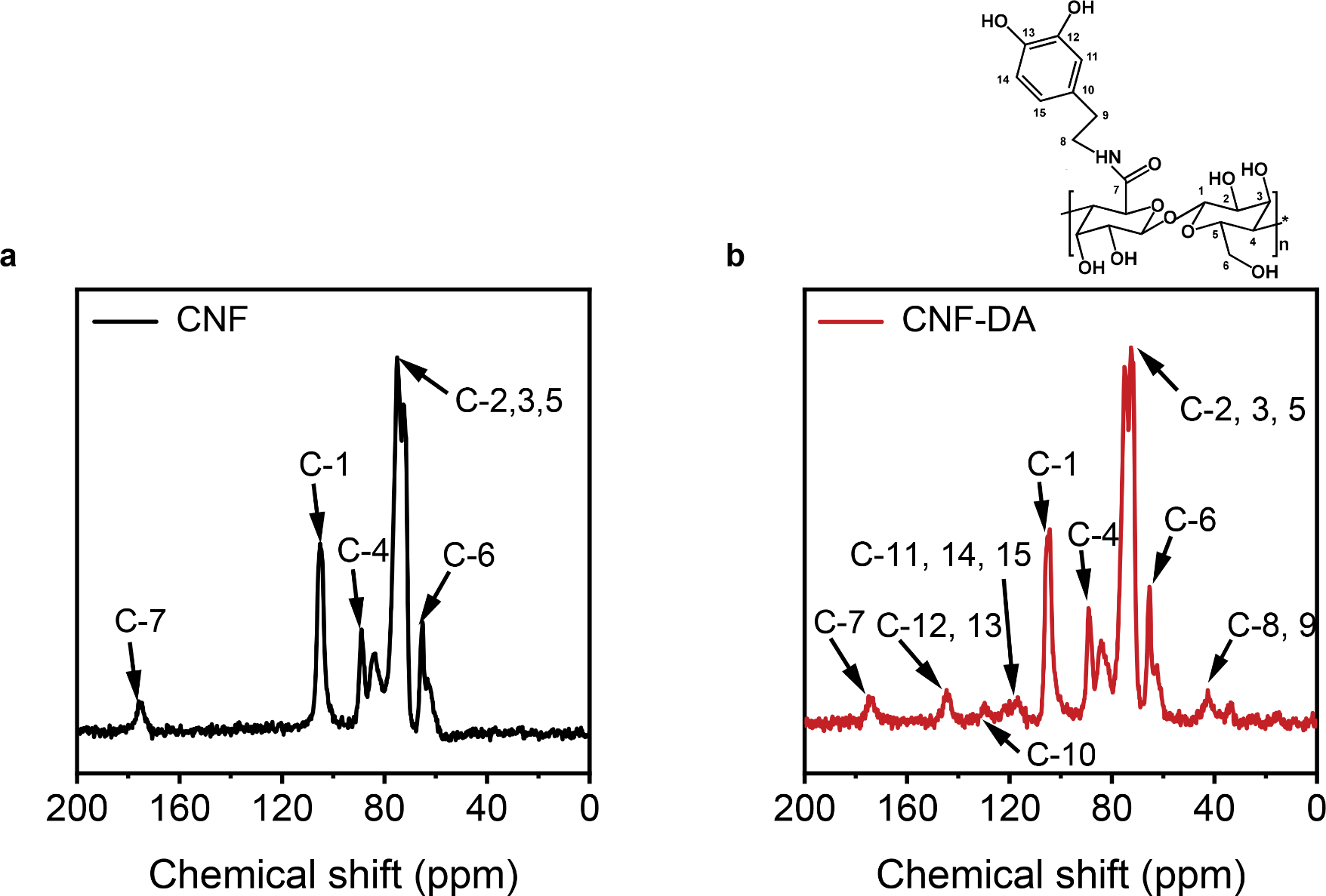
**

**Supplementary Fig. 58 | The structure of CNF, CNF-DA. a**, **b** The solid-state ^13^C NMR spectra of CNF, CNF-DA. The solid-state ^13^C NMR spectra of CNF−COOH (Supplementary Fig. 58a) demonstrated the characteristic chemical shifts of cellulose at 66.18 ppm (C-6), 73.44 ppm, 75.85 ppm, 76.05 ppm (C-2, C-3, and C-5), 89.77 ppm (C-4), and 105.85 ppm (C-1). Furthermore, a distinct chemical shift is observed at 171.25 ppm (C-7, −COOH). Featured chemical shifts of CNF-DA are similar to those of CNF−COOH, indicating that the molecular structure of CNF−COOH is preserved after grafting DA to CNF chains (Supplementary Fig. 58b). However, the new peaks of 20−50 ppm (−CH_2_−) and 100−160 ppm (benzene ring) appear in the ^13^C NMR spectra of CNF-DA, which are attributed to the grafting of DA on CNF−COOH^14^.

**Supplementary Fig. 59 | Fourier transform infrared (FTIR) spectra of CNF-DA/PAA@Fe^3+^ hydrogel, CNF/PAA@Fe^3+^ hydrogel, and PAA@Fe^3+^ hydrogel.**

**Supplementary Table**

**Supplementary Table 1 | Comparison of the tunable ratio in adhesive strength of the CNF-DA/PAA@Fe^3+^ hydrogel with other adhesive materials.**

| **Refs** | **Adhesive materials** | **External stimuli** | **Adhesive strength before external stimuli (N/m)** | **Adhesive strength after external stimuli (N/m)** | **Upper limit adhesive strength adjustment proportion (%)** | **Lower limit adhesive strength adjustment proportion (%)** |
| --- | --- | --- | --- | --- | --- | --- |
| This work | CNF-DA/PAA@Fe^3+^ hydrogel | UV light | 80~49 | 5~5.9 | 94.0 | 93 |
| 22 | PS-*b*-P2VP | Humidity | 275~300 | 213~282 | 23 | 6 |
| 23 | OP-CPC films | Water | 175~185 | 100~120 | 42.9 | 35.1 |
| 24 | PAAm hydrogel | NaClO4/dopamine | 340~280 | 140~180 | 58.8 | 35.7 |
| 25 | *G. aparine* leaf hooks | Mechanical interlocker | 0.53~0.25 | 0.18~0.12 | 66.0 | 52.0 |
| 7 | Stitching cellulose solution | pH | 248~150 | 75~50 | 69.8 | 66.7 |
| 26 | AL-Cu@W/EG-PAM | APS | 27.5~25 | 10~7.5 | 70.0 | 63.6 |
| 27 | PEDOT:PSS films | H_2_SO_4_ | 20~2.5 | 5.3~1 | 73.5 | 60 |
| 11 | PAAm@*κ*-carrageenan hydrogel | Electrochemistry treating | 200~300 | 1400~900 | 85.7 | 66.7 |
| 28 | BAP film | Temperature | 32.7~45 | 10~5 | 88.9 | 69.4 |
| 12 | Fe^3+^-Citrate | UV light | 200~50 | 10~25 | 90.0 | 50.0 |
| 29 | Sticky-slippy skin | Crosslinking agent | 133~120 | ~10 | 92.5 | 91.9 |
| 30 | Magnetic micropillars | Magnetic fields | 11~9 | 0.7~0.69 | 93.6 | 92.2 |

**Supplementary References**

1. Li, Q., Wang, A., Long, K., He, Z. & Cha, R. Modified fenton oxidation of cellulose fibers for cellulose nanofibrils preparation. *ACS Sustain*. *Chem*. *Eng.* **7,** 1129−1136 (2018).

2. Pignatello, J. J., Oliveros, E. & MacKay, A. Advanced oxidation processes for organic contaminant destruction based on the fenton reaction and related chemistry. *Crit*. *Rev*. *Environ*. *Sci. Tec.* **36,** 1−84 (2006).

3. Vijay, P., Batchelor, W. & Saito, K. One-pot treatment of cellulose using iron oxide catalysts to produce nanocellulose and water-soluble oxidised cellulose. *Carbohydr*. *Polym*. **282,** 119060 (2022).

4. Duan, L., Liu, R. & Li, Q. A more efficient fenton oxidation method with high shear mixing for the preparation of cellulose nanofibers. *Starch - Stärke* **72,** 11−12 (2020).

5. Wang, Q. et al. Ultrafast gelling using sulfonated lignin-Fe^3+^ chelates to produce dynamic crosslinked hydrogel/coating with charming stretchable, conductive, self-healing, and ultraviolet-blocking properties. *Chem*. *Eng*. *J*. **396,** 125341 (2020).

6. Liang, L., Niu, L., Wu, T., Zhou, D. & Xiao, Z. Fluorine-free fabrication of MXene via photo-fenton approach for advanced lithium-sulfur batteries. *ACS Nano* **16,** 7971−7981 (2022).

7. Kriechbaum, K. & Bergstrom, L. Antioxidant and UV-blocking leather-inspired nanocellulose-based films with high wet strength. *Biomacromolecules* **21,** 1720−1728 (2020).

8. Shannon, D. P. et al. Modular Synthesis and Patterning of High-Stiffness Networks by Postpolymerization Functionalization with Iron-Catechol Complexes. *Macromolecules* **56**, 2268-2276 (2023).

9. Zhang, Z. et al. Eco-Friendly, Self-Healing Hydrogels for Adhesive and Elastic Strain Sensors, Circuit Repairing, and Flexible Electronic Devices. *Macromolecules* **52**, 2531-2541 (2019).

10. Deng, J. et al. A bioinspired medical adhesive derived from skin secretion of andrias davidianus for wound healing. *Adv.* *Funct.* *Mater.* **29**, 1809110 (2019).

11. Cui, C., Shao, C., Meng, L. & Yang, J. High-strength, self-adhesive, and strain-sensitive chitosan/poly (acrylic acid) double-network nanocomposite hydrogels fabricated by saltsoaking strategy for flexible sensors. *ACS* *Appl.* *Mater.* *Inter.* **11**, 39228–39237 (2019).

12. Ma, Y. et al. Liquid bandage harvests robust adhesive, hemostatic, and antibacterial performances as a first-aid tissue adhesive. *Adv.* *Funct.* *Mater.* **30**, 2001820 (2020).

13. Zou, H. et al. Quantifying the triboelectric series. *Nat*. *Commun*. **10,** 1427 (2019).

14. Wang, L. et al. A biocompatible cellulose-nanofiber-based multifunctional material for Fe^3+^ detection and drug delivery. *J*. *Mater*. *Chem*. *C* **8,** 11796−11804 (2020).
