# Supplementary material for: Cellulose nanofiber-mediated manifold dynamic synergy enabling adhesive and photo-detachable hydrogel for self-powered E-skin
Source: Nat Commun. 2024 May 8;15:3859. doi: 10.1038/s41467-024-47986-y (PMC11078967; doi:10.1038/s41467-024-47986-y)
Supplement: Supplementary file 3 — Description of Additional Supplementary Files [file 41467_2024_47986_MOESM3_ESM.pdf]

**Description of Additional Supplementary Files**

**File Name: Supplementary Data 1**

Description: The atomic coordinates of the Gaussian simulation optimized computational models among Fe ions ($Fe^{3+}$, $Fe^{2+}$) and gelatin, chitosan, alginate, starch, PAAm, and PVA. Fe (x), x represents the valence state of Fe ions, including Fe (III) and Fe (II).

**File Name: Supplementary Movie 1**

Description: Photo-detachable adhesion properties of the CNF-DA/PAA@$Fe^{3+}$ hydrogel. CNF-DA/PAA@$Fe^{3+}$ hydrogel seamlessly adheres to pocrine skin, and the hydrogel peels off easily after UV irradiation.

**File Name: Supplementary Movie 2**

Description: Adhesion properties of he CNF-DA/PAA@$Fe^{3+}$ hydrogel. The CNF-DA/PAA@$Fe^{3+}$ hydrogel can adhere to 20 g of steel balls. During the return movement, the steel balls are tightly bonded and do not fall off.

**File Name: Supplementary Movie 3**

Description: Conductivity properties of the CNF-DA/PAA@$Fe^{3+}$ hydrogel. A light-emitting diode (LED) can be lighted cyclically with a easy capacity when the CNF-DA/PAA@$Fe^{3+}$ hydrogel acts as an ionic conductor, proving it possesses excellent ionic conductivity and sensitivity.

**File Name: Supplementary Movie 4**

Description: Sensing properties of the CNF-DA/PAA@$Fe^{3+}$ hydrogel. When the CNF-DA/PAA@$Fe^{3+}$ hydrogel is adhered to the human wrist and bent, repeatable resistance responses with a high signal-to-noise ratio are also obtained, demonstrating a long service life and good reliability of hydrogel.

**File Name: Supplementary Movie 5**

Description: PdA-TENG properties of the CNF-DA/PAA@$Fe^{3+}$ hydrogel. When the CNF-DA/PAA@$Fe^{3+}$ hydrogel is assembled into a self-powered electronic skin, the light-emitting diode (LED) can easily cycle to emit light after continuous tapping of the sole of the foot, demonstrating its excellent electrical output performance.

**Supplementary Movie 6**

Description: Wireless-sensing properties of the CNF-DA/PAA@$Fe^{3+}$ hydrogel. When PdA-TENG is applied to wireless sensors, electrical signals of human body movement can be accurately collected.
