# Supplementary material for: Cellulose nanofiber-mediated manifold dynamic synergy enabling adhesive and photo-detachable hydrogel for self-powered E-skin
Source: Nat Commun. 2024 May 8;15:3859. doi: 10.1038/s41467-024-47986-y (PMC11078967; doi:10.1038/s41467-024-47986-y)
Supplement: Supplementary file 11 — Reporting Summary [file 41467_2024_47986_MOESM11_ESM.pdf]

nature portfolio

Corresponding author(s): He Liu, Chaoji Chen

Last updated by author(s): Feb 27, 2024

Reporting Summary

Nature Portfolio wishes to improve the reproducibility of the work that we publish. This form provides structure for consistency and transparency in reporting. For further information on Nature Portfolio policies, see our Editorial Policies and the Editorial Policy Checklist.

Statistics

For all statistical analyses, confirm that the following items are present in the figure legend, table legend, main text, or Methods section.

n/a	Confirmed	
☐	☒	The exact sample size (*n*) for each experimental group/condition, given as a discrete number and unit of measurement
☐	☒	A statement on whether measurements were taken from distinct samples or whether the same sample was measured repeatedly
☒	☐	The statistical test(s) used AND whether they are one- or two-sided<br>*Only common tests should be described solely by name; describe more complex techniques in the Methods section.*
☐	☒	A description of all covariates tested
☒	☐	A description of any assumptions or corrections, such as tests of normality and adjustment for multiple comparisons
☐	☒	A full description of the statistical parameters including central tendency (e.g. means) or other basic estimates (e.g. regression coefficient) AND variation (e.g. standard deviation) or associated estimates of uncertainty (e.g. confidence intervals)
☒	☐	For null hypothesis testing, the test statistic (e.g. *F*, *t*, *r*) with confidence intervals, effect sizes, degrees of freedom and *P* value noted<br>*Give P values as exact values whenever suitable.*
☒	☐	For Bayesian analysis, information on the choice of priors and Markov chain Monte Carlo settings
☒	☐	For hierarchical and complex designs, identification of the appropriate level for tests and full reporting of outcomes
☒	☐	Estimates of effect sizes (e.g. Cohen's *d*, Pearson's *r*), indicating how they were calculated

Our web collection on statistics for biologists contains articles on many of the points above.

Software and code

Policy information about availability of computer code

Data collection	SUNS (ver. UTM6503), Keithley (ver. 7510), Omnic (ver. 8.0)
Data analysis	OriginPro 2021 (ver. 9.8.0.200), Gaussian 09 (ver. 16 A.03), 2D shige (ver. 1.22)

For manuscripts utilizing custom algorithms or software that are central to the research but not yet described in published literature, software must be made available to editors and reviewers. We strongly encourage code deposition in a community repository (e.g. GitHub). See the Nature Portfolio guidelines for submitting code & software for further information.

Data

Policy information about availability of data

All manuscripts must include a data availability statement. This statement should provide the following information, where applicable:

- Accession codes, unique identifiers, or web links for publicly available datasets
- A description of any restrictions on data availability
- For clinical datasets or third party data, please ensure that the statement adheres to our policy

The data supporting the findings of this study are available within thearticle and its supplementary files. Any additional requests for information can be directed to, and will be fulfilled by, the corresponding authors. Source data are provided with this paper.

Research involving human participants, their data, or biological material

Policy information about studies with human participants or human data. See also policy information about sex, gender (identity/presentation), and sexual orientation and race, ethnicity and racism.

Reporting on sex and gender	The participant's sex and gender were not considered since they do not affect the results.
Reporting on race, ethnicity, or other socially relevant groupings	The participant's race, ethnicity, or other socially relevant groupings were not considered since they do not affect the results.
Population characteristics	The participant was randomly selected, which the selected participant was between 25 and 27 years old.
Recruitment	The participant was recruited randomly with no compensation. There was no self-selection bias for the recruitment that may impact experimental results. Recommend volunteers through the group and we discuss with volunteers.
Ethics oversight	Shanghai Ninth People's Hospital, Shanghai Jiao Tong University School of Medicine Ethics Commitee (SH9H-2022-T357-1)

Note that full information on the approval of the study protocol must also be provided in the manuscript.

Field-specific reporting

Please select the one below that is the best fit for your research. If you are not sure, read the appropriate sections before making your selection.

[x] Life sciences [ ] Behavioural & social sciences [ ] Ecological, evolutionary & environmental sciences

For a reference copy of the document with all sections, see nature.com/documents/nr-reporting-summary-flat.pdf

Life sciences study design

All studies must disclose on these points even when the disclosure is negative.

Sample size	No statistical methods were used to predetermine sample size. Sample size was based on preliminary experimentation. The sample size is sufficient to identify differences between groups. Details regarding sample size of all experiments are provided in the Methods section and Figure legends.
Data exclusions	No data were excluded from the analyses.
Replication	These measures were taken to verify the reproducibility, and all attempts at replication were successfully. The experiments were replicated or performed independently for 3-6 times in the manuscript, and 3 times in the supplementary information. The specific number of independent replicate experiments is indicated in the figure legend.
Randomization	All the experimental groups were randomly allocated.
Blinding	The investigators who assisted in data collection were blinded to group allocation during data collection and analysis.

Reporting for specific materials, systems and methods

We require information from authors about some types of materials, experimental systems and methods used in many studies. Here, indicate whether each material, system or method listed is relevant to your study. If you are not sure if a list item applies to your research, read the appropriate section before selecting a response.

Materials & experimental systems

n/a	Involved in the study	
[x]	[ ]	Antibodies
[ ]	[x]	Eukaryotic cell lines
[x]	[ ]	Palaeontology and archaeology
[x]	[ ]	Animals and other organisms
[x]	[ ]	Clinical data
[x]	[ ]	Dual use research of concern
[x]	[ ]	Plants

Methods

n/a	Involved in the study	
[x]	[ ]	ChIP-seq
[x]	[ ]	Flow cytometry
[x]	[ ]	MRI-based neuroimaging

Eukaryotic cell lines

Policy information about cell lines and Sex and Gender in Research

Cell line source(s)	L cell, L-929 were purchased from Wuhan Pricella Biotechnology Co., Ltd (CL-0137).
Authentication	None of the cell lines used were authenticated.
Mycoplasma contamination	All cell lines were tested negative for mycoplasma contamination.
Commonly misidentified lines (See ICLAC register)	No misidentified cell lines were used in our study according to the results of ICLAC register.

Plants

Seed stocks	None of the seed stocks or other plant material were used.
Novel plant genotypes	None of the novel plant genotypes were produced.
Authentication	None of the seed stocks used were authenticated.
